# Personalising anal cancer radiotherapy dose (PLATO): protocol for a multicentre integrated platform trial

**DOI:** 10.1136/bmjopen-2025-109655

**Published:** 2025-11-09

**Authors:** Russell Frood, Alexandra Gilbert, Duncan Gilbert, Natalie L Abbott, Susan D Richman, Vicky Goh, Sheela Rao, Joanne Webster, Alexandra Smith, Joanne Copeland, Sharon P Ruddock, Lindy Berkman, Rebecca Muirhead, Andrew G Renehan, Mark Harrison, Richard Adams, Maria Hawkins, Sarah Brown, David Sebag-Montefiore

**Affiliations:** 1 Leeds Institute of Clinical Trials Research, University of Leeds, Leeds, UK; 2 MRC Clinical Trials Unit, University College London, London, UK; 3 National Radiotherapy Trials QA, Velindre Cancer Centre, Cardiff, UK; 4 Leeds Institute of Medical Research, University of Leeds, Leeds, UK; 5 NIHR Leeds Biomedical Research Centre, Leeds NHS Teaching Hospitals, Leeds, UK; 6 Cancer Imaging, King’s College London School of Biomedical Engineering & Imaging Sciences, London, UK; 7 Gastrointestinal Unit, Royal Marsden NHS Foundation Trust, London, UK; 8 Patient Representative, London, UK; 9 Oxford University Hospitals NHS Foundation Trust, Oxford, UK; 10 Division of Cancer Sciences, University of Manchester, Manchester, UK; 11 Hillingdon Hospitals NHS Foundation Trust, Uxbridge, UK; 12 Velindre Cancer Centre, Cardiff, Wales, UK; 13 Medical Physics and Biomedical Engineering, University College London, London, UK

**Keywords:** Radiotherapy, Chemotherapy, Gastrointestinal tumours, Treatment Outcome, Clinical Trial, Adult radiotherapy

## Abstract

**Introduction:**

The incidence of anal carcinoma is increasing, with the current gold standard treatment being chemoradiotherapy. There is currently a wide range in the radiotherapy dose used internationally which may lead to overtreatment of early-stage disease and potential undertreatment of locally advanced disease.

PLATO is an integrated umbrella trial protocol which consists of three trials focused on assessing risk-adapted use of adjuvant low-dose chemoradiotherapy in anal margin tumours (ACT3), reduced-dose chemoradiotherapy in early anal carcinoma (ACT4) and dose-escalated chemoradiotherapy in locally advanced anal carcinoma (ACT5), given with standard concurrent chemotherapy.

**Methods and analysis:**

The primary endpoints of PLATO are locoregional failure (LRF)-free rate for ACT3 and ACT4 and LRF-free survival for ACT5. Secondary objectives include acute and late toxicities, colostomy-free survival and patient-reported outcome measures. ACT3 will recruit 90 participants: participants with removed anal tumours with margins ≤1 mm will receive lower dose chemoradiotherapy, while participants with anal tumours with margins >1 mm will be observed. ACT4 will recruit 162 participants, randomised on a 1:2 basis to receive either standard-dose intensity modulated radiotherapy (IMRT) in combination with chemotherapy or reduced-dose IMRT in combination with chemotherapy. ACT5 will recruit 459 participants, randomised on a 1:1:1 basis to receive either standard-dose IMRT in combination with chemotherapy, or one of two increased-dose experimental arms of IMRT with synchronous integrated boost in combination with chemotherapy.

**Ethics and dissemination:**

This study has been approved by Yorkshire & The Humber – Bradford Leeds Research Ethics Committee (ref: 16/YH/0157, IRAS: 204585), July 2016. Results will be disseminated via national and international conferences, peer-reviewed journal articles and social media. A plain English report will be shared with the study participants, patients’ organisations and media.

**Trial registration number:**

ISRCTN88455282.

**STRENGTHS AND LIMITATIONS OF THIS STUDY**

- An umbrella clinical trial allowing concurrent evaluation of treatment across distinct risk groups aiming to personalise radiotherapy dose for patients with locoregionally confined anal cancer.
- Integrated translational substudies enable correlation of biological, genomic and microbiome data with clinical outcomes using prospectively collected samples.
- Prospective collection of both clinician and patient-reported toxicity provides comprehensive assessment of treatment tolerability.
- Centralised radiotherapy quality assurance and standardised intensity modulated radiotherapy protocols ensure treatment consistency between sites.
- Recruitment is limited to ECOG 0–1 or 0–2 patients which likely restricts generalisability to frailer populations.

## Introduction

### Anal cancer

The global incidence of anal squamous cell carcinoma is rising with approximately 1600 new cases in the UK,^1^ with a global incidence of 50 865 cases.^2^ Around 90% of cases are associated with prior high-risk human papillomavirus (HPV) infection.^3^ Many patients present with local disease, with less than 10% presenting with metastases.^2^ The gold standard treatment is chemoradiotherapy (CRT); three randomised trials performed between 1987 and 1994 determined concurrent mitomycin C (MMC), 5-fluorouracil (5FU) and radiotherapy (MF-CRT) as the standard of care.^4–6^ ACT1, the largest of these, randomised 585 UK patients to radiotherapy alone or MF-CRT and demonstrated CRT significantly reduced locoregional failure (LRF) from 61% to 36% at 3 years.^4^ This benefit was maintained after a median follow-up of 13 years,^7^ leading to a change in routine clinical practice with chemoradiotherapy replacing radical surgery as the standard of care.^8^ The reduction in LRF was also demonstrated in a parallel European Organisation for Research and Treatment of Cancer (EORTC) trial of 110 patients with locally advanced disease^5^ and in a Radiation Therapy Oncology Group (RTOG) trial (n=310) which demonstrated a significant reduction in LRF colostomy-free survival (CFS) with MF-CRT compared with 5FU and radiotherapy.^6^

Three further randomised trials were performed between 1998 and 2008 testing the role of neoadjuvant, concurrent and adjuvant cisplatin schedules.^9–11^ The UK ACT2 trial, the largest anal trial,^11^ enrolled 940 patients and used a continuous two-phase radiotherapy schedule. There was no difference in the complete response rate for concurrent cisplatin compared with mitomycin C, and no improvement in progression-free survival (PFS) with the addition of two cycles of maintenance 5FU cisplatin. However, outcomes were improved compared with ACT1, and the continuous radiotherapy schedule may have contributed to this, as other trials included a treatment break prior to the second phase of radiotherapy. The RTOG 9811 trial of 641 patients found that neoadjuvant and concomitant cisplatin 5FU resulted in inferior LRF, CFS and overall survival (OS) when compared with MF-CRT.^9 10^ The ACCORD 03 trial enrolled 307 patients and showed no benefit from the addition of neoadjuvant cisplatin 5FU chemotherapy.^12^ It also compared two doses of boost (15 Gy vs 20–25 Gy) after whole pelvic irradiation. A non-significant 5% improvement in local control was seen. Following these trials, MMC 5FU CRT remained the standard of care.

The radiotherapy techniques used in previous phase III trials were relatively crude and resulted in substantial irradiation of the surrounding tissue and associated acute and late toxicity. Significant improvement in radiotherapy treatment using intensity modulated radiotherapy (IMRT) allows the use of altered doses to the gross tumour volume, sparing normal tissues.^13^ As IMRT can reduce acute toxicity, this has led to a wide range in the radiotherapy dose fractionation used, with doses of 60 Gy or more used in several countries globally. This is likely to result in substantial overtreatment.^14 15^

Although there are clinically derived prognostic biomarkers (T and N stage, male gender, low Hb and more recently tumour-infiltrating lymphocytes) which can help define the risk of relapse, there is currently a lack of biologically derived and validated predictive biomarkers to stratify and personalise treatment.^16–20^

We designed a UK umbrella clinical trial to personalise radiotherapy dose across the locoregional disease spectrum.

## Methods and analysis

### Design and aim

PLATO is an integrated umbrella trial protocol that employs a master protocol consisting of three risk stratified multicentre trials (ACT3, ACT4 and ACT5). ACT3 is a non-randomised phase II trial where participants with a locally excised small (<=2 cm) anal verge tumour with margins ≤1 mm receive lower dose chemoradiotherapy and participants with anal tumour margins >1 mm are observed. ACT4 is a randomised non-comparative phase II trial where participants are randomised on a 1:2 basis to receive either standard-dose IMRT or reduced-dose IMRT in combination with chemotherapy. ACT5 is a randomised comparative phase II/III trial with an internal pilot where participants are randomised on a 1:1:1 basis to receive either standard-dose IMRT in combination with chemotherapy, or one of two increased-dose experimental arms of IMRT with synchronous integrated boost (SIB) in combination with chemotherapy. An integrated translational programme is included across the trial platform.

**Figure 1:**
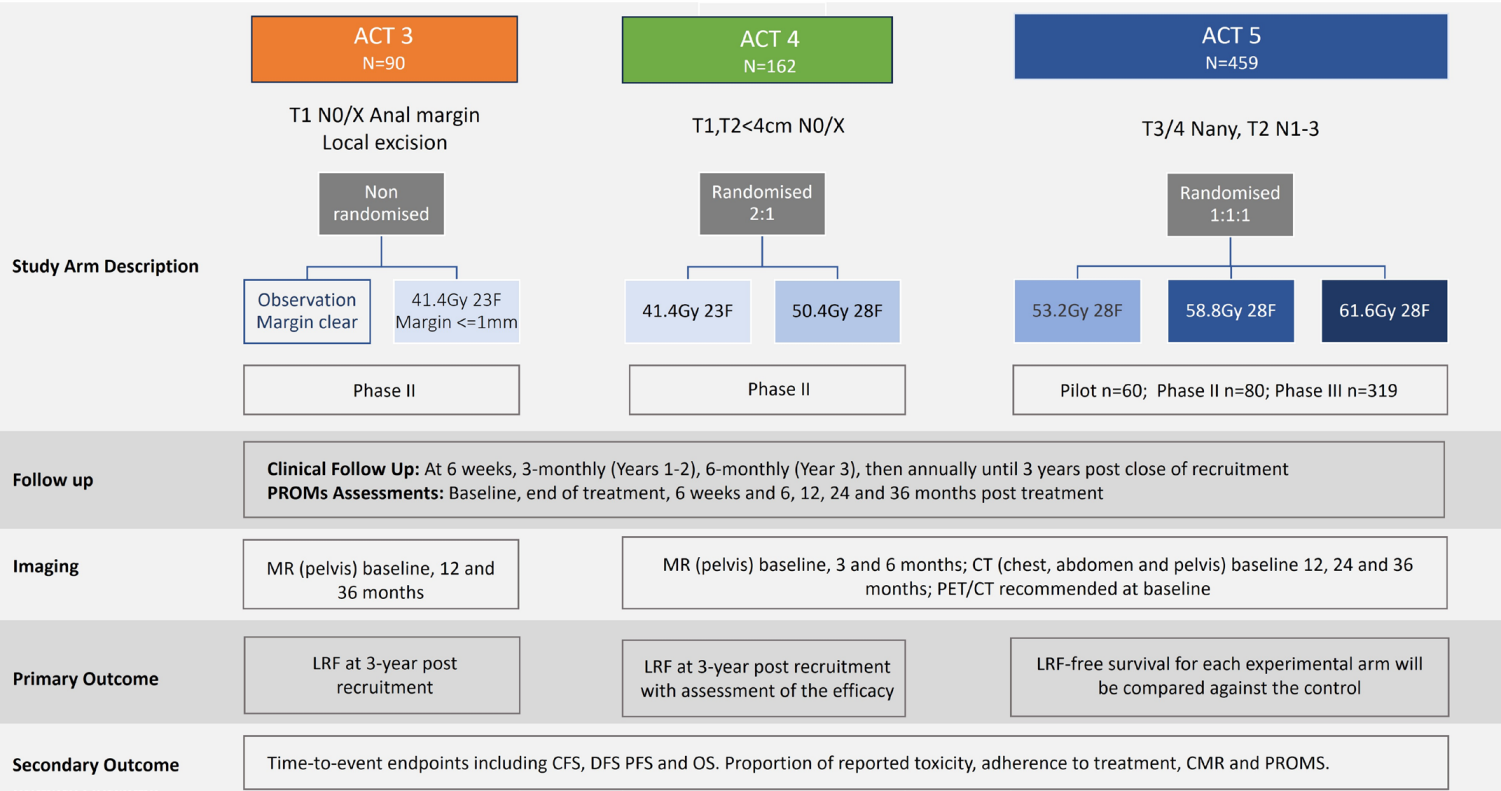
Overview of the participant pathway for ACT3, ACT4 and ACT5. CFS, colostomy-free survival; DFS, disease-free survival; CMR; complete metabolic response; LRF, locoregional failure; OS, overall survival; PFS, progression-free survival; PROMs, patient reported outcome measures.

A total of 711 eligible participants will be recruited from 36 UK National Health Service sites: 90 to ACT3, 162 to ACT4 and 459 to ACT5. Participant pathway is depicted in figure 1. The protocol (current V.9.0) and this manuscript have been written in accordance with the Standard Protocol Items: Recommendations for Interventional Trials guidelines. Recruitment is anticipated to take place over 6 years. Recruitment opened on 3 January 2017 and closed on 31 August 2023 with a total of 709 patients recruited. Follow-up for the primary endpoint analysis of each trial is currently ongoing, and the first primary endpoint results are expected to be reported in May 2026.

### Trial objectives

The primary objectives are as follows:

*ACT3:* To assess 3-year LRF-free rate in participants with anal margin tumours treated with local excision.

*ACT4:* To assess 3-year LRF-free rate in participants with early-stage disease.

*ACT5:* to assess LRF-free survival in participants with locally advanced disease.

#### Secondary objectives

To assess overall (all ACT trials) and between individual trial treatment arms (ACT4 and 5 only):

- Acute and late toxicities.
- Treatment compliance.
- Clinical response rate.
- Disease-free survival (DFS).
- Colostomy-free survival (CFS).
- Progression-free survival (PFS).
- Overall survival (OS).
- Patient-reported outcome measures.

#### Translational objectives

To assess the prognostic utility of p16/HPV status and tumour-infiltrating lymphocyte scores with respect to chemoradiotherapy in anal squamous cell carcinoma.

To investigate the nature and performance characteristics of circulating markers of disease (circulating tumour cells and cell-free DNA) in response/relapse in high-risk cases (ACT5 patients only).

To assess the changes in the gut microbiome caused by chemoradiotherapy and develop hypotheses around any potential relationship between this and patients experiencing late (gut) toxicities.

### Study population

Patients with locally excised T1 N0 or Nx anal margin tumour and anal canal superficially invasive squamous cell carcinoma (SISCCA) are eligible for ACT3, patients with T1−2 ≤4 cm N0 or Nx anal canal or T2 ≤4 cm N0 or Nx anal margin (in situ or treated by prior local excision within 3 months before randomisation) are eligible for ACT4, and patients with T2 N1-3 or T3-4 Nany are eligible for ACT5. ACT3 accepts patients with an Eastern Cooperative Oncology Group (ECOG) performance status of 0–2, whereas for ACT4 or ACT5, patients require an ECOG performance status of 0–1.

Only participants meeting all the inclusion criteria and none of the exclusion criteria will be considered for participation in the trial, with no eligibility waivers permitted. The full inclusion and exclusion criteria for ACT3, ACT4 and ACT5 are set out in tables 1 and 2.

**Table 1:** Inclusion criteria for the different PLATO studies

Inclusion criteria		
**ACT3**	**ACT4**	**ACT5**
Informed consent	Informed consent	Informed consent
Histologically proven, invasive primary squamous cell carcinoma of the anus	Histologically proven, invasive primary squamous cell carcinoma of the anus	Histologically proven, invasive primary squamous cell carcinoma of the anus
ECOG performance status 0–2	ECOG performance status 0–1	ECOG performance status 0–1
Absolute neutrophil count >1.5×10^9^/L; platelets >100×10^9^/L	Absolute neutrophil count >1.5×10^9^/L; platelets >100×10^9^/L 3	Absolute neutrophil count >1.5×10^9^/L; platelets >100×10^9^/L
Serum transaminase <2×ULN	Serum transaminase <2×ULN	Serum transaminase <2×ULN
Estimated GFR >50 mL/min	Estimated GFR >50 mL/min	Estimated GFR >50 mL/min
Bilirubin <1.5×ULN	Bilirubin <1.5×ULN	Bilirubin <1.5×ULN
HIV negative or HIV positive and receiving effective antiretroviral therapy with supervision and CD4 count >200	HIV negative or HIV positive and receiving effective antiretroviral therapy with supervision and CD4 count >200	HIV negative or HIV positive and receiving effective antiretroviral therapy with supervision and CD4 count >200
T1 N0 or Nx anal margin tumour and anal canal SISCCA treated with local excision within 3 months before registration	T1−2≤4cm N0 or Nx7 anal canal or T2≤4cm N0 or Nx7 anal margin (in situ or treated by prior local excision within 3 months before randomisation)	T2 N1-3 or T3-4 N any
Age 16 or over	Age 16 or over	Age 16 or over (18 in Ireland)
Patient considered fit for either the protocol-defined follow-up, or reduced dose CRT	Patient considered fit for all ACT4 protocol defined treatments	Patient considered fit for all ACT5 protocol defined treatments
Prepared to practise methods of contraception of proven efficacy during treatment and until 6 months post end of treatment	Prepared to practise methods of contraception of proven efficacy during treatment and until 6 months post end of treatment	Prepared to practise methods of contraception of proven efficacy during treatment and until 6 months post end of treatment
Able to undergo all mandated staging and follow-up investigations, including MRI	Able to undergo all mandated staging and follow-up investigations, including MRI	Able to undergo all mandated staging and follow-up investigations, including MRI

CD, cluster of differentiation; CRT, chemoradiotherapy; ECOG, Eastern Co-operative Oncology Group; GFR, glomerular filtration rate; SISCCA, superficially invasive squamous cell carcinoma.

**Table 2:** Exclusion criteria for the different PLATO studies

Exclusion criteria		
**ACT3**	**ACT4**	**ACT5**
Previous malignancy of pelvic origin where treatment was completed less than 2 years before registration or there is still evidence of disease, or previous untreated malignancy of any origin.	Previous malignancy of pelvic origin where treatment was completed less than 2 years before randomisation or there is still evidence of disease, or previous untreated malignancy of any origin.	Previous malignancy of pelvic origin where treatment was completed less than 2 years before randomisation or there is still evidence of disease, or previous untreated malignancy of any origin.
Prior systemic chemotherapy for anal cancer	Prior systemic chemotherapy for anal cancer	Prior systemic chemotherapy for anal cancer
Prior radiotherapy to the pelvis	Prior radiotherapy to the pelvis	Prior radiotherapy to the pelvis
Uncontrolled cardiorespiratory comorbidity	Uncontrolled cardiorespiratory comorbidity	Uncontrolled cardiorespiratory comorbidity
Pregnant or lactating	Pregnant or lactating	Pregnant or lactating
Immunocompromised (organ transplant)	Immunocompromised (organ transplant)	Immunocompromised (organ transplant)
Requiring ongoing treatment with a contraindicated concomitant medication	Requiring ongoing treatment with a contraindicated concomitant medication	Requiring ongoing treatment with a contraindicated concomitant medication
>1 local excision performed, at different times, for the same lesion		

#### Consent and withdrawal

Informed written consent will be given by all participants before entering the study and before any interventions. An example of the consent form is provided in online supplemental material 3. Consent will be taken by the principal investigator or their approved appropriate medically qualified healthcare professional. Optional consent for participants recruited to ACT5 will be sought regarding the participation in the biological serum plasma biomarker and microbiome substudies. In the event of withdrawal, any data collected up until that point will be kept and potentially included in analyses. It will be made clear to any participant withdrawing consent that further data pertaining to safety will continue to be collected.

10.1136/bmjopen-2025-109655.supp3 Supplementary data

### Registration, randomisation and recruitment

Participants will be registered (ACT3) or randomised (ACT4, ACT5) at the University of Leeds Cancer Research UK Clinical Trials Unit (CTU) following confirmation of written consent and eligibility.

#### ACT3

Participants with anal tumour margins >1 mm (deep and lateral) will undergo observation. Participants with anal tumour margins ≤1 mm (close or involved), including piecemeal excision, will receive lower dose CRT.

#### ACT4

Participants will be randomised on a 1:2 basis (standard-dose:reduced-dose) to receive either standard-dose IMRT or reduced-dose IMRT, both in combination with chemotherapy. A computer-generated minimisation program that incorporates a random element will be used to ensure the treatment groups are well-balanced for the following participant characteristics:

- T-stage (T1, T2).
- N-stage (N0, NX).
- Gender (M, F)
- HIV status (positive, negative).
- Randomising centre.

#### ACT5

For each of the pilot, phase II and phase III trial components, patients who fulfil the eligibility criteria and have given written informed consent will be randomised on a 1:1:1 basis to receive either standard-dose IMRT in combination with chemotherapy, or one of two increased-dose experimental arms of IMRT with SIB in combination with chemotherapy and will be allocated a trial number. A computer-generated minimisation program will be used to balance the following participant characteristics at randomisation:

- T-stage (T2/3, T4).
- N-stage (NX/0/1, N2/3).
- Gender (M, F).
- HIV status (positive, negative).
- Chemotherapy regimen (5FU, capecitabine).
- Randomising centre.

### Sample size

The target sample size is 711 participants across each of the three study arms. The justification for the translational research questions sample size is provided in online supplemental material 1.

10.1136/bmjopen-2025-109655.supp1 Supplementary data

#### ACT3

This study is powered to demonstrate that the overall treatment strategy of local excision alone with or without additional lower dose radiotherapy with chemotherapy does not produce unacceptable rates of efficacy (ie, below 80%) but can reach efficacy rates of up to 90% (ie, LRF rate of 10%). With 80% power and 5% one-sided significance, 82 patients are required. Allowing for a dropout rate of 10%, the target sample size is 90 patients, recruited over 3 years.

#### ACT4

This study is powered to demonstrate that the reduced-dose IMRT arm does not produce unacceptable rates of efficacy (ie, below 80%) but can reach efficacy rates of up to 90% (ie, LRF rate of 10%). A target efficacy rate of 90% or greater is considered desirable based on data from the ACT2 trial,^10^ where 10% of T1-2 N0 patients had a pelvic failure at 3 years post-randomisation. The standard-dose IMRT arm will act as a calibration arm to ensure the desired efficacy rate is plausible.

With 80% power and 5% one-sided significance, 123 patients are required with a 1:2 randomisation (standard-dose:reduced-dose). In order to address the research hypothesis for the p16+ subset, the sample size has been inflated by 20% (as 90% of patients are expected to present with this genotype, and a further 90% have samples suitable for analysis). Allowing for a 10% dropout rate, the target sample size is 162 patients, recruited over 2 years.

#### ACT5

The pilot stage of ACT5 will recruit a maximum of 60 patients (20:20:20). A formal power calculation was not performed for the pilot study given the aim is to review the initial feasibility of dose escalation and concomitant chemotherapy.

The phase II trial will recruit an additional 80 patients. The phase II analysis will focus on the acute toxicity data including grade 3 and above neutropenia from the first 140 patients (including the pilot) randomised (overall in the three arms). With 80% power and 5% one-sided significance, an acceptable rate of grade 3/4 neutropenia of <40% and an unacceptable rate of ≥60% yields a sample size of 126 patients (42:42:42), 140 with 10% drop out. The desired rate of 40% is based on data from the RTOG 9811, RTOG 0529 and ACT2 trials where rates of grade 3/4 neutropenia were 45%, 58% and 24%, respectively.

The overall trial is powered on detecting an absolute difference of 10% in 3-year LRF-free survival between the control and experimental arm(s). This difference is equivalent to a HR of 0.63 and assumes a LRF rate of 30% (3-year LRF-free survival 70%) in the control arm based on the ACT2 data.^10^

If both experimental arms pass the prespecified boundaries for acceptable toxicity at interim analysis, all three arms will continue into the phase III trial. This would compare each experimental arm against the standard dose in terms of 3-year LRF-free survival. With 80% power and 10% two-sided significance, 459 (153:153:153) patients are required (including 10% drop out) for the primary endpoint analysis. The target event rate is 113 per comparison based on assuming 6 years of recruitment and a minimum of 3 years follow-up for all patients.

### Treatment regimen

#### Radiotherapy treatment outlining and planning

Participants will have their radiotherapy planning study performed using a CT acquisition with a slice thickness of no greater than 3 mm in a supine position. Patients will be scanned with a comfortably full bladder using local bladder filling protocol. The clinician will determine if additional bolus material is required during scanning. A nationally approved consensus protocol was developed for IMRT (online supplemental material 1).

### Treatment regimen

#### ACT3

Participants within the observation arm will be observed under local practice but must attend trial follow-up assessments. Patients under the intervention arm will receive reduced dose IMRT (CTV 41.4 Gy in 23 fractions (F)) plus MMC capecitabine (online supplemental material 2).

10.1136/bmjopen-2025-109655.supp2 Supplementary data

#### ACT4

Participants are randomised to receive either to receive either standard-dose IMRT (GTV 50.4 Gy, Elective CTV 40 Gy (28F)) in combination with chemotherapy or reduced-dose IMRT (GTV 41.4 Gy, Elective CTV 34.5 Gy (23F)) in combination with chemotherapy (online supplemental material 2).

#### ACT5

Patients will receive either standard-dose IMRT (GTV 53.2 Gy) or one of two increased-dose experimental arms of IMRT with SIB (GTV 58.8 Gy or GTV 61.6 Gy) with elective CTV treated to 40 Gy (28F) in combination with chemotherapy. ACT5 will use mitomycin C combined with either capecitabine or 5FU based on hospital preference (online supplemental material 2).

### Trial assessments and follow-up

For an overview of the ACT3, ACT4 and ACT5 assessment schedule, see online supplemental material 2.

#### Clinician-reported toxicity

With the exception of the ACT3 observation arm, assessment of acute toxicities will take place during each week of treatment. In ACT5 only, clinician-reported acute toxicities will be captured at 6 weeks, 3 and 6 months post-end of treatment. All adverse reactions, serious adverse reactions and related unexpected serious adverse events will be recorded in ACT5, in ACT 4 grade 2 and above will be recorded and in ACT3 grade 3 and above will be recorded.

#### Patient-reported outcome measures (toxicity and quality of life)

Baseline, acute and late toxicities and assessment of participants’ quality-of-life (QoL) will be performed via the validated EORTC QLQ-C30 and QLQ-ANL27^21^ questionnaires at baseline, in the final week of treatment, at 6 weeks, 6, 12, 24 and 36 months post-end of treatment. ACT3 participants in the observation arm will complete QoL questionnaires at 6 weeks, 6, 12, 24 and 36 months post-date of registration. Patients will all complete baseline and final week of treatment questionnaires on paper and then could opt to complete on paper or online using REDCAP.

#### Follow-up imaging

For ACT3, an MRI pelvis will be carried out at 12 and 36 months post-treatment.

For ACT4 and ACT5, MRI pelvis will be performed at 3 and 6 months, and a follow-up CT (chest/abdomen/pelvis) will be carried out at 12, 24 and 36 months post-treatment.

#### Assessment of efficacy

All patients will be followed up to detect recurrence that may occur in the pelvis or as distant metastases. These will be recorded on the relevant CRF and completed at the time of each follow-up visit. All failures that occur within the pelvis up to the level of the sacral promontory are considered locoregional. The date of failure will be taken as the date of the biopsy, or where this is not available, the date of the first imaging assessment that confirmed the LRF. The presence of distant metastases is determined by cross-sectional imaging.

DFS is defined as the time from randomisation to the first documented evidence of pelvic failure which includes LRFs, distant metastases, new primary tumours or death.

All deaths occurring from the date of registration/randomisation will be recorded.

#### Ancillary care

Participants will receive ancillary care beyond the scope of the trial as required. Any adverse events or complications arising from trial participation will be promptly addressed, and necessary medical interventions will be provided at no cost to the participants. Ancillary care will extend to managing conditions unrelated to the trial that may arise during the study period, ensuring the overall well-being of the participants.

#### Translational research

Baseline and pretreatment biopsies, and resections and biopsies from recurrent disease will be collected retrospectively for participants. Tumours will be characterised for p16 (immunohistochemistry), HPV status (PCR) and tumour-infiltrating lymphocyte scores, and correlated with outcome.

Longitudinal blood (plasma) samples (ACT 5) (pretreatment, end of treatment, 3 months, 6 months and 12 months post-treatment and at relapse) will be used for analysis of cell-free DNA (cfDNA) and characterisation and changes in levels of circulating tumour cells and comparison of these with conventional markers of response.

At least 65% of the ACT5 participants will have faecal samples collected prior to commencing chemoradiotherapy and a paired sample 6–12 weeks (±4 weeks) after the end of treatment. The microbiome will be characterised in these samples and will allow for additional biological insights into local immune response and potential factors relating to long-term bowel toxicity.

### Data collection and management

Participant data will be recorded on trial-specific case report forms and submitted to the CTU from participating sites. All data will be entered, validated and stored securely by CTU, with missing and discrepant data queried by the data management team. Missing data will be followed up until received or confirmed not available. Data items regarding consent, patient safety and the primary endpoint will be subject to manual priority checking.

Only the CTU will have access to the data, prior to analysis and release of trial results. On completion of the trial, data will be stored in the sponsor archiving facility for a minimum of 15 years. After the final trial results have been published, interested researchers may contact the CTRU to request access to relevant data.

### Statistical analysis

Efficacy analyses for ACT3 and ACT5 will be on an intention-to-treat (ITT) basis, including participants according to the treatment arm they were recruited/randomised to. For ACT4, a modified ITT will be used, including participants if they receive at least one dose of trial treatment.

### Primary endpoint analysis

LRF includes a failure at the primary site (local) and/or surrounding nodal sites (regional). The event date is the biopsy date where the failure was confirmed histologically, or the scan date used to confirm failure on MDT review if a biopsy date is not available.

In ACT3, analysis will assess the proportion of participants without a LRF at 3-year post-recruitment, and the one-sided 95% CI (corresponding to a two-sided 90% CI) on the whole trial cohort. Assessment of the efficacy of the overall treatment strategy will focus on the lower bound of the CI, and whether it falls below the ‘unacceptable’ rate of 80%.

In ACT4, analysis will assess the proportion of participants without a LRF in the reduced-dose IMRT arm at 3 years post-recruitment and the one-sided 95% CI. Assessment of the efficacy will focus on whether the lower bound of the CI falls below the ‘unacceptable’ rate of 80%. The proportion of participants without a LRF in the standard-dose IMRT arm with corresponding CI will be presented to act as a calibration arm, and to establish whether the desired efficacy rate of 90% is plausible. In addition, the analysis will be performed on the subgroup of patients with a p16+ genotype.

In ACT5, LRF-free survival for each experimental arm will be compared against the control, that is, two separate comparisons, using Cox’s proportional hazards (PH) modelling, adjusting for the minimisation factors. The HR for the experimental arm versus the control arm will be presented along with two-sided 90% CIs and associated p value testing. A secondary analysis will compare the combined dose escalation arms with the control to test the hypothesis that dose escalation generally provides improved LRF-free survival compared with control.

LRF-free survival will be presented via Kaplan-Meier (KM) curves. Median LRF-free survival and LRF-free survival estimates at 3 years, with corresponding 90% and 95% CIs will be presented along with the log-rank test statistic (and associated p value).

Participants who are failure-free at the time of analysis, or who have come off trial prior to observing their primary endpoint, will be censored at their last known alive and LRF-free date.

#### Secondary endpoint analysis

Time-to-event endpoints including CFS, DFS, PFS and OS will be presented using KM curves. For ACT5, Cox’s PH model will be used to compare each experimental arm with the control. There will be no formal comparison between the arms for ACT3 and ACT4. The proportion of participants experiencing each event will be presented by treatment group (where applicable) and overall.

The proportion of participants experiencing acute toxicities as per the latest National Cancer Institute Common Terminology Criteria for Adverse Events (NCI-CTAE) will be summarised for each treatment arm, for the overall treatment period and (for ACT5 only) at 6 weeks and 3 and 6 months. The number and proportion of participants experiencing grade 3 and above acute toxicities will be reported along with 95% CIs. These will be compared between each treatment arm and the control using a multivariable logistic regression model adjusted for the minimisation factors, for ACT5 only. Exploratory comparisons of acute toxicity may be performed between the experimental arms.

The proportion of participants adhering to the radiotherapy schedule (with no greater than 3 days delay due to toxicity) will be reported. In ACT5, only this will be compared between each treatment arm and the control using a logistic regression model.

The proportion of participants achieving a complete response (CR) at 3 and 6 months will be presented along with 95% CIs (ACT4 and 5). Complete response is defined as a TRG score of 1 or 2 assessed via MRI, or if clinical assessment only is used to define response, a category of ‘Complete response’. A logistic regression model, adjusted for the minimisation factors, will be used to compare each treatment arm with the control (ACT 5 only) for the proportion of participants who have and have not achieved a CR at 6 months post-end of treatment.

EORTC QLQ guidelines will be used for analyses and management of missing data.^1^ Mean scores and change in mean score from baseline with corresponding 95% CIs will be calculated for all domains of the patient reported questionnaires (EORTC QLQ-C30 and ANL27) for each treatment group and overall, at each follow-up timepoint.

Descriptive summaries will be presented by treatment arm for the pattern of failure and the proportion of participants with local relapse undergoing salvage surgery.

### Translational statistical analysis

#### Analysis of p16, HPV and LRF

p16/HPV and tumour-infiltrating lymphocytes will be analysed as part of the main trial analysis (primary endpoints of 3 year LRF) and as such, results of the biomarker analysis from ACT4 and the pilot/phase II stages of ACT5 will be made available (subject to oversight, see below) with a plan to validate the predictive value in later stages of the platform and inform subsequent biomarker stratification in later stages of the trial.

#### Analysis of cfDNA

Serial data on CTCs and cfDNA will be analysed for the cohort of patients in ACT5 (with an estimated 25% risk of recurrence at 1 year). Performance characteristics will be determined for these data (CTC and cfDNA) in relation to complete response and 1-year DFS.

#### Analysis of changes in gut microbiome

The faecal microbiome in patients undergoing radical treatment for anal cancer has not previously been investigated, and as such, all analyses and comparisons with, for example, long-term toxicity outcome measures are exploratory. Our primary outcome measure is the feasibility of collecting and analysing the faecal microbiome in patients enrolled in ACT5 with respect to changes in microbiome/metabolomics profiles before and after chemoradiotherapy.

### Trial oversight

The chief investigator (CI) will have overall responsibility for the trial design, set-up and management and will be supported by five study-specific clinical Leads across the three trials. A trial management group (TMG) comprising the CI, CTU team, a PPIE representative (LB), co-leads for each trial, representatives for medical oncology, medical imaging, radiotherapy QA, translational research and patient reported outcomes and key external members of staff will meet 3–6 monthly. A trial steering committee will provide overall supervision of the trial, in particular trial progress, adherence to protocol, participant safety and consideration of new information. It will include an independent chair, not less than two other independent members and a patient and public involvement (PPI) representative. An independent data monitoring and ethics committee (DMEC), which will meet annually as a minimum, will review safety and ethics of the trial during recruitment and follow-up period.

### Patient and public involvement and engagement (PPIE)

Input from the Leeds Cancer Centre PPIE team, National Cancer Research Institute consumer representatives and Leeds Radiotherapy Research user group was sought, and the information gained acted on through every aspect of the protocol design, the patient information leaflet and the lay summary.

### Ethics and dissemination

This study has been approved by the Yorkshire & The Humber – Bradford Leeds Research Ethics Committee (ref: 16/YH/0157, IRAS: 204585), July 2016.

The trial results will be analysed in stages for short-term and long-term endpoints. Results will be disseminated via national and international conferences, peer-reviewed journal articles as well as social media and a newsletter to the relevant stakeholders. The final trial publications will be written by the TMG and will adhere to International Committee of Medical Journal Editors guidelines.

## Discussion

The incidence of anal carcinoma is rising globally, with a range of radiotherapy doses being employed to treat patients without high levels of evidence. PLATO is an integrated umbrella trial which aims to personalise radiotherapy dose for locoregionally confined cancer with arms looking at anal margin tumours, early and advanced anal carcinoma with it being the multicentre phase II (ACT3 and ACT4) and phase II/III (ACT5) to do so. It is also the first anal cancer trial to use the EORTC QLG-ANL27 questionnaire and has translational components of the study for a subset of patients will add insight into HPV status and circulating tumour markers on DFS, and the changes in the gut microbiome and how this may relate to bowel toxicity.

Since the development of the protocol, the core outcome research measures in anal cancer (CORMAC)^22^ has been published with the outcomes in the original protocol being retained but the planned analyses will align with the CORMAC recommendations. PLATO is focused on the personalisation of treatment for patients with a good ECOG performance status and highlights the need for further research into the optimal treatment for elderly and frail patients.^23^ As part of this, a need for a reliable surrogate for the current performance status which is not subjective and can take into account changes in patients physical state is needed.^24^ Medical imaging-based body composition analysis, which is the process of quantifying the volume, density and ratio of adipose tissue, skeletal muscle, bone and soft tissues within the abdomen, can provide additional valuable patient-specific metrics which could be used as an indicator of frailty or outcome.^25^ Future translational work looking into the imaging biomarkers as part of the PLATO study will be explored and will be invaluable in any future trial design.

## Supplementary Material

Reviewer comments

Author's manuscript
