# Supplementary material for: Personalising anal cancer radiotherapy dose (PLATO): protocol for a multicentre integrated platform trial
Source: BMJ Open. 2025 Nov 9;15(11):e109655. doi: 10.1136/bmjopen-2025-109655 (PMC12658545; doi:10.1136/bmjopen-2025-109655)
Supplement: Supplementary data [file bmjopen-15-11-s003.pdf]

Delete text and insert local header here

Patient Information Sheet and Informed Consent Document

PLATO

Anal Cancer Trial 5 (ACT5)

A large-print version of this sheet is available on request

We invite you to take part in a research study called ACT5 (Anal Cancer Trial 5)

- Before you decide whether to take part, it is important for you to understand why the research is being done, what it would involve for you and how we would use the information we would collect about you.
- Please take time to read the following information carefully. Talk to others about the study if you wish.
- Ask us if anything is unclear, or if you would like more information.
- Once you have read this information, your doctor or nurse will talk to you about the study again and you can ask any questions you like.
- **Part 1** (Sections 1-22) tells you the purpose of the study and what will happen if you take part.
- **Part 2** (Sections 23-33) gives you more detailed information about the conduct of the study.
- Please take time to decide whether or not you wish to take part.

Thank you for reading this information sheet.

How to contact us

If you have any questions about this study, or need to speak to a member of your medical care team, please contact:

<<Enter PI, nurse name >>

<< Contact details for site>>

In case of emergencies outside of office hours, please contact:

<<Please insert relevant numbers for emergency contacts>>

Frood R, et al. BMJ Open 2025; 15:e109655. doi: 10.1136/bmjopen-2025-109655

Part 1

Important things that you need to know

- We want to find out the best way to treat patients with locally advanced cancer of the anus.
- The study will compare 3 different doses of radiotherapy given with chemotherapy (chemoradiotherapy).
- You will need to attend hospital every week day for treatment (for 5½ weeks), then for follow-up assessments every 3 months (for 2 years) and every 6 months (for 1 year), when we will collect information about your progress.
- You can stop taking part in the study at any time, without giving a reason. This will not affect the standard of care you receive in any way.

1 What is the purpose of the study?

In this study we are exploring the potential benefit of giving higher doses of radiotherapy to the tumour in patients with more advanced anal cancers. An advanced cancer is one that is bigger than 4cm or has spread into the local lymph nodes.

Previous research has shown that patients with a more advanced cancer have a higher chance of the cancer coming back.

We are testing to see if giving a higher dose of radiotherapy reduces the chance of the cancer coming back, while at the same time not causing too many extra side effects.

2 Why have I been chosen?

Your Doctor or Surgeon has informed you that you have a locally advanced anal cancer that may be successfully treated with a combination of chemotherapy and radiotherapy.

The study aims to recruit 459 patients like you.

3 Do I have to take part?

No, your participation in this study is entirely voluntary. If you decide to take part, you will be given this information document to keep. You will be asked to sign a consent form, but you may withdraw your consent to take part at any time and without giving a reason.

If you decide not to take part, your doctor or nurse will be happy to talk through other treatment alternatives with you. The standard of your treatment and care will not be affected in any way if you decide not to take part.

4 If I want to, will I definitely be able to take part?

Unfortunately, no. Although your doctor thinks you might be suitable to take part, they will still need to carry out some tests and ask you some specific questions to make sure you are suitable. These are known as "eligibility screening tests". Most of these tests would happen as a part of normal clinical care, and you may already have undergone some of them. These tests are described under Section 8.

If the eligibility screening tests show that it is not appropriate for you to take part in this study, your doctor will discuss your other treatment options with you.

Frood R, *et al. BMJ Open* 2025; 15:e109655. doi: 10.1136/bmjopen-2025-109655

5 What is the current standard treatment?

The standard treatment for locally advanced anal cancer is a combination of chemotherapy (anti-cancer drugs) and radiotherapy (high energy X-rays). The aim of this treatment is to get rid of the cancer.

6 What are the new treatments that are being studied?

The new treatments which are being studied are two higher doses of radiotherapy, given in combination with standard chemotherapy. We want to find out whether a higher dose of radiotherapy offers a better chance of getting rid of the cancer, while not causing unacceptable side effects. The higher doses of radiotherapy being tested take no longer to give each day and are given over the same number of days as the standard dose (5½ weeks). The only difference is that the dose given each day is slightly higher than the standard dose. We expect that the higher doses will lead to an increase in side effects at the time of radiotherapy and afterwards (see Section 11), but it may reduce the number of cancers that come back.

7 Which treatment will I receive if I take part?

We need to make sure the groups of people receiving each treatment are as similar as possible, to make sure that the way each group responds to their treatment is due to the treatment itself rather than some other difference between the groups that we do not know about. The best way of dividing people up into groups is by randomly deciding which treatment each person will receive. This is called ‘randomisation.’ A computer programme will be used to decide what treatment you will be randomised to receive. Neither you, your doctor nor the study staff will be able to choose which group you will be placed in, or which radiotherapy dose you will receive. In this way, a fair comparison between the treatments can be made.

In this study, participants will be split into three groups. Each group will receive a different dose of radiotherapy

If you are in the control group, you will receive the standard dose of radiotherapy. If you are in the experimental groups, you will receive one of the two higher doses of radiotherapy.

You will have a 2 in 3 chance of receiving a higher dose of radiotherapy and a 1 in 3 chance of receiving the standard dose. You will also be given chemotherapy alongside your radiotherapy treatment. The chemotherapy that you will receive will be the same no matter which dose of radiotherapy you receive. Further details about the treatments are given under Section 8.

Occasionally, some patients will be advised to have a surgical procedure before starting treatment if the medical team think that diarrhoea side effects or control of your bowels will become a problem during the treatment. The surgical procedure will form a stoma (called an ileostomy or colostomy). A stoma is an opening on your abdomen or tummy that allows faeces to bypass your back passage and pass straight into a bag. This approach is considered before treatment in routine practice in the same way as this study. Your doctor will explain more about this procedure if it is relevant to you.

Frood R, *et al. BMJ Open* 2025; 15:e109655. doi: 10.1136/bmjopen-2025-109655

8 What will happen to me if I take part?

This section describes what will happen to you if you agree to take part in the study.

Routine baseline assessments

You will need to have some tests, examinations and scans of your disease, to make sure that you are suitable for the study. Most of these are routine and include:

- Medical history and physical examination.
- A biopsy (a procedure where a small piece of tissue is removed from the cancer to look at under a microscope).
- Blood tests to check your blood count, kidney and liver function. We will also test you for Human Immuno-deficiency Virus (HIV).
- Scans to assess your disease. This will include a CT scan (which involves exposing you to some radiation) of your chest, abdomen and pelvis, and an MRI scan of your pelvis. Your doctor may also choose for you to have a PET scan of your whole body, which may be considered additional to normal care at some hospitals. More information about these scans is provided later (Section 17).

An assessment of your quality of life

If you agree to take part, we would like you to fill in a questionnaire about your quality of life and how you are feeling. This questionnaire will take about 15 minutes and must be completed before you are allocated to your treatment group.

After these assessments, you will attend hospital for radiotherapy treatment planning, radiotherapy and chemotherapy treatment, periodic scans and other checks to monitor your progress, as described below.

Radiotherapy treatment planning

Before your radiotherapy treatment can start, your treatment will need to be planned. You will need to attend the radiotherapy department for a CT scan. This is in addition to the CT scan that you will have had to diagnose your disease. At this visit, the radiographers may make marks on your skin to make sure the same area is treated at each treatment session. After your planning scan, the physicist and your radiotherapy doctor will then carefully create your personal treatment plan using the CT scan images. This planning may take 2 weeks or more to complete. You will then receive an appointment for your first dose of radiotherapy.

Radiotherapy treatment

High energy X-ray beams are directed at the cancer from outside the body (this is known as external beam radiotherapy). The type of radiotherapy that you will receive is called Intensity Modulated Radiotherapy (IMRT). IMRT is able to target your tumour more precisely so that your tumour receives a very high dose of radiotherapy and normal healthy cells nearby receive a much lower dose. The radiotherapy treatment is given as a series of short, daily treatments using equipment similar to a large X-ray machine. The radiographers will help you to get into the right position on the radiotherapy couch. Once you are in the right position, the staff will leave you alone in the room so that they are not exposed to the radiation. They will watch you carefully either through a window or on a closed circuit television screen. The treatment is painless

Frood R, *et al. BMJ Open* 2025; 15:e109655. doi: 10.1136/bmjopen-2025-109655

and the machine will rotate around you without touching you over 10-20 minutes. Treatment will occur each weekday for 5½ weeks (28 weekday treatments in total).

Chemotherapy treatment

This is the use of anti-cancer drugs to destroy cancer cells. Chemotherapy used in this study is a combination of two drugs. One of the drugs is given by injection through a vein (intravenously) on the first day of radiotherapy. It is called **Mitomycin C**. The second drug is either a drug called **5FU** (which goes into your vein) or **Capecitabine** (which is given as tablets). 5FU is given on the 1st and 5th week of your radiotherapy for 4 days each time. Capecitabine tablets are taken twice a day each day of radiotherapy. Your hospital will tell you which of these two drugs they will use. Both drugs are equally as good, but different hospitals are used to different drugs so will use what they usually give.

It is important that you tell your doctor if you are taking, have recently taken or might take any other medicines. This is particularly important if you are taking any of the following:

- **allopurinol** (gout medicine)
- **warfarin** (blood-thinning medicine used to treat blood clots)
- **sorivudine** and **brivudine** (anti-viral medicines)
- **phenytoin** (medicine for seizures or tremors)
- **metronidazole** (antibiotic)
- **clozapine** (schizophrenia medicine)

9 How long does treatment go on for?

The chemoradiotherapy treatment is given over 5½ weeks (28 treatments). Occasionally it may go on for slightly longer than this if you experience side effects that mean treatment must be interrupted.

10 The effect of chemoradiotherapy on having children

Men:

Male patients will be required to use barrier contraception during chemoradiotherapy and until 6 months after finishing treatment. It is advised that men avoid having children during this time period unless this is through sperm stored before the treatment started. Whilst some men will be infertile after treatment, many will not.

Women:

It is vital to avoid pregnancy during the treatment and contraceptives should be used throughout. It is important to tell your clinical care team if you are pregnant or become pregnant during treatment, as this will affect your care.

It is likely you will be infertile after the treatment is finished. This means you will lose the ability to have children. However, if you think there is any chance you are pregnant in the future, please speak to your doctor.

Further information about the risks of infertility is provided in Section 11h.

11 What are the potential side effects of radiotherapy?

If you take part in the study, you must report any problems you have to your study nurse or doctor. There is also a contact number given at the end of this information sheet for you to phone if you become worried at any time.

With intensity modulated radiotherapy treatment (IMRT), there is very little normal tissue in the treatment area. This means that the risk of side effects is lower than non-IMRT techniques, but unfortunately you can still have side effects. As with any external beam radiotherapy, the side effects only affect the part of the body that the radiotherapy treatment is aimed at. Radiotherapy affects people in different ways, so it's difficult to predict exactly how you will react. Some people have only mild side effects, but for others the side effects are more severe. Knowing about the side effects may help you to prepare and manage any problems.

<u>Acute side effects of radiotherapy</u>

The side effects that occur during radiotherapy and for a few weeks after finishing radiotherapy treatment are known as 'acute' side effects. They usually get worse towards the end of treatment and then start to gradually get better a few weeks after treatment has ended, but may take some months to entirely settle back to normal.

You should be aware that the side effects listed below may be worse if you get one of the higher doses of radiotherapy

a) Effects on the skin

It's very likely that your skin will be sore in the area being treated, which is around the anus. There will possibly be other areas affected including the scrotum (the testicles and the skin sack they are held in) in men, the vulval area for women, and in the creases in your groin. This soreness usually starts about 2-3 weeks after treatment begins and clears up 3-6 weeks after it finishes. The skin may become blistered and sore and the area may be quite painful. You can be prescribed painkillers to help with this.

The hospital staff will keep an eye on the area and will advise you how to look after your skin. They will provide you with creams and dressings to use on the skin. It's important that you only use products they recommend.

b) Diarrhoea

During the course of treatment, you may notice changes in how your bowel works. You may experience diarrhoea, mucus or jelly-like discharge and pass more wind. These symptoms can sometimes be reduced by avoiding particular foods. Your doctor or a dietician at the hospital can give you advice about this. Most of the time the diarrhoea can be controlled by tablets, but your team will discuss other options if it becomes very troublesome.

c) Urinary problems

During radiotherapy passing urine may become painful or difficult and it may sometimes be difficult to hold your urine. If this happens, your medical team will treat any infection and may suggest pain killers or other tablets. Very occasionally a fine tube (called a catheter) is put into your bladder to drain the urine to relieve pain or symptoms.

Frood R, *et al. BMJ Open* 2025; 15:e109655. doi: 10.1136/bmjopen-2025-109655

d) Hair loss (from radiotherapy)

It's common to lose your pubic hair during radiotherapy. The hair should grow back after treatment finishes in most patients, although very occasionally the hair loss is permanent.

e) Extreme tiredness (fatigue)

Fatigue is a common side effect of radiotherapy for anal cancer. The tiredness usually starts around 3-4 weeks after treatment begins and may last for a number of weeks following treatment. Generally by around 8-12 weeks, most people feel their energy levels retuning, although this can be a longer period for some. It is important to pace yourself during this time. Your health care team will be able to give you advice on balancing rest with exercise/activities.

f) Vaginal irritation

The vagina can become irritated and inflamed during radiotherapy. It can be prone to infection, so please let staff know if there is an abnormal discharge or itch. Intercourse may be painful or cause bleeding and it may be advisable to not have intercourse until the skin has healed. Your nurse or radiographer will advise you on this and can offer practical advice to help minimise symptoms.

All these acute side effects usually decrease gradually once the treatment has ended, but it may take some months for skin changes to go back to normal.

<u>Late side effects of radiotherapy</u>

Side effects can also occur months and years after finishing radiotherapy. Some side effects can still be a problem years after the patient has finished treatment. These are known as 'chronic' side effects and are listed below.

You should be aware that the side effects may be increased if you receive one of the higher doses of radiotherapy.

g) Bowel problems

Some people find that the way their bowel works is permanently altered after radiotherapy and occasionally a patient has severe difficulties. Problems that can occur include: more regular bowel motions due to scarring; narrowing of the bowel passage causing difficulty in passing bowel motions; bleeding from the back passage due to new, fragile blood vessels formed as a result of radiotherapy; mucus or jelly-like discharge; an urgent need to go to pass a motion, but nothing comes out; or occasionally patients can lose control of bowel motions completely. These side effects can usually be managed although you may need to take medication. Your healthcare team will be able to advise you to minimise any bowel effects that you experience.

In very rare circumstances patients may require surgery to form a stoma (see end of Section 7). Any requirement would be discussed thoroughly with you and information provided to help inform decision making.

h) Infertility (loss of the ability to have children)

Pelvic radiotherapy is likely to cause infertility in women. This is because the ovaries usually stop producing eggs after radiotherapy and the uterus is less flexible and may not be able to carry a baby to full term. Pelvic radiotherapy may also cause infertility in men. If you are considering having a child in the

future, it is important to discuss your options with your doctor or nurse before treatment starts. Fertility treatments aimed at helping you to be able to have a child after your treatment has finished can be complicated. The options available to you will depend on your age, whether you already have children or a partner, and how soon your treatment needs to start. Your doctor can refer you to a fertility specialist for further discussion.

Men: Sperm can be stored for use in the future. It is important to talk to your doctor before the treatment starts. During radiotherapy you may still produce sperm, but these could be damaged, resulting in abnormalities in the child. It is therefore very important to use barrier contraception during treatment and for a number of months after treatment has finished.

i) Menopause

Women who are still having periods may find that treatment brings on an early menopause because of the reduction in hormones in the ovaries. This results in infertility and menopausal symptoms such as hot flushes and sweats, which may affect sexual activity. Your doctor or nurse can give you advice on managing menopausal symptoms. It usually takes about three months for the ovaries to stop producing eggs and during this time it is still possible to get pregnant. It is important to use contraception during radiotherapy if you are still of child-bearing age.

j) Vaginal dryness and tightening

Women may develop dryness and narrowing of the vagina after radiotherapy. This may make sexual intercourse difficult or uncomfortable. This can often be avoided with the use of “vaginal dilators” as soon as the soreness has resolved after treatment is finished. These are plastic devices that you put into the vagina twice a week to keep the vagina walls open and supple. You may also need to use a lubricating jelly during sex. Your doctor or specialist nurse can give you more information about this.

k) Bones

During radiotherapy, the radiation goes through the bones of your pelvis including your hips bones. As such, you will be at a slightly increased risk of pain in your pelvic bones, or very occasionally breaking these bones in future. If you are concerned please speak to your doctor about reducing the risk of these side effects.

l) Impotence

Men may become unable to have an erection (impotence) after treatment. It’s important to let your doctor know if this happens to you as there are ways this can be managed.

m) Urinary problems

There is a small risk of difficulties passing water or bleeding from the waterworks after finishing radiotherapy. Please let your doctor know if these are an issue because both can be treated.

n) Skin changes

The skin around the pelvis may look different after any blistering has subsided. It may be a slightly different colour than the surrounding skin, have visible blood vessels in it, or feel slightly thicker. It may be prone to bleeding. This does not usually cause a problem, but if you are worried about it please speak to your medical team. You may experience an itch

which can last for many months (it may be longer in some patients, although this is rare). Your medical team will advise on topical creams which you can apply to minimise the dryness and sooth the itching.

12 What are the potential side effects of chemotherapy?

a) Anaemia (low red blood cells)

Red blood cells carry oxygen round the body. If they are low, you have less oxygen and can be tired and breathless. The medical team will do weekly blood tests to pick this up and occasionally you may need a drip to give you extra red blood cells (blood transfusion).

b) Bruising and bleeding

Chemotherapy can reduce the number of platelets in your blood. Platelets are cells that help the blood to clot. Tell your doctor if you have any bruising or bleeding you can't explain. This includes nosebleeds, bleeding gums, blood spots or rashes on the skin. The medical team will do weekly blood tests to check platelets. Very occasionally you may require a drip to give you extra platelets (platelet transfusion).

c) Risk of infection

Chemotherapy can reduce the number of white blood cells in your blood. When the number of white blood cells is low, it's called neutropenia. White blood cells help your body fight infection. Neutropenia will make you more likely to get an infection.

Contact the hospital straight away on the contact number you've been given if you have a temperature over 37.5°C (99.5°F), you suddenly feel unwell (even with a normal temperature), or you have symptoms of an infection such as shivering, sweats, sore throat or pain on passing water.

The medical team will do weekly blood tests to check for neutropenia.

d) Nausea and vomiting

You may feel sick (nauseous) or be sick (vomiting). Medicines are given to counteract this and are generally very effective. If you continue to suffer nausea or vomiting consult your doctor or nurse, as there are many different anti-sickness medicines available.

e) Hair loss (from chemotherapy)

While the radiotherapy will cause pubic hair loss, some chemotherapy drugs can cause temporary hair loss in all areas of hair. However, this is uncommon with the drugs you will receive in this study.

f) Sore mouth

Chemotherapy drugs may make your mouth sore and cause mouth ulcers. Regular mouthwashes are important and your nurse will show you how to use these properly. Let your medical team know if your mouth continues to be sore as other treatments are available.

g) Severe skin reaction

Although rare, you may develop a severe skin reaction, such as skin rash, ulceration and blistering, which may involve ulcers of the mouth, nose, genitalia, hands, feet and eyes (red and swollen eyes). **Contact the hospital straight away should any of these symptoms occur.**

13 Very rare side effects of chemotherapy or radiotherapy

There is a very small risk of developing other side effects as a result of receiving chemotherapy. These can include abnormalities in your blood tests, heart problems or blood clots. Please let the medical team know if you have any concerns about any of these.

Giving radiotherapy or chemotherapy, whether given as part of standard treatment or in a study, carries a very small risk of severe side effects that can rarely result in the death of the patient. This risk should be discussed with your doctor and compared with the need for treatment and its possible benefits. Early treatment of side effects is important and you should inform the clinical team promptly if you experience side effects.

14 What if the treatment doesn't help?

Occasionally the treatment does not completely get rid of the cancer. If this happens your team will discuss the options with you. One option is to undergo a surgical operation, which would remove the anus and rectum and leave you with a permanent stoma (see end of Section 7).

15 How is my condition monitored?

You will be seen weekly throughout your chemoradiotherapy treatment and will be able to call for advice at any time.

Once your treatment is complete you will be under close follow up every few months with your cancer team for a total of 3-5 years.

16 What happens when treatment has finished?

After your treatment has finished, you will need to attend clinic for follow-up visits at 6 weeks after your treatment ends, then every 3 months for 2 years, every 6 months for 1 year, then once per year, until 3 years after the trial closes to recruitment. During these study visits, your doctor will examine you and record information about your progress.

You will have an MRI scan of your pelvis at 3 months and 6 months after the end of your treatment. Also, at 12, 24 and 36 months after the end of your treatment you will have a CT scan of your chest, abdomen and pelvis.

We would like you to complete a questionnaire about your quality of life (similar to the one you filled in at the start of the study) at the end of your treatment and again at 6 weeks, 6, 12, 24 and 36 months later. The questionnaire can be completed on-line, or on paper if you prefer.

Your doctor may wish to see you at other time points outside these study visits.

We will register your name, date of birth and, depending on which country you live in, your NHS number[1], CHI number[2], or H&C number[3] with NHS Digital or other registries. This is so we can continue to check your health status for the purposes of this study after you have stopped attending for your study visits.

[1] England and Wales, [2] Scotland, [3] Northern Ireland

17 What are the different scans that I will have?

CT scan

A computed tomography (CT scan) is a standard test used to create images of the inside of the body using X-rays. It is performed whilst you are lying down as you pass through a tunnel. Please ask your doctor if you wish to know more about this.

PET scan
A positron emission tomography (PET scan) involves a specialist scanner to create images of the inside of your body, which highlight areas of cancer. You have an injection of a very small amount of a radioactive drug (tracer) first. The amount of radiation is very small and does not make you feel unwell. It only stays in the body for a few hours. Please ask your doctor if you wish to know more about this.

MRI scan
A magnetic resonance imaging (MRI scan) uses strong magnetic fields to create images of the inside of your body. It is performed whilst you are lying down as you pass through a tunnel. It is a bit noisy and occasionally people can find it a little claustrophobic. Please ask your doctor if you wish to know more about this.

18 What risk is involved with scans I will have as part of the study?

CT and PET scans involve X-rays (radiation). For many patients, the number of scans and radiation exposure you will receive in this study are the same as if you were not in the study. For some patients, the number of scans and radiation exposure you will receive in this study may be more than you would usually receive. Each scan corresponds to about 5 years' exposure to the natural background radiation that we all receive throughout life.

19 What are the possible benefits of taking part?

We hope that taking part in this study will help you by giving you the best available treatment for your cancer. If you receive one of the higher doses of radiotherapy, it may reduce the chance of the cancer coming back.

20 What are the possible disadvantages and risks of taking part?

Although similar high doses of radiotherapy have been used in other trials done in other countries, the side effects that occur years after radiotherapy have not been studied. There is a risk that the side effects may be worse with the higher doses of radiotherapy, and require more treatment.

21 What if something goes wrong?

As with any cancer treatment, your doctors and nurses aim to ensure that any risks are kept to a minimum. The treatment is checked by a number of different people in every department before and during treatment, so the chance of it being delivered incorrectly is very small. Should this happen, or an unexpected problem arises during treatment your medical team will discuss the consequences and options with you.

The Trial Management Group and independent committees will monitor the study on an on-going basis so that if any of the treatments turn out to be much worse than the others, this will be detected as soon as possible and the study stopped. If you

experience problems, you must report these to your study nurse or doctor. Their contact numbers can be found at the end of this information sheet.

22 Will my taking part be kept confidential?

If you decide to take part in this study, the information collected about you will be handled strictly in accordance with the General Data Protection Regulation (GDPR) and 2018 Data Protection Act.

The study is being carried out in the United Kingdom and the University of Leeds is the Sponsor for this study. We will be using information from you and your medical records in order to undertake this study. The University of Leeds will act as the data controller for this study. As a publicly-funded organisation, we have to ensure that it is in the public interest when we use personally-identifiable information from people who have agreed to take part in research. This organisation will be responsible for looking after your information and using it properly. The University of Leeds will keep identifiable information about you for 15 years after the study has finished.

Your rights to access, change or move your information are limited, as we need to manage your information in specific ways in order for the research to be reliable and accurate. If you withdraw from the study, we will keep the information about you that we have already obtained. To safeguard your rights, we will use the minimum personally-identifiable information possible.

The University of Leeds will collect information about you for this research study from the hospital you are receiving treatment from. This information will include your name, date of birth and, depending on which country you live in, your NHS number, CHI number, or H&C number, as well as your postal or email address (if you consent to the quality of life study). This information is regarded as a special category of information. The reasons why we collect this are explained in Section 27 below.

If you have any concerns about the way your personal data is being processed or have a query about the information in this document, please contact the University of Leeds Data Protection Officer using any of the following details:

- Email: DPO@leeds.ac.uk;
- General postal address: University of Leeds, Leeds LS2 9JT, UK;
- Postal address for data protection issues: University of Leeds, Room 11.72, EC Stoner Building, Leeds, LS2 9JT;
- Telephone number: +44 (0)113 243 1751.

The data controller registration number provided by the Information Commissioner's Office is Z553814X.

If you are not satisfied with our response or believe we are processing your personal data in a way that is not lawful you can complain to the Information Commissioner’s Office (https://ico.org.uk/).

Please refer to Section 27 in Part 2 for further details about how your personal data will be handled.

Frood R, *et al. BMJ Open* 2025; 15:e109655. doi: 10.1136/bmjopen-2025-109655

This completes Part 1 of the Information Sheet. If the information in Part 1 has interested you and you are considering participation, please continue to read the additional information in Part 2 before making any decision.

Frood R, *et al. BMJ Open* 2025; 15:e109655. doi: 10.1136/bmjopen-2025-109655

Part 2

23 Additional research (optional)

We will be using this opportunity to study a lot of different aspects of anal cancer. We aim to use information from you to allow us to gain a better understanding of who is at risk of this type of cancer and who is most likely to develop significant side effects. Additional research includes:

1) With your permission, we would like to collect some of your stored biopsies (samples of tumour) from your diagnosis and if your disease relapses. These samples will be sent to a central laboratory and stored for future research within, or possibly outside, the UK. These studies may include genetic tests. The samples will be considered a gift. For this research, your samples will not have your name or address attached to them. They will be labelled with a code number that will enable the trials team to link the sample back to you, but you will not be identified or contacted.

2) We would also like to collect scans taken as part of the study to look at other aspects of anal cancer. These scans will be sent to a central department and held in a secure NHS server. They will be considered a gift. These scans will be used for future research within or possibly outside the UK. In this research, your scans will not have your name / address or details attached to them but the trials team will be able to link them back to you.

It is important to note that collection of these samples and scans and participation in this additional research is optional. Even if you choose to enter the main part of the study, you may choose not to provide these samples. This will not affect your participation in this study, your treatment, or your relationship with your doctor.

24 What if relevant new information becomes available?

Sometimes during the course of a study, new information becomes available. You will be informed if new information comes to light regarding any of the study treatments or your cancer that could have a bearing on the treatment of your cancer. We will notify you of any changes in the way the study is run at the earliest opportunity and discuss with you whether you want to continue in the study. If you decide not to continue, your cancer team will continue with your care as appropriate, and discuss other treatment options with you. If you decide to continue you may be asked to sign an updated consent form. Occasionally on receiving new information, your cancer team may consider it to be in your best interest to withdraw you from further study treatment.

25 What will happen if I don’t want to carry on with the study?

You may withdraw from the study at any time. You will not need to give a reason, however, we would like to know the reason if you are willing to say. Before deciding to stop, you should talk to your study doctor or nurse. They can advise you and may be able to deal with any concerns you may have. Information about your participation will remain confidential.

Frood R, *et al. BMJ Open* 2025; 15:e109655. doi: 10.1136/bmjopen-2025-109655

Even if you do not wish to continue with study treatment, we would still like you to attend for trial visits and assessments and to continue to collect information about you and how you are feeling, unless you request otherwise.

If you do withdraw consent for study treatment and/or further data collection, your data collected up until that point will remain on file and will be included in the final study analysis.

26 Harm and complaints

Every care will be taken in the course of this clinical study. However, in the unlikely event that you are injured as a result of the managing organisation (University of Leeds), compensation may be available but you may have to pay your related legal costs. Your hospital where you receive your treatment has a duty of care to you whether or not you agree to take part in the study and the University of Leeds accepts no liability for negligence on the part of your hospital's employees. If you wish to complain about any aspect of the way you have been treated please contact your research doctor in the first instance.

Any claims will be subject to UK law and must be brought in the UK.

Regardless of this, if you wish to complain, or have any concerns about any aspect of the way you have been approached or treated during the course of this study, the normal National Health Service complaints services are available to you. These are unique to your local NHS Trust. Your study nurse/doctor can give you their information.

If you have private medical insurance, you should contact your insurer, who will let you know whether participation in this study will affect your policy.

27 Will my taking part be kept confidential?

If you decide to take part in ACT5, the information collected about you will be handled in accordance with the General Data Protection Regulation (GDPR) and 2018 Data Protection Act. The information needed for study purposes will be collected on paper forms and sent (usually using standard Royal Mail post, but in some cases by fax, email or secure electronic transfer), from the hospital to the University of Leeds Clinical Trials Research Unit (CTRU). Paper records will be stored in locked filing cabinets. You will be allocated a study number, which will be used along with your date of birth and initials to identify you on each paper form. Your full name and signature will be included on your consent form, and a copy of this will be sent to the CTRU by fax, post or secure electronic transfer. In addition, your full name and postal address or email address will be sent to the CTRU by fax, post or secure electronic transfer to allow the CTRU to issue Quality of Life questionnaires online or by post. Every effort will be made to ensure that any further information about you that leaves the hospital will have your name and address removed so that you cannot be recognised from it. This information will usually be removed by a member of the study team at your hospital, but if this does not happen, it will be removed by the CTRU upon receipt.

If you agree to use the online system (called REDCap) to complete your Quality of Life questionnaires, the CTRU will email a you a

Frood R, et al. BMJ Open 2025; 15:e109655. doi: 10.1136/bmjopen-2025-109655

link to the online questionnaire when it is due to be completed.

When completing the online questionnaires, we recommend using a trusted personal computer/device and avoiding using shared public computers such as the ones in public libraries, as they can be less secure and more open to viruses.

Your GP, and the other doctors involved in your clinical care, will be kept informed of your participation in this study, but otherwise all information about you and your treatment will remain confidential.

We will register your name, date of birth and, depending on which country you live in, your NHS number, CHI number, or H&C number with NHS Digital or other registries. This is so we can continue to check your health status for the purposes of this study after you have stopped attending for your study visits.

Your data will be entered onto a secure database held at the CTRU in accordance with the 2018 Data Protection Act.

Your anonymised data may be passed to other organisations (possibly in other countries where the data protection standards and laws are different to the UK) to monitor the safety of the treatment(s) that you are receiving; this data will have your name removed.

Some of the scans from selected participants may be sent to other hospitals to be looked at by other doctors. This is to ensure that results / reports are consistent across hospitals. These will be sent via standard hospital processes (such as Royal Mail or courier). This data will be anonymised and your name removed.

Your healthcare records may be looked at by authorised individuals from the research team, the University of Leeds (the study Sponsor), or the regulatory authorities, to check that the study is being carried out correctly.

The information collected about you may be shared with other research teams to answer new research questions in future. These researchers may be in this or other organisations. These organisations may be universities, NHS organisations or companies involved in health and care research in this country or abroad. Your information will only be used by organisations and researchers to conduct research in accordance with the UK Policy Framework for Health and Social Care Research. Wherever possible, information will be anonymised. Where this information could identify you, the information will be held securely with strict arrangements about who can access the information. The information will only be used for the purpose of health and care research, or to contact you about future opportunities to participate in research. It will not be used to make decisions about future services available to you, such as insurance.

Where there is a risk that you can be identified your data will only be used in research that has been independently reviewed by an ethics committee.

When the study is finished, the results will be published in a medical journal, but no individual participants will be identified.

In line with Good Clinical Practice guidelines, at the end of the study, your data will be securely archived for a minimum of 15 years. Arrangements for confidential destruction will then be made.

28 What will happen if I lose mental capacity during the study period?

This is expected to be a very rare occurrence. It could happen to any patient whether or not they take part in this study. If this did occur, no further study treatment would be given and data collection for the study would stop. Data collected up until this point will remain on file and will be included in the study analysis.

29 What happens when the research study stops?

The study will stop three years after the last patient is randomised. After this time the frequency at which you are seen by your doctor may vary slightly depending on your hospital's policy, but you are likely to be seen either 6 monthly or yearly until it is 5 years after your treatment ended.

Five years after your treatment you are usually discharged from follow up, but can always contact the team if you have any concerns following discharge.

30 What will happen to the results of the research study?

When the study is complete, the results will be made openly available to the scientific community and will be published in a medical journal. Your personal information will be protected and kept confidential at all times and no individual participants will be named in any published material. If you would like to obtain a copy of the published results, please ask your doctor. We will prepare a summary which will be available to patients when submitting the results to a journal to make it easier to understand.

31 Who is organising and funding the research?

This study is funded by the Cancer Research UK (CRUK) Clinical Trials Advisory and Awards Committee (CTAAC). The study is being organised by the University of Leeds, through the Clinical Trials and Research Unit (CTRU), who will collect and analyse your data. The study is also sponsored by the University of Leeds.

32 Who has reviewed the study and who will monitor it?

The study was reviewed and approved by the National Cancer Research Institute Colorectal Clinical Studies Group, the NHS Health Research Authority and the Research and Development (R&D) department at your hospital. It has also been reviewed by an independent NHS Research Ethics Committee.

The study is supervised by two independent committees called the Data Monitoring and Ethics Committee (DMEC) and the Trial Steering Committee (TSC). These committees include experienced cancer doctors, statisticians and a patient representative.

33 Further information

If you have any further questions about your cancer or clinical trials, please discuss them with your doctor. You may also find it helpful to contact Macmillan Cancer Support, an independent cancer information charity (free phone: 0808 808 0000; address: 89 Albert

Frood R, *et al. BMJ Open* 2025; 15:e109655. doi: 10.1136/bmjopen-2025-109655

Embankment, London, SE1 7UQ; web site www.macmillan.org.uk), or CancerHelp.

CancerHelp is an information service Cancer Research UK (Tel: 020 7061 8355; website www.cancerhelp.org.uk). If you would like further information about clinical research, the UK Clinical Research Collaboration (a partnership of organisations working together on clinical research in the UK) have published a booklet entitled 'Understanding Clinical Trials'. Contact UKCRC: Tel: 0207 670 5452; website www.ukcrc.org

An on-line tool that explains some of the terminology used in this information sheet is available on the CancerHelp website http://www.cancerresearchuk.org/cancer-help/utilities/glossary/.

Thank you very much for taking an interest in this research

Frood R, *et al. BMJ Open* 2025; 15:e109655. doi: 10.1136/bmjopen-2025-109655

Glossary of terms

Short term	Long term	What it means
Anaemia		A low level of red blood cells and/or haemoglobin in your blood. Haemoglobin carries oxygen around the body. It can make you feel very tired, weak or breathless.
Chemoradiotherapy		A combination of chemotherapy and radiotherapy treatment. Chemotherapy is given on the same days as radiotherapy
CT scan	Computer Tomography scan	Using X-Rays to create a 2D picture of inside the body. The image produced is very detailed.
CTRU	Clinical Trials Research Unit	This is the organisation coordinating the study. It is part of the University of Leeds.
IMRT	Intensity Modulated Radiotherapy	This is a way of giving radiotherapy to a tumour in a way that targets the treatment very accurately, and where the normal healthy cells nearby receive a much lower dose.
MRI scan	Magnetic Resonance Imaging scan	Using a magnetic field to create a picture of inside the body. This is very useful for telling the difference between muscle, soft tissue and bone.
Neutropenia		A low level of neutrophils, a type of white blood cell. Neutrophils help the body fight infection. People who have neutropenia have a higher risk of getting serious infections.
PET scan	Positron Emission Tomography scan	Using a scan to detect radiation given off by a small amount of a radioactive drug (tracer) that is injected into you before the scan. It shows how the body tissues are working and what they look like. It is often combined with a CT scan to create more detailed pictures.
Randomisation		Using a computer programme to decide which treatment a patient will. In this way, a fair comparison between the treatments can be made
Stoma		An opening on your abdomen or tummy that allows faeces to bypass your back passage and pass straight into a bag.

Frood R, *et al. BMJ Open* 2025; 15:e109655. doi: 10.1136/bmjopen-2025-109655

Summary of your participation in ACT5

	Routine Procedures	Extra Procedures
**Eligibility and Consent**	• Physical exam<br>• Medical history<br>• Diagnostic biopsy<br>• Blood tests<br>• Pregnancy test<br>• CT scan<br>• MRI scan	• PET CT scan may be done. If done, this may be routine or extra, depending on your hospital's local policy.
**Randomisation**	**14 days before treatment:**<br>• Heart test (ECG)<br>**10 days before treatment:**<br>• Blood tests	• Questionnaire
	You will be randomised to receive standard-dose radiotherapy plus chemotherapy, or one of two higher doses of radiotherapy plus chemotherapy	
**Radiotherapy planning**	• CT scan to plan your radiotherapy treatment	
**Radiotherapy and chemotherapy 5½ weeks (28 weekday treatments)**	**Each week:**<br>• Blood tests<br>• Assessment of side effects	
**End of Treatment**	• Blood tests<br>• Assessment of side effects	• Questionnaire
**6 weeks after end of treatment**	• Assessment of side effects	• Questionnaire<br>• Physical exam
**Follow Up: Every 3 months for 2 years, then every 6 months for at least 1 year**	• Physical exam<br>• Assessment of side effects at 3 and 6 months after the end of treatment<br>• MRI scan at 3 and 6 months after the end of treatment<br>• CT scan at 12, 24 and 36 months after the end of treatment	• Questionnaire at 6, 12, 24 and 36 months after the end of treatment

Frood R, *et al. BMJ Open* 2025; 15:e109655. doi: 10.1136/bmjopen-2025-109655

(Reprint this form on headed paper)

Participant ID:	Initials:
Date of Birth:	NHS/CHI/H&C Number:
ISRCTN: 88455282	Principal Investigator:

ACT5 (Anal Cancer Trial 5)

CONSENT FORM

1. I confirm that I have read and understand the information sheet for the above study and have had the opportunity to ask questions.

2. I understand that my participation is voluntary and that I am free to withdraw at any time, without giving any reason and without my medical care or legal rights being affected. I understand that even if I withdraw from the above study, the data collected from me will be used in analysing the results of the study and in some cases, further information about any unwanted effects of my treatment may need to be collected by the study team.

3. I understand that my healthcare records may be looked at by authorised individuals from the study team, regulatory bodies or Sponsor in order to check that the study is being carried out correctly.

4. I understand that the information held and maintained by NHS Digital and other central UK registries may be used to help contact me or provide information about my health status. I give permission for my details (which will include my name, date of birth and NHS number, CHI number, or H&C number) to be submitted to these bodies so that information about my health status may be obtained by the CTRU if necessary.

5. I agree to a copy of this Consent Form being sent to the CTRU.

6. I agree that my general practitioner (GP), or any other doctor treating me, will be notified of my participation in this study.

7. I agree to allow any information or results arising from this study to be used for healthcare and/or further medical research upon the understanding that my identity will remain anonymous.

8. I understand that if during this study my clinical care team determine that I have lost my ability to make my own decisions, no further study intervention will be given. I agree that information collected up until this point will remain on file and will be included in the analysis.

9. I agree to take part in this study.

The following points are OPTIONAL. Even if you agree to take part in this study, you do not have to agree to the questions in this section.

Please tick ✓

	Yes	No
10. I agree to take part in the Quality of Life study and understand that my email address or postal address will be passed to the CTRU for the purpose of issuing the study Quality of Life questionnaires (either online, or via the post)	☐	☐
11. I give permission for surplus samples from my cancer to be retrieved from my hospital's pathology laboratory and sent to a central laboratory, to be stored and used for future cancer research that receives ethical approval. I understand that my name will not be stored on the samples, but they will be labelled with a unique reference number that will allow the sample to be linked back to me in future, for research purposes. I understand that my tissue samples are a 'gift' and may be shared on a collaborative basis with researchers in the UK and potentially abroad, including outside the European Economic Area. I understand that my tissue samples may be used for genetic research.	☐	☐
12. I give permission for any scans taken as part of the study to be sent to a central department and held in a secure NHS server. They will be considered a gift. These scans will be used for future research that receives ethical approval within or possibly outside the UK. In this research, your scans will not have your name / address or details attached to them but the trials team will be able to link them back to you.	☐	☐

Frood R, *et al. BMJ Open* 2025; 15:e109655. doi: 10.1136/bmjopen-2025-109655

Patient:
Signature………………………………………………………………………………

Name (block capitals)……………………………………………………………………

Date………………………………………………………………………………………

Investigator:
I have explained the study to the above named patient and he/she has indicated his/her willingness to participate.

Signature………………………………………………………………………………

Name (block capitals)……………………………………………………………………

Date………………………………………………………………………………………

(If used)Translator:

Signature………………………………………………………………………………

Name (block capitals)……………………………………………………………………

Date………………………………………………………………………………………

(1 copy for patient; 1 copy sent to CTRU; 1 copy for patient notes, original stored in Investigator Site File)

Frood R, *et al. BMJ Open* 2025; 15:e109655. doi: 10.1136/bmjopen-2025-109655
