# Supplementary material for: Personalising anal cancer radiotherapy dose (PLATO): protocol for a multicentre integrated platform trial
Source: BMJ Open. 2025 Nov 9;15(11):e109655. doi: 10.1136/bmjopen-2025-109655 (PMC12658545; doi:10.1136/bmjopen-2025-109655)
Supplement: Supplementary data [file bmjopen-15-11-s001.pdf]

Translational research sample size

Analysis of p16, HPV and loco-regional failure

The sample size calculations in ACT4 are inflated to allow for a pre-planned analysis of p16/HPV. With 108 in the dose de-escalation arm of ACT4, we will have around 88% power to the test the dose de-escalation strategy and demonstrate efficacy rates of up to 90%. Assuming only 90% of these patients will have samples suitable for analysis, and 85% will be p16+, we will have 80% power to demonstrate efficacy rates of up to 90% within the p16+ve sub-group. With respect to the ACT5 question around dose escalation, 140 patients in the pilot and phase II stages of ACT5 give approximately 94 patients in the dose escalated arms. Assuming a relapse rate of 30%, where 85% are p16+ve (n=80) and 15% p16-ve (n=14), there would be 94% power to detect a HR of 4.5 between the p16 +ve/-ve groups. With a lower relapse rate of 20% in the dose escalated arms (as targeted within ACT5), the power would reduce to 85%. With 90% of samples suitable for analysis (so 126 patients with samples at the end of phase II, 84 in the dose escalated arm) and assuming a relapse rate of 30%, 85% p16+ve (n=71), and 15% p16-ve (n=13), there would be 90% power to detect a HR of 4.5 between the p16 +ve/-ve groups. If we assumed a relapse rate of 20% in the dose escalated arms (which is what were are targeting in ACT5), the power would reduce to 78%. The power to demonstrate a relationship between TIL score and relapse within p16 +ve patients is about 80% for a relapse rate of 30% and just over 50% for a relapse rate of 20%.

Analysis of cfDNA

Serial data on CTCs and cfDNA (presence/absence and enumeration/ quantification) will be analysed for the cohort of patients in ACT5 (phase II/III) with an estimated 25% risk of recurrence at 1 year. Performance characteristics (including ROC) will be determined for these data (CTC and cfDNA) in relation to complete response and 1 year disease free survival. For such longitudinal monitoring power calculation methodology is less clearly developed, but with 6 marker values throughout the follow-up period on 80 patients we will only be powered to detect relatively large effects.

Analysis of changes in gut microbiome

The faecal microbiome in patients undergoing radical treatment for anal cancer has not previously been investigated and as such all analyses and comparisons are exploratory. Our primary outcome measure is the feasibility of collecting and analysing the faecal microbiome in patients enrolled in ACT5 in >65% cases, i.e. obtaining paired, evaluable samples in at least 65% of patients. If we recruit at least 55 patients into the faecal microbiome sub-study, evaluable paired samples in 42 of these

will give us 80% power (at the 1-sided 5% significance level) to achieve this, assuming a true paired, evaluable rate of 80%.

Target volume definition

Gross Tumour Volume (GTV) – ACT3, ACT4 and ACT 5 patients

The GTV will be determined by the treating clinician using the planning CT, and information from clinical data and diagnostic imaging.

- GTV_A = Site of primary tumour and scar (ACT3)
- GTV_A = Includes the gross primary anal tumour volume (ACT4 and ACT5)
- GTV_N = Includes all involved nodes ≤3cm (ACT5)
- GTV_N3 = Includes all involved nodes >3cm (ACT5)

Clinical Target Volume (CTV) – ACT3, ACT4 and ACT5 patients

All the expansions are in 3 dimensions unless stated otherwise.

- CTV_A = GTV_A + 10mm (ACT3)
- CTV_A = GTV_A + 10mm (ACT4)
- CTV_A = GTV_A +15mm (ACT5)

This is manually enlarged to ensure coverage of the anal canal, anal verge, internal and external anal sphincters. In the absence of bony involvement this is then edited to exclude bone.

- CTV_N = GTV_N + 5mm. This is also edited off bone and muscles in the absence of bony involvement (ACT5)
- CTV_E = Elective nodal regions (ACT4 and ACT5).

Planning Target Volume (PTV) – ACT3, ACT4 and ACT5 patients

All the expansions are in 3 dimensions unless stated otherwise.

ACT3 patients:-

- PTV_A= CTV_A + 10mm

ACT4 patients:-

- PTV_A= CTV_A + 10mm

- PTV_E = CTV_E + 5mm

ACT5 patients:-

- PTV_A= CTV_A + 10mm
- PTV_N = CTV_N + 5mm
- PTV_E = CTV_E + 5mm
