# Supplementary material for: Personalising anal cancer radiotherapy dose (PLATO): protocol for a multicentre integrated platform trial
Source: BMJ Open. 2025 Nov 9;15(11):e109655. doi: 10.1136/bmjopen-2025-109655 (PMC12658545; doi:10.1136/bmjopen-2025-109655)
Supplement: Supplementary data [file bmjopen-15-11-s002.pdf]

ACT3 treatment summary

Patients with anal tumour margins >1mm (deep and lateral) will undergo observation; patients with anal tumour margins ≤1mm (close or involved) will receive lower-dose CRT.

ACT3 observation arm (if margin >1mm)

Patients in this arm will be under observation as per local practice but must attend trial follow-up assessments.

ACT3 – Radiotherapy plus MMC capecitabine (if margin ≤1mm)

Treatment	Week																						
	1					2					3					4					5		
	Days 1-5					Days 8-12					Days 15-19					Days 22-26					Days 29-31		
Mitomycin C 12mg/m^2 iv	•																						
Capecitabine 825mg/m^2 orally bd Mon-Fri*	• •	• •	• •	• •	• •	• •	• •	• •	• •	• •	• •	• •	• •	• •	• •	• •	• •	• •	• •	• •	• •	• •	• •
Radiotherapy CTV 41.4Gy 23F	•	•	•	•	•	•	•	•	•	•	•	•	•	•	•	•	•	•	•	•	•	•	•

*recommended, but treatment may start on any day of the week.

ACT4 treatment summary

Patients will be randomised on a 1:2 basis (standard-dose: reduced-dose) to receive either standard-dose IMRT in combination with chemotherapy or reduced-dose IMRT in combination with chemotherapy.

ACT4 - Standard-dose IMRT plus MMC capecitabine

Treatment	Week																											
	1					2					3					4					5					6		
	Days 1-5					Days 8-12					Days 15-19					Days 22-26					Days 29-33					Days 36-38		
Mitomycin C 12mg/m^2 iv	•																											
Capecitabine 825mg/m^2 orally bd Mon-Fri*	• •	• •	• •	• •	• •	• •	• •	• •	• •	• •	• •	• •	• •	• •	• •	• •	• •	• •	• •	• •	• •	• •	• •	• •	• •	• •	• •	• •
Radiotherapy GTV 50.4Gy CTV 40Gy 28F	•	•	•	•	•	•	•	•	•	•	•	•	•	•	•	•	•	•	•	•	•	•	•	•	•	•	•	•

*recommended, but treatment may start on any day of the week.

ACT4 - Reduced-dose IMRT plus MMC capecitabine

Treatment	Week																						
	1					2					3					4					5		
	Days 1-5					Days 8-12					Days 15-19					Days 22-26					Days 29-31		
Mitomycin C 12mg/m^2 iv	●																						
Capecitabine 825mg/m^2 orally bd Mon-Fri*	●<br>●	●<br>●	●<br>●	●<br>●	●<br>●	●<br>●	●<br>●	●<br>●	●<br>●	●<br>●	●<br>●	●<br>●	●<br>●	●<br>●	●<br>●	●<br>●	●<br>●	●<br>●	●<br>●	●<br>●	●<br>●	●<br>●	●<br>●
Radiotherapy GTV 41.4Gy CTV 34.5Gy 23F	●	●	●	●	●	●	●	●	●	●	●	●	●	●	●	●	●	●	●	●	●	●	●

*recommended, but treatment may start on any day of the week.

ACT5 treatment summary

Patients will receive either standard-dose IMRT or one of two increased-dose experimental arms of IMRT with SIB, both in combination with chemotherapy. ACT5 will use Mitomycin C combined with either Capecitabine or 5 Fluorouracil (5FU).

ACT5 - Standard-dose IMRT plus MMC capecitabine

Treatment	Week																											
	1					2					3					4					5					6		
	Days 1-5					Days 8-12					Days 15-19					Days 22-26					Days 29-33					Days 36-38		
Mitomycin C 12mg/m^2 iv	●																											
Capecitabine 825mg/m^2 orally bd Mon-Fri*	●<br>●	●<br>●	●<br>●	●<br>●	●<br>●	●<br>●	●<br>●	●<br>●	●<br>●	●<br>●	●<br>●	●<br>●	●<br>●	●<br>●	●<br>●	●<br>●	●<br>●	●<br>●	●<br>●	●<br>●	●<br>●	●<br>●	●<br>●	●<br>●	●<br>●	●<br>●	●<br>●	●<br>●
Radiotherapy GTV 53.2Gy CTV 40Gy 28F	●	●	●	●	●	●	●	●	●	●	●	●	●	●	●	●	●	●	●	●	●	●	●	●	●	●	●	●

*recommended, but treatment may start on any day of the week.

ACT5 – SIB1 IMRT plus MMC capecitabine

Treatment	Week																											
	1					2					3					4					5					6		
	Days 1-5					Days 8-12					Days 15-19					Days 22-26					Days 29-33					Days 36-38		
Mitomycin C 12mg/m^2 iv	●																											
Capecitabine 825mg/m^2 orally bd Mon-Fri*	●<br>●	●<br>●	●<br>●	●<br>●	●<br>●	●<br>●	●<br>●	●<br>●	●<br>●	●<br>●	●<br>●	●<br>●	●<br>●	●<br>●	●<br>●	●<br>●	●<br>●	●<br>●	●<br>●	●<br>●	●<br>●	●<br>●	●<br>●	●<br>●	●<br>●	●<br>●	●<br>●	●<br>●

Radiotherapy GTV 58.8Gy CTV 40Gy 28F	•	•	•	•	•	•	•	•	•	•	•	•	•	•	•	•	•	•	•	•	•	•	•	•	•	•	•	•

*recommended, but treatment may start on any day of the week.

ACT5 – SIB2 IMRT plus MMC capecitabine

Treatment	Week																											
	1					2					3					4					5					6		
	Days 1-5					Days 8-12					Days 15-19					Days 22-26					Days 29-33					Days 36-38		
Mitomycin C 12mg/m^2 iv	•																											
Capecitabine 825mg/m^2 orally bd Mon-Fri*	• •	• •	• •	• •	• •	• •	• •	• •	• •	• •	• •	• •	• •	• •	• •	• •	• •	• •	• •	• •	• •	• •	• •	• •	• •	• •	• •	• •
Radiotherapy GTV 61.6Gy CTV 40Gy 28F	•	•	•	•	•	•	•	•	•	•	•	•	•	•	•	•	•	•	•	•	•	•	•	•	•	•	•	•

*recommended, but treatment may start on any day of the week.

ACT5 - Standard-dose IMRT plus MMC 5FU

Treatment	Week																											
	1					2					3					4					5					6		
	Days 1-5					Days 8-12					Days 15-19					Days 22-26					Days 29-33					Days 36-38		
Mitomycin C 12mg/m^2 iv	•																											
5FU 1000mg/m^2/24h iv	•	•	•	•																	•	•	•	•				
Radiotherapy GTV 53.2Gy CTV 40Gy 28F Mon-Fri*	•	•	•	•	•	•	•	•	•	•	•	•	•	•	•	•	•	•	•	•	•	•	•	•	•	•	•	•

ACT5 - SIB1 IMRT plus MMC 5FU

Treatment	Week																											
	1					2					3					4					5					6		
	Days 1-5					Days 8-12					Days 15-19					Days 22-26					Days 29-33					Days 36-38		
Mitomycin C 12mg/m^2 iv	•																											
5FU 1000mg/m^2/24h iv	•	•	•	•																	•	•	•	•				

Radiotherapy GTV 58.8Gy CTV 40Gy 28F Mon-Fri*	•	•	•	•	•	•	•	•	•	•	•	•	•	•	•	•	•	•	•	•	•	•	•	•	•	•	•	•

ACT5 – SIB2 IMRT plus MMC 5FU

Treatment	Week																											
	1					2					3					4					5					6		
	Days 1-5					Days 8-12					Days 15-19					Days 22-26					Days 29-33					Days 36-38		
Mitomycin C 12mg/m² iv	•																											
5FU 1000mg/m²/24h iv	•	•	•	•																	•	•	•	•				
Radiotherapy GTV 61.6Gy CTV 40Gy 28F Mon-Fri*	•	•	•	•	•	•	•	•	•	•	•	•	•	•	•	•	•	•	•	•	•	•	•	•	•	•	•	•

*recommended, but radiotherapy may start on any day of the week. Regardless of when radiotherapy starts, MMC and 5FU should start on the first Monday or Tuesday of radiotherapy.

ACT3 Assessment schedule

ACT3	Baseline			Treatment					Follow-up from end of treatment (or date of registration for observational arm)														
	Eligibility assessments	Pre reg	Pre tx	Week 1	Week 2	Week 3	Week 4	Week 5	6 weeks	3 months	6 months	9 months	12 months	15 months	18 months	21 months	24 months	30 months	36 months	48 months	60 months	Then annually[5]	Relapse
Medical history	●																						
ECOG PS, Vital Signs, ht/wt	●[1]																						
Physical exam	●								●*	●	●	●	●	●	●	●	●	●	●	●	●		
Tumour biopsy	●																						
Pregnancy test	●*[1]																						
Full Blood Count:	●[1]		●*[4]	●*	●*	●*	●*	●*															
U&E	●[1]		●*[4]	●*	●*	●*	●*	●*															
LFTs	●[1]		●*[4]	●*	●*	●*	●*	●*															
HIV	●*[2]	●**[2]																					
CD4 count if HIV +ve	●*[2]	●**[2]																					
ECG	●*[1]																						
MRI scan	●[3]												*						*				
Informed consent		●																					
Registration		●																					
QoL		●						●*	● CTRU to administer		● CTRU to administer		● CTRU to administer				● CTRU to administer		● CTRU to administer				
Radiotherapy planning scan		●*																					
Data collection	●	●	●*	●*	●*	●*	●*	●*		●	●	●	●	●	●	●	●	●	●	●	●	●	
CTCAE acute toxicity monitoring				●*	●*	●*	●*	●*															
SAR monitoring and reporting				Monitor during weeks of treatment*																			

Translational research samples (explicit consent required)

Tumour Biopsy	●[6]																						●[6, 7]

*RT arm only
** observation arm only
[1]within 14 days prior to registration
[2]within 28 days prior to registration
[3]within 63 days prior to registration
[4]within 10 days prior to start of treatment
[5]until 3 years after the last participant has completed treatment or death
[6]if consented to retrieval of stored material for future cancer research
[7]and where clinically indicated

ACT4 Assessment schedule

ACT4	Baseline			Treatment					
	Eligibility assessments	Pre rand	Pre tx	Week 1	Week 2	Week 3	Week 4	Week 5	Week 6
Medical history	•								
ECOG PS, Vital Signs, ht/wt	•[1]								
Physical exam	•								
Tumour Biopsy	•								
Pregnancy test	•[1]								
Full Blood Count	•[1]		•[5]	•	•	•	•	•	•*
U&E	•[1]		•[5]	•	•	•	•	•	•*
LFTs	•[1]		•[5]	•	•	•	•	•	•*
HIV	•[2]								
CD4 count if HIV +ve	•[2]								
ECG	•[1]								
CT scan	•[3]								
MRI scan	•[4]								
PET scan	•[4] Strongly recommended								
Informed consent		•							
Randomisation		•							
QoL		•						• 41.4Gy arm only	• 50.4Gy arm only
Radiotherapy planning scan		•							
Data collection	•	•	•	•	•	•	•	•	•*
CTCAE acute toxicity monitoring				•	•	•	•	•	•*
SAR monitoring and reporting				Monitor during weeks of treatment					
Response assessment									

ACT4	Follow-up from end of treatment														
	6 weeks	3 months	6 months	9 months	12 months	15 months	18 months	21 months	24 months	30 months	36 months	48 months	60 months	then annually[6]	Relapse
Medical history															
ECOG PS, Vital Signs, ht/wt															
Physical exam	•	•	•	•	•	•	•	•	•	•	•	•	•		
Tumour Biopsy															
Pregnancy test															
Full Blood Count															
U&E															
LFTs															
HIV															
CD4 count if HIV +ve															
ECG															
CT scan					•				•		•				
MRI scan		•	•												
PET scan															
Informed consent															
Randomisation															
QoL	• CTRU to administer		• CTRU to administer		• CTRU to administer				• CTRU to administer		• CTRU to administer				
Radiotherapy planning scan															
Data collection		•	•	•	•	•	•	•	•	•	•	•	•	•	
CTCAE acute toxicity monitoring															
SAR monitoring and reporting															
Response assessment		•	•												

Translational research samples (explicit consent required)

	Eligibility assessments	Pre rand	Pre tx	Week 1	Week 2	Week 3	Week 4	Week 5	Week 6	6 weeks	3 months	6 months	9 months	12 months	15 months	18 months	21 months	24 months	30 months	36 months	48 months	60 months	then annually	Relapse
Tumour Biopsy	•[7]																							•[7,8]

*50.4Gy arm only
[1]within 14 days prior to randomisation.
[2]within 28 days prior to randomisation
[3]within 63 days prior to randomisation. If the CT scan falls outside this timeframe but a PET CT has been carried out, there is no requirement to repeat the CT scan as long as the PET CT is a whole body scan.
[4]as a minimum, 1 of the MRI or PET scan (if done) for pelvic staging must be within 42 days before randomisation
[5]within 10 days prior to start of treatment
[6]until 3 years after the last participant has completed treatment or death
[7]if consented to retrieval of stored material for future cancer research
[8]and where clinically indicated

ACT5 Assessment schedule

ACT5	Baseline			Treatment						Follow-up from end of treatment														
	Eligibility assessments	Pre rand	Pre tx	Week 1	Week 2	Week 3	Week 4	Week 5	Week 6	6 weeks	3 months	6 months	9 months	12 months	15 months	18 months	21 months	24 months	30 months	36 months	48 months	60 months	then annually [6]	Relapse
Medical history	•																							
ECOG PS, Vital Signs, ht/wt	• [1]																							
Physical exam	•									•	•	•	•	•	•	•	•	•	•	•	•	•		
Tumour Biopsy	•																							
Pregnancy test	• [1]																							
Full Blood Count	• [1]		• [5]	•	•	•	•	•	•															
U&E	• [1]		• [5]	•	•	•	•	•	•															
LFTs	• [1]		• [5]	•	•	•	•	•	•															
HIV	• [2]																							
CD4 count if HIV +ve	• [2]																							
ECG	• [1]																							
CT scan	• [3]													•				•		•				
MRI scan	• [4]										•	•												
PET scan	• [4] Strongly recommended																							
Informed consent		•																						
Randomisation		•																						
QoL		•							•	• CTRU to administer		• CTRU to administer		• CTRU to administer				• CTRU to administer		• CTRU to administer				
Radiotherapy planning scan		•																						
Data collection	•	•	•	•	•	•	•	•	•	•	•	•	•	•	•	•	•	•	•	•	•	•	•	
CTCAE acute toxicity monitoring				•	•	•	•	•	•	•	•	•												
SAR monitoring and reporting				Monitor during weeks of treatment and until 6 months post end of treatment																				
Response assessment											•	•												

Translational research samples (explicit consent required)

ACT5	Eligibility assessments	Pre rand	Pre tx	Week 1	Week 2	Week 3	Week 4	Week 5	Week 6	6 weeks	3 months	6 months	9 months	12 months	15 months	18 months	21 months	24 months	30 months	36 months	48 months	60 months	then annually	Relapse
Tumour Biopsy	• [7]																							• [7,8]
2 x Streck Tubes Blood (only for participants currently enrolled in the sub-study)	• [9] Blood sample sub-study closed to new patients in March 2022. Sample collections only applicable for patients already enrolled.				• [9]						• [9]	• [9]		• [9]										• [9]
Consent for microbiome sub-study (separate PIS/ICF)		•																						
Stool sample collection			• [10]								• [10]													
Midstream urine collection			• [10]								• [10]													

[1]within 14 days prior to randomisation.
[2]within 28 days prior to randomisation.
[3]within 63 days prior to randomisation. If the CT scan falls outside this timeframe but a PET CT has been carried out within 42 days prior to randomisation, then there is no requirement to repeat the CT scan as long as the PET CT is a whole body scan.
[4]as a minimum, 1 of the MRI or PET scan (if done) for pelvic staging must be within 42 days before randomisation.
[5]within 10 days prior to start of treatment
[6]until 3 years after the last participant has completed treatment or death
[7]if consented to retrieval of stored material for future cancer research
[8]and where clinically indicated
[9]if consented to biomarkers sub-study and after randomisation (ACT5 only)
[10]if consented to gut microbiome sub-study and after randomisation (ACT5 only)
