# Supplementary material for: Personalising anal cancer radiotherapy dose (PLATO): protocol for a multicentre integrated platform trial
Source: BMJ Open. 2025 Nov 9;15(11):e109655. doi: 10.1136/bmjopen-2025-109655 (PMC12658545; doi:10.1136/bmjopen-2025-109655)
Supplement: Author's manuscript [file bmjopen-2025-109655.draft_revisions.pdf]

BMJ Open is committed to open peer review. As part of this commitment we make the peer review history of every article we publish publicly available.

When an article is published we post the peer reviewers' comments and the authors' responses online. We also post the versions of the paper that were used during peer review. These are the versions that the peer review comments apply to.

The versions of the paper that follow are the versions that were submitted during the peer review process. They are not the versions of record or the final published versions. They should not be cited or distributed as the published version of this manuscript.

BMJ Open is an open access journal and the full, final, typeset and author-corrected version of record of the manuscript is available on our site with no access controls, subscription charges or pay-per-view fees (http://bmjopen.bmj.com).

If you have any questions on BMJ Open's open peer review process please email info.bmjopen@bmj.com

BMJ Open

Personalising anal cancer radiotherapy dose (PLATO): protocol for a multi-centre integrated platform trial

Journal:	*BMJ Open*
Manuscript ID	bmjopen-2025-109655
Article Type:	Protocol
Date Submitted by the Author:	20-Aug-2025
Complete List of Authors:	Frood, Russell; University of Leeds, Leeds Institute of Clinical Trials<br>Gilbert, Alexandra; University of Leeds Leeds Institute of Clinical Trials Research<br>Gilbert, Duncan; University College London, MRC Clinical Trials Unit at UCL<br>Abbott, Natalie; Velindre Cancer Centre, National Radiotherapy Trials QA<br>Richman, Susan; University of Leeds Leeds Institute of Medical Research at St James's; Leeds Biomedical Research Centre<br>Goh, Vicky; King's College London School of Biomedical Engineering & Imaging Sciences, Cancer Imaging<br>Rao, Sheela; Royal Marsden NHS Foundation Trust, Gastrointestinal Unit<br>Webster, Joanne; University of Leeds, Leeds Institute of Clinical Trials Research<br>Smith, Alexandra; University of Leeds, Leeds Institute of Clinical Trials Research<br>Copeland, Joanne; University of Leeds Leeds Institute of Clinical Trials Research<br>Ruddock, Sharon; University of Leeds Leeds Institute of Clinical Trials Research<br>Berkman, Lindy; Patient representative<br>Muirhead, Rebecca ; Oxford University Hospitals NHS Foundation Trust,<br>Renehan, Andrew; University of Manchester, Division of Cancer Sciences; University of Manchester Institute of Population Health, MRC Health eResearch Centre (HeRC), Division of Informatics, Imaging and Data Sciences,<br>Harrison, Mark; Hillingdon Hospitals NHS Foundation Trust<br>Adams, Richard; Velindre Cancer Centre<br>Hawkins, Maria; University College London, Medical Physics and Biomedical Engineering; University College London<br>Brown, Sarah; University of Leeds, Clinical Trials Research Unit<br>Sebag-Montefiore, David ; University of Leeds Faculty of Medicine and Health, Institute of Cancer and Pathology
Keywords:	RADIOTHERAPY, CHEMOTHERAPY, Gastrointestinal tumours < GASTROENTEROLOGY, Treatment Outcome

For peer review only - http://bmjopen.bmj.com/site/about/guidelines.xhtml

Personalising anal cancer radiotherapy dose (PLATO): protocol for a multi-centre integrated platform trial

Russell Frood[1], Alex Gilbert[1], Duncan Gilbert[2], Natalie L Abbott[3], Susan D Richman[4], Vicky Goh[5], Sheela Rao[6], Joanne Webster[1], Alexandra Smith[1], Joanne Copeland[1], Sharon Ruddock[1], Lindy Berkman[7], Rebecca Muirhead[8], Andrew G Renehan[9], Mark Harrison[10], Richard Adams[11], Maria Hawkins[12], Sarah Brown[1*], David Sebag-Montefiore[13*]

*co-lead authors

1. Leeds Cancer Research UK Clinical Trials Unit, Leeds Institute of Clinical Trials Research, University of Leeds, Leeds, UK
2. MRC Clinical Trials Unit, University College London, London, UK
3. National Radiotherapy Trials QA (RTTQA) group, Velindre Cancer Centre, UK
4. Leeds Institute of Medical Research & NIHR Leeds BRC, University of Leeds, Leeds, UK
5. Cancer Imaging, School of Biomedical Engineering and Imaging Sciences, Kings College London, UK
6. Gastrointestinal Unit, The Royal Marsden Hospital - Chelsea, London, UK
7. Patient and Public Involvement Trial Representative, London, UK
8. Oxford University Hospitals NHS Foundation Trust, Oxford, UK
9. Division of Cancer Sciences, School of Medical Sciences, Faculty of Biology, Medicine and Health, University of Manchester, Manchester, UK
10. The Hillingdon Hospital NHS Foundation Trust, Uxbridge, UK
11. Velindre Cancer Centre, Cardiff, UK
12. University College London, Medical Physics and Biomedical Engineering, London, UK
13. Leeds Institute of Medical Research, University of Leeds, Leeds, UK

For peer review only

Abstract

Introduction

The incidence of anal carcinoma is increasing with the current gold standard treatment being chemoradiotherapy. There is currently a wide range in the radiotherapy dose used internationally which may lead to overtreatment of early-stage disease, and potential under treatment of locally advanced disease.

PLATO is an integrated umbrella trial protocol which consists of three trials focussed on assessing risk adapted use of adjuvant low dose chemoradiotherapy in anal margin tumours (ACT3), reduced dose chemoradiotherapy in early anal carcinoma (ACT4) and dose escalated chemoradiotherapy in locally advanced anal carcinoma (ACT5), given with standard concurrent chemotherapy.

Methods and analysis

The primary endpoints of PLATO are locoregional failure (LRF)-free rate for ACT3 and ACT4 and LRF-free survival for ACT5. Secondary objectives include acute and late toxicities, colostomy-free survival and patient reported outcome measures. ACT3 will recruit 90 participants: participants with removed anal tumours with margins ≤1mm will receive lower dose chemoradiotherapy whilst participants with anal tumours with margins >1mm will be observed. ACT4 will recruit 162 participants, randomised on a 1:2 basis to receive either standard-dose Intensity modulated radiotherapy (IMRT) in combination with chemotherapy or reduced-dose IMRT in combination with chemotherapy. ACT5 will recruit 459 participants, randomised on a 1:1:1 basis to receive either standard-dose IMRT in combination with chemotherapy, or one of two increased-dose experimental arms of IMRT with synchronous integrated boost in combination with chemotherapy.

Ethics and dissemination

This study has been approved by Yorkshire & The Humber - Bradford Leeds Research Ethics Committee (ref: 16/YH/0157, IRAS: 204585), July 2016. Results will be disseminated via national and international conferences, peer reviewed journal articles and social media. A plain English report will be shared with the study participants, patients' organisations and media. Trial registration: ISRCTN88455282.

For peer review only

Article Summary

<u>Strengths and limitations</u>

- An umbrella clinical trial allowing concurrent evaluation of treatment across distinct risk groups aiming to personalise radiotherapy dose for patients with loco-regionally confined anal cancer
- PLATO ACT3 is the first multi-centre phase II study to assess use of risk-adapted adjuvant low dose chemoradiotherapy in T1 anal margin tumours.
- PLATO ACT4 is the first randomised phase II study to assess IMRT dose de-escalation in early anal carcinoma.
- The use of patient-reported outcomes (PROs) to collect toxicity outcome will allow for future patient centred care.
- Recruitment limited to ECOG 0–1 or 0–2 patients which likely restricts generalisability to frailer populations.

Keywords

Anal cancer; chemoradiotherapy; radiotherapy dose; HPV biology; locoregional recurrence

For peer review only

Introduction

Anal cancer

The global incidence of anal squamous cell carcinoma is rising with approximately 1600 new cases in the UK [1] with a global incidence of 50,865 cases [2]. Around 90% of cases are associated with prior high-risk HPV infection [3]. Many patients present with local disease, with less than 10% presenting with metastases [2]. The gold standard treatment is chemoradiotherapy (CRT); three randomised trials performed between 1987 and 1994 determined concurrent Mitomycin C (MMC), 5-Fluorouracil (5FU) and radiotherapy (MF-CRT) as the standard of care [4-6]. ACT1, the largest of these, randomised 585 UK patients to radiotherapy alone or MF-CRT and demonstrated CRT significantly reduced locoregional failure (LRF) from 61% to 36% at 3 years [4]. This benefit was maintained after a median follow up of 13 years [7], leading to a change in routine clinical practice with chemoradiotherapy replacing radical surgery as the standard of care [8]. The reduction in LRF was also demonstrated in a parallel European Organisation for Research and Treatment of Cancer (EORTC) trial of 110 patients with locally advanced disease [5] and in a Radiation Therapy Oncology Group (RTOG) trial (n=310) which demonstrated a significant reduction in LRF colostomy-free survival with MF-CRT compared with 5-Fluorouracil and radiotherapy [6].

Three further randomised trials were performed between 1998 and 2008 testing the role of neoadjuvant, concurrent and adjuvant cisplatin schedules [9-11]. The UK ACT2 trial, the largest anal trial [11] enrolled 940 patients and used a continuous two-phase radiotherapy schedule. There was no difference in the complete response rate for concurrent cisplatin compared with mitomycin C, and no improvement in progression-free survival with the addition of two cycles of maintenance 5FU cisplatin. However, outcomes were improved compared to ACT1 and the continuous radiotherapy schedule may have contributed to as other trials included a treatment break prior to the second phase of radiotherapy. The RTOG 9811 trial of 641 patients found that neoadjuvant and concomitant cisplatin 5FU resulted in inferior LRF, colostomy-free survival (CFS) and overall survival when compared with MF-CRT [9, 10]. The ACCORD 03 trial enrolled 307 patients and showed no benefit from the addition of neoadjuvant cisplatin 5FU chemotherapy [12]. It also compared two doses of boost (15 vs 20-25Gy) after whole pelvic irradiation. A non-significant 5% improvement in local control was seen. Following, these trials MMC 5FU CRT remained the standard of care.

The radiotherapy techniques used in previous phase III trials were relatively crude and resulted in substantial irradiation of the surrounding tissue and associated acute and late toxicity. Significant improvement in radiotherapy treatment using Intensity Modulated Radiotherapy (IMRT), allows the

For peer review only

use of altered doses to the gross tumour volume, sparing normal tissues [13]. As IMRT can reduce acute toxicity, this has led a wide range in the radiotherapy dose fractionation used, with doses of 60Gy, or more used in several countries globally. This is likely to result in substantial overtreatment [14, 15].

Although there are clinically derived prognostic biomarkers (T and N stage, male gender, low Hb, and more recently tumour infiltrating lymphocytes) which can help define the risk of relapse, there is currently a lack of biologically derived and validated predictive biomarkers to stratify and personalise treatment [16-20].

We designed a UK umbrella clinical trial to personalise radiotherapy dose across the locoregional disease spectrum.

Methods and analysis

Design and Aim

PLATO is an integrated umbrella trial protocol that employs a master protocol consisting of three risk stratified multi-centre trials (ACT3, ACT4 and ACT5). ACT3 is a non-randomised phase II trial where participants with a locally excised small (<=2cm) anal verge tumour with margins ≤1mm receive lower dose chemoradiotherapy and participants with anal tumour margins >1mm are observed. ACT4 is randomised non-comparative phase II trial where participants are randomised on a 1:2 basis to receive either standard-dose IMRT or reduced-dose IMRT in combination with chemotherapy. ACT5 is a randomised comparative phase II/III trial with an internal pilot where participants are randomised on a 1:1:1 basis to receive either standard-dose IMRT in combination with chemotherapy, or one of two increased-dose experimental arms of IMRT with synchronous integrated boost (SIB) in combination with chemotherapy. An integrated translational programme is included across the trial platform.

A total of 711 eligible participants will be recruited from 36 UK National Health Service sites: 90 to ACT3, 162 to ACT4, and 459 to ACT5. Participant pathway is depicted in Figure 1. The protocol and this manuscript have been written in accordance with the Standard Protocol Items: Recommendations for Interventional Trials guidelines. Recruitment is anticipated to take place over six years. Recruitment opened on 03/01/2017 and closed on 31/08/23 with a total of 709 patients recruited. Follow up for the primary endpoint analysis of each trial is currently ongoing and the first primary endpoint results are expected to be reported in May 2026.

For peer review only

Trial Objectives

The primary objectives are as follows:

ACT3: to assess 3-year LRF-free rate in participants with anal margin tumours treated with local excision.

ACT4: to assess 3-year LRF- free rate in participants with early-stage disease.

ACT5: to assess LRF-free survival in participants with locally advanced disease.

Secondary Objectives

To assess overall (all ACT trials) and between individual trial treatment arms (ACT4 and 5 only):

- Acute and late toxicities
- Treatment compliance
- Clinical response rate
- Disease-free survival (DFS)
- Colostomy-free survival (CFS)
- Progression-free survival (PFS)
- Overall survival (OS)
- Patient Reported Outcome Measures

Translational Objectives

- To assess the prognostic utility of p16/HPV status and tumour infiltrating lymphocyte scores with respect to chemoradiotherapy in Anal Squamous Cell Carcinoma.
- To investigate the nature and performance characteristics of circulating markers of disease (circulating tumour cells and cell free DNA) in response/relapse in high-risk cases (ACT5 patients only).
- To assess the changes in the gut microbiome caused by chemoradiotherapy and develop hypotheses around any potential relationship between this and patients experiencing late (gut) toxicities.

Study population

Patients with locally excised T1 N0 or Nx anal margin tumour and anal canal superficially invasive squamous cell carcinoma (SISCCA) are eligible for ACT3, patients with T1-2 ≤4cm N0 or Nx anal canal or T2 ≤4cm N0 or Nx anal margin (in situ or treated by prior local excision within 3 months before

For peer review only

randomisation) are eligible for ACT4, and patients with T2 N1-3 or T3-4 Nany are eligible for ACT5. ACT3 accepts patients with an Eastern Cooperative Oncology Group (ECOG) performance status of 0-2 whereas for ACT4 or ACT5 patients require an ECOG performance status of 0-1.

Only participants meeting all the inclusion criteria and none of the exclusion criteria will be considered for participation in the trial, with no eligibility waivers permitted. The full inclusion and exclusion criteria for ACT3, ACT4 and ACT5 are set out in **Table 1** and **Table 2**.

Consent and withdrawal

Informed written consent will be given by all participants before entering the study and before any interventions. Consent will be taken by the Principal Investigator or their approved appropriate medically qualified healthcare professional. Optional consent for participants recruited to ACT5 will be sought regarding the participation in the biological serum plasma biomarker and microbiome sub-studies. In the event of withdrawal, any data collected up until that point will be kept and potentially included in analyses. It will be made clear to any participant withdrawing consent that further data pertaining to safety will continue to be collected.

Registration, randomisation and recruitment

Participants will be registered (ACT3) or randomised (ACT4, ACT5) at the University of Leeds Cancer Research UK Clinical Trials Unit (CTU) following confirmation of written consent and eligibility.

ACT3

Participants with anal tumour margins >1mm (deep and lateral) will undergo observation. Participants with anal tumour margins ≤1mm (close or involved), including piecemeal excision, will receive lower-dose CRT.

ACT4

Participants will be randomised on a 1:2 basis (standard-dose:reduced-dose) to receive either standard-dose IMRT or reduce-doe IMRT, both in combination with chemotherapy. A computer-generated minimisation program that incorporates a random element will be used to ensure the treatment groups are well-balanced for the following participant characteristics:

- T-stage (T1, T2)

For peer review only

- N-stage (N0, NX)
- Gender (M, F)
- HIV status (positive, negative)
- Randomising centre

ACT5

For each of the pilot , Phase II and Phase III trial components, patients who fulfil the eligibility criteria and have given written informed consent will be randomised on a 1:1:1 basis to receive either standard-dose IMRT in combination with chemotherapy, or one of two increased-dose experimental arms of IMRT with synchronous integrated boost (SIB) in combination with chemotherapy and will be allocated a trial number. Acomputer-generated minimisation program will be used to balance the following participant characteristics at randomisation:

- T-stage (T2/3, T4)
- N-stage (NX/0/1, N2/3)
- Gender (M, F)
- HIV status (positive, negative)
- Chemotherapy regimen (5FU, Capecitabine)
- Randomising centre

Sample size

The target sample size is 711 participants across each of the three study arms. The justification for the translational research questions sample size is provided in **Supplemental Material 1**.

ACT3

This study is powered to demonstrate that the overall treatment strategy of local excision alone with or without additional lower-dose radiotherapy with chemotherapy, does not produce unacceptable rates of efficacy (i.e. below 80%), but can reach efficacy rates of up to 90% (i.e. LRF rate of 10%). With 80% power and 5% 1-sided significance, 82 patients are required. Allowing for a drop-out rate of 10%, the target sample size is 90 patients, recruited over 3 years.

For peer review only

Inclusion Criteria		
**ACT3**	**ACT4**	**ACT5**
Informed consent	Informed consent	Informed consent
Histologically-proven, invasive primary squamous cell carcinoma of the anus	Histologically-proven, invasive primary squamous cell carcinoma of the anus	Histologically-proven, invasive primary squamous cell carcinoma of the anus
ECOG performance status 0-2	ECOG performance status 0-1	ECOG performance status 0-1
Absolute neutrophil count >1.5 x 109/L; platelets >100 x 109/L	Absolute neutrophil count >1.5 x 109/L; platelets >100 x 109/L 3	Absolute neutrophil count >1.5 x 109/L; platelets >100 x 109/L
Serum transaminase <2x ULN	Serum transaminase <2x ULN	Serum transaminase <2x ULN
Estimated GFR >50mls/min	Estimated GFR >50mls/min	Estimated GFR >50mls/min
Bilirubin <1.5 x ULN	Bilirubin <1.5 x ULN	Bilirubin <1.5 x ULN
HIV negative or HIV positive and receiving effective antiretroviral therapy with supervision and CD4 count >200	HIV negative or HIV positive and receiving effective antiretroviral therapy with supervision and CD4 count >200	HIV negative or HIV positive and receiving effective antiretroviral therapy with supervision and CD4 count >200
T1 N0 or Nx anal margin tumour and anal canal SISCCA treated with local excision within 3 months before registration	T1-2 ≤4cm N0 or Nx7 anal canal<br>or<br>T2 ≤4cm N0 or Nx7 anal margin (in situ or treated by prior local excision within 3 months before randomisation)	T2 N1-3<br>or<br>T3-4 N any
Age 16 or over	Age 16 or over	Age 16 or over (18 in Ireland)
Patient considered fit for either the protocol-defined follow-up, or reduced dose CRT.	Patient considered fit for all ACT4 protocol defined treatments	Patient considered fit for all ACT5 protocol defined treatments
Prepared to practice methods of contraception of proven efficacy during treatment and until 6 months post end of treatment	Prepared to practice methods of contraception of proven efficacy during treatment and until 6 months post end of treatment	Prepared to practice methods of contraception of proven efficacy during treatment and until 6 months post end of treatment
Able to undergo all mandated staging and follow-up investigations, including MRI	Able to undergo all mandated staging and follow-up investigations, including MRI	Able to undergo all mandated staging and follow-up investigations, including MRI

Table 1: Inclusion criteria for the different PLATO studies. CD = cluster of differentiation, ECOG = Eastern Co-operative Oncology Group, GFR = glomerular filtration rate, CRT = chemoradiotherapy, HIV = human immunodeficiency virus, MRI = magnetic resonance imaging.

For peer review only

Exclusion Criteria		
**ACT3**	**ACT4**	**ACT5**
Previous malignancy of pelvic origin where treatment was completed less than 2 years before registration or there is still evidence of disease, or previous untreated malignancy of any origin.	Previous malignancy of pelvic origin where treatment was completed less than 2 years before randomisation or there is still evidence of disease, or previous untreated malignancy of any origin.	Previous malignancy of pelvic origin where treatment was completed less than 2 years before randomisation or there is still evidence of disease, or previous untreated malignancy of any origin.
Prior systemic chemotherapy for anal cancer	Prior systemic chemotherapy for anal cancer	Prior systemic chemotherapy for anal cancer
Prior radiotherapy to the pelvis	Prior radiotherapy to the pelvis	Prior radiotherapy to the pelvis
Uncontrolled cardiorespiratory comorbidity	Uncontrolled cardiorespiratory comorbidity	Uncontrolled cardiorespiratory comorbidity
Pregnant or lactating	Pregnant or lactating	Pregnant or lactating
Immuncompromised (organ transplant)	Immuncompromised (organ transplant)	Immuncompromised (organ transplant)
Requiring ongoing treatment with a contraindicated concomitant medication	Requiring ongoing treatment with a contraindicated concomitant medication	Requiring ongoing treatment with a contraindicated concomitant medication
>1 local excision performed, at different times, for the same lesion		

Table 2: Exclusion criteria for the different PLATO studies.

ACT4

This study is powered to demonstrate that the reduced-dose IMRT arm does not produce unacceptable rates of efficacy (i.e. below 80%) but can reach efficacy rates of up to 90% (i.e. LRF rate of 10%). A target efficacy rate of 90% or greater is considered desirable based on data from the ACT2 trial [10] where 10% of T1-2 N0 patients had a pelvic failure at 3 years post-randomisation. The standard-dose IMRT arm will act as a calibration arm, to ensure the desired efficacy rate is plausible.

With 80% power and 5% 1-sided significance, 123 patients are required with a 1:2 randomisation (standard-dose:reduced-dose). In order to address the research hypothesis for the p16+ subset, the sample size has been inflated by 20% (as 90% of patients are expected to present with this genotype, and a further 90% have samples suitable for analysis). Allowing for a 10% drop-out rate, the target sample size is 162 patients, recruited over 2 years.

For peer review only

ACT5

The pilot stage of ACT5 will recruit a maximum of 60 patients (20:20:20). A formal power calculation was not performed for the pilot study given the aim is to review the initial feasibility of dose escalation and concomitant chemotherapy.

The Phase II trial will recruit an additional 80 patients. The phase II analysis will focus on the acute toxicity data including grade 3 and above neutropenia from the first 140 patients (including the pilot) randomised (overall in the three arms). With 80% power and 5% 1-sided significance, an acceptable rate of grade 3/4 neutropenia of <40% and an unacceptable rate of ≥60% yields a sample size of 126 patients (42:42:42), 140 with 10% drop-out. The desired rate of 40% is based on data from the RTOG 9811, RTOG 0529 and ACT2 trials where rates of grade 3/4 neutropenia were 45%, 58% and 24% respectively.

The overall trial is powered on detecting an absolute difference of 10% in 3-year LRF-free survival between the control and experimental arm(s). This difference is equivalent to a hazard ratio of 0.63 and assumes a LRF rate of 30% (3-year LRF-free survival 70%) in the control arm based on the ACT2 data [10].

If both experimental arms pass the pre-specified boundaries for acceptable toxicity at interim analysis, all three arms will continue into the phase III trial. This would compare each experimental arm against the standard dose in terms of 3-year LRF-free survival. With 80% power and 10% 2-sided significance 459 (153:153:153) patients are required (including 10% drop out) for the primary endpoint analysis. The target event rate is 113 per comparison based on assuming 6 years of recruitment and a minimum of 3 years follow up for all patients.

Treatment regimen

<u>Radiotherapy treatment outlining and planning</u>

Participants will have their radiotherapy planning study performed using a CT acquisition with a slice thickness of no greater than 3mm in a supine position. Patients will be scanned with a comfortably full bladder using local bladder filling protocol. The clinician will determine if additional bolus material is required during scanning. A nationally approved consensus protocol was developed for IMRT (**Supplementary Material 1)**.

For peer review only

Treatment regimen

ACT3

Participants within the observation arm will be observed under local practice but must attend trial follow-up assessments. Patients under the intervention arm will receive reduced dose IMRT (CTV 41.4Gy in 23 fractions(F)) plus MMC capecitabine (**Table 3**).

ACT4

Participants are randomised to receive either to receive either standard-dose IMRT (GTV 50.4Gy, Elective CTV 40Gy (28F)) in combination with chemotherapy or reduced-dose IMRT (GTV 41.4Gy, Elective CTV 34.5Gy (23F)) in combination with chemotherapy (**Table 4**)

ACT5

Patients will receive either standard-dose IMRT (GTV 53.2Gy) or one of two increased-dose experimental arms of IMRT with SIB (GTV 58.8Gy or GTV 61.6Gy) with elective CTV treated to 40Gy (28F)) in combination with chemotherapy. ACT5 will use Mitomycin C combined with either Capecitabine (**Table 5**) or 5 Fluorouracil (5FU) based on hospital preference (**Table 6**).

Trial Assessments and follow up

For an overview of the ACT3, ACT4 and ACT5 assessment schedule please see **Supplementary Material 2**.

Clinician reported toxicity

With the exception of the ACT3 observation arm, assessment of acute toxicities will take place during each week of treatment. In ACT5 only, clinician reported acute toxicities will be captured at 6 weeks, 3 and 6 months post-end of treatment. All adverse reactions, serious adverse reactions and related unexpected serious adverse events will be recorded in ACT5, in ACT 4 grade 2 and above will be recorded and in ACT3 grade 3 and above will be recorded.

Patient reported outcome measures (toxicity and quality of life)

Baseline, acute and late toxicities and assessment of participants' Quality-of-life (QoL) will be performed via the validated EORTC QLQ-C30 and QLQ-ANL27 [21] questionnaires at baseline, in the final week of treatment, at 6 weeks, 6, 12, 24 and 36 months post-end of treatment. ACT3 participants in the observation arm will complete QoL questionnaires at 6 weeks, 6, 12, 24 and 36 months post-

For peer review only

date of registration. Patients will all complete baseline and final week of treatment questionnaires on paper and then could opt to complete on paper or online using REDCAP.

Follow up imaging

For ACT3 an MRI pelvis will be carried out at 12 and 36 months post-treatment.

For ACT4 and ACT5 MRI pelvis will be performed at 3 and 6 months, and a follow-up CT (chest/abdomen/pelvis) will be carried out at 12, 24 and 36 months post-treatment.

Assessment of efficacy

All patients will be followed up to detect recurrence that may occur in the pelvis or as distant metastases. These will be recorded on the relevant CRF and completed at the time of each follow-up visit. All failures that occur within the pelvis up to the level of the sacral promontory are considered loco-regional. The date of failure will be taken as the date of the biopsy, or where this is not available, the data of the first imaging assessment that confirmed the LRF. The presence of distant metastases is determined by cross sectional imaging.

DFS is defined as the time from randomisation to the first documented evidence of pelvic failure which includes: LRFs, distant metastases, new primary tumours or death.

All deaths occurring from the date of registration/randomisation will be recorded.

Ancillary Care

Participants will receive ancillary care beyond the scope of the trial as required. Any adverse events or complications arising from trial participation will be promptly addressed, and necessary medical interventions will be provided at no cost to the participants. Ancillary care will extend to managing conditions unrelated to the trial that may arise during the study period, ensuring the overall well-being of the participants.

Translational research

Baseline and pre-treatment biopsies, and resections and biopsies from recurrent disease will be collected retrospectively for participants. Tumours will be characterised for p16

For peer review only

(immunohistochemistry), HPV status (PCR) and tumour infiltrating lymphocyte scores, and correlated with outcome.

Longitudinal blood (plasma) samples (ACT 5) (pre-treatment, end of treatment, 3 months, 6 months and 12 months post treatment and at relapse) will be used for analysis of cell free DNA (cfDNA) and characterisation and changes in levels of circulating tumour cells and comparison of these with conventional markers of response.

At least 65% of the ACT5 participants will have faecal samples collected prior to commencing chemoradiotherapy and a paired sample 6-12 weeks (+/- 4 weeks) after the end of treatment. The microbiome will be characterized in these samples and will allow for additional biological insights into local immune response and potential factors relating to long term bowel toxicity.

Data collection and management

Participant data will be recorded on trial-specific case reports forms and submitted to the CTU from participating sites. All data will be entered, validated and stored securely by CTU with missing and discrepant data queried by the data management team. Missing data will be followed up until received or confirmed not available. Data items regarding consent, patient safety and the primary endpoint will be subject to manual priority checking.

Only the CTU will have access to the data, prior to analysis and release of trial results. On completion of the trial, data will be stored in the sponsor archiving facility for a minimum of 15 years. After the final trial results have been published, interested researchers may contact the CTRU to request access to relevant data.

Statistical Analysis

Efficacy analyses for ACT3 and ACT5 will be on an intention-to-treat (ITT) basis, including participants according to the treatment arm they were recruited/randomised to. For ACT4 a modified ITT will be used, including participants if they receive at least one dose of trial treatment.

<u>Primary endpoint analysis</u>

LRF includes a failure at the primary site (local) and/or surrounding nodal sites (regional). The event date is the biopsy date where the failure was confirmed histologically, or the scan date used to confirm failure on MDT review if a biopsy date is not available.

For peer review only

In ACT3, analysis will assess the proportion of participants without a LRF at 3-year post-recruitment, and the one-sided 95% CI (corresponding to a two-sided 90% CI) on the whole trial cohort. Assessment of the efficacy of the overall treatment strategy will focus on the lower bound of the CI, and whether it falls below the 'unacceptable' rate of 80%.

In ACT4, analysis will assess the proportion of participants without a LRF in the reduced-dose IMRT arm at 3 years post-recruitment and the one-sided 95% CI. Assessment of the efficacy will focus on whether the lower bound of the CI falls below the 'unacceptable' rate of 80%. The proportion of participants without a LRF in the standard-dose IMRT arm with corresponding CI will be presented to act as a calibration arm, and to establish whether the desired efficacy rate of 90% is plausible. In addition, the analysis will be performed on the subgroup of patients with a p16+ genotype.

In ACT5 LRF-free survival for each experimental arm will be compared against the control, i.e. two separate comparisons, using Cox's proportional hazards (PH) modelling, adjusting for the minimisation factors. The hazard ratio (HR) for the experimental arm versus the control arm will be presented along with two-sided 90% CIs and associated p-value testing. A secondary analysis will compare the combined dose escalation arms with the control to test the hypothesis that dose escalation generally provides improved LRF-free survival compared to control.

LRF-free survival will be presented via Kaplan-Meier (KM) curves. Median LRF-free survival and LRF-free survival estimates at 3 years, with corresponding 90% and 95% CIs will be presented along with the log-rank test statistic (and associated p-value).

Participants who are failure-free at the time of analysis, or who have come off trial prior to observing their primary endpoint will be censored at their last known alive and LRF-free date.

Secondary endpoint analysis

Time-to-event endpoints including CFS, DFS PFS and OS will be presented using KM curves. For ACT5 Cox's PH model will be used to compare each experimental arm with the control. There will be no formal comparison between the arms for ACT3 and ACT4. The proportion of participants experiencing each event will be presented by treatment group (where applicable) and overall.

For peer review only

The proportion of participants experiencing acute toxicities as per the latest National Cancer institute Common Terminology Criteria for Adverse Events (NCI-CTAE) will be summarised for each treatment arm, for the overall treatment period and (for ACT5 only) at 6 weeks and 3 and 6 months. The number and proportion of participants experiencing grade 3 and above acute toxicities will be reported along with 95% CIs. These will be compared between each treatment arm and the control using a multivariable logistic regression model adjusted for the minimisation factors, for ACT5 only. Exploratory comparisons of acute toxicity may be performed between the experimental arms.

The proportion of participants adhering to the radiotherapy schedule (with no greater than 3 days delay due to toxicity) will be reported. In ACT5 only this will be compared between each treatment arm and the control using a logistic regression model.

The proportion of participants achieving a Complete Response (CR) at 3 and 6 months will be presented along with 95% CIs (ACT4 and 5). Complete response is defined as a TRG score of 1 or 2 assessed via MRI imaging, or if clinical assessment only is used to define response, a category of 'Complete response'. A logistic regression model, adjusted for the minimisation factors will be used to compare each treatment arm with the control (ACT 5 only) for the proportion of participants who have and have not achieved a CR at 6 months post-end of treatment.

EORTC QLQ guidelines will be used for analyses and management of missing data.(1) Mean scores and change in mean score from baseline with corresponding 95% CIs will be calculated for all domains of the patient reported questionnaires (EORTC QLQ-C30 and ANL27) for each treatment group and overall, at each follow-up time point.

Descriptive summaries will be presented by treatment arm for the pattern of failure and the proportion of participants with local relapse undergoing salvage surgery.

<u>Translational statistical analysis</u>

Analysis of p16, HPV and LRF

p16/HPV and tumour infiltrating lymphocytes will be analysed as part of the main trial analysis (primary endpoints of 3 year LRF) and as such, results of the biomarker analysis from ACT4 and the pilot/phase II stages of ACT5 will be made available (subject to oversight, see below) with a plan to validate the predictive value in later stages of the platform and inform subsequent biomarker stratification in later stages of the trial.

For peer review only

Analysis of cfDNA

Serial data on CTCs and cfDNA will be analysed for the cohort of patients in ACT5 (with an estimated 25% risk of recurrence at 1 year). Performance characteristics will be determined for these data (CTC and cfDNA) in relation to complete response and 1 year DFS.

Analysis of changes in gut microbiome

The faecal microbiome in patients undergoing radical treatment for anal cancer has not previously been investigated and as such all analyses and comparisons with e.g. long-term toxicity outcome measures are exploratory. Our primary outcome measure is the feasibility of collecting and analysing the faecal microbiome in patients enrolled in ACT5 with respect to changes in microbiome/metabolomics profiles pre- and post-chemoradiotherapy.

Trial oversight

The Chief Investigator (CI) will have overall responsibility for the trial design, set-up and management and will be supported by 5 study-specific clinical Leads across the 3 trials. A trial management group (TMG) comprising of the CI, CTU team, a PPIE representative (LB), co-leads for each trial, representatives for medical oncology, medical imaging, radiotherapy QA, translational research and patient reported outcomes and key external members of staff will meet 3-6 monthly. A trial steering committee will provide overall supervision of the trial, in particular trial progress, adherence to protocol, participant safety and consideration of new information. It will include an Independent Chair, not less than two other independent members and a PPI representative. An independent data monitoring and ethics committee (DMEC), which will meet annually as a minimum, will review safety and ethics of the trial during recruitment and follow-up period.

Patient and Public Involvement and Engagement (PPIE)

Input from the Leeds Cancer Centre PPIE team, National Cancer Research Institute consumer representatives and Leeds Radiotherapy Research user group was sought, and the information gained acted on through every aspect of the protocol design, the patient information leaflet, and the lay summary.

Ethics and dissemination

For peer review only

This study has been approved by the Yorkshire & The Humber - Bradford Leeds Research Ethics Committee (ref: 16/YH/0157, IRAS: 204585), July 2016.

The trial results will be analysed in stages for short-term and long-term endpoints. Results will be disseminated via national and international conferences, peer reviewed journal articles as well as social media and a newsletter to the relevant stakeholders. The final trial publications will be written by the TMG and will adhere to International Committee of Medical Journal Editors guidelines.

Discussion

The incidence of anal carcinoma is rising globally with a range of radiotherapy doses being employed to treat patients without high levels of evidence. PLATO is an integrated umbrella trial which aims to personalise radiotherapy dose for locoregionally confined cancer with arms looking at anal margin tumours, early and advanced anal carcinoma with it being the multi-centre phase II (ACT3 and ACT4) and phase II/III (ACT5) to do so. It is also the first anal cancer trial to use the EORTC QLG-ANL27 questionnaire and has translational components of the study for a subset of patients will add insight into HPV status and circulating tumour markers on disease free survival, and the changes in the gut microbiome and how this may relate to bowel toxicity.

Since the development of the protocol, the core outcome research measures in anal cancer (CORMAC) [22] has been published with the outcomes in the original protocol being retained but the planned analyses will align with the CORMAC recommendations. PLATO is focussed on personalisation of treatment for patients with a good ECOG performance status and highlights the need for further research into the optimal treatment for elderly and frail patients [23]. As part of this a need for a reliable surrogate for the current performance status which is not subjective and can take into account changes in patients physical state is needed [24]. Medical imaging-based body composition analysis, which is the process of quantifying the volume, density and ratio of adipose tissue, skeletal muscle, bone and soft tissues within the abdomen can provide additional valuable patient-specific metrics which could be used as an indicator or frailty or outcome [25]. Future translational work looking into the imaging biomarkers as part of the PLATO study will be explored and will be invaluable in any future trial design.

Patient consent for publication

Not applicable.

For peer review only

References

[1] Anal cancer incidence statistics (2021) Cancer Research UK. Available at: https://www.cancerresearchuk.org/health-professional/cancer-statistics/statistics-by-cancer-type/anal-cancer/incidence (Accessed: 01 August 2024).

[2] Huang J, Lok V, Zhang L, et alIDDF2022-ABS-0273â€…Global incidence of anal cancer by histological subtypes. Gut 2022;71:A179-A180.

[3] Baricevic I, He X, Chakrabarty B, et al. High-sensitivity human papilloma virus genotyping reveals near universal positivity in anal squamous cell carcinoma: different implications for vaccine prevention and prognosis. Eur J Cancer. 2015;51(6):776-85.

[4] UKCCCR Anal Cancer Trial Working Party. Epidermoid anal cancer: results from the UKCCCR randomised trial of radiotherapy alone versus radiotherapy, 5-fluorouracil, and mitomycin. Lancet. 1996;348(9034):1049-54.

[5] Bartelink H, Roelofsen F, Eschwege F, et al. Concomitant radiotherapy and chemotherapy is superior to radiotherapy alone in the treatment of locally advanced anal cancer: results of a phase III randomized trial of the European Organization for Research and Treatment of Cancer Radiotherapy and Gastrointestinal Cooperative Groups. J Clin Oncol 1997;15:2040-9.

[6] Flam M, John M, Pajaket TF, et al. Role of mitomycin in combination with fluorouracil and radiotherapy, and of salvage chemoradiation in the definitive nonsurgical treatment of epidermoid carcinoma of the anal canal: results of a phase III randomized intergroup study. J Clin Oncol 1996;14:2527-39.

[7] Northover J, Glynne-Jones R, Sebag-Montefiore D, et al. Chemoradiation for the treatment of epidermoid anal cancer: 13-year follow-up of the first randomised UKCCCR Anal Cancer Trial (ACT I). Br J Cancer 2010;30;102(7):1123-8.

[8] Downing A, Morris EJ, Aravani A, et al. The Effect of the UK Coordinating Centre for Cancer Research Anal Cancer Trial (ACT1) on Population-based Treatment and Survival for Squamous Cell Cancer of the Anus. Br J Cancer 2010;102(7):1123-8.

For peer review only

[9] Ajani JA, Winter KA, Gunderson LL, et al. Fluorouracil, mitomycin, and radiotherapy vs fluorouracil, cisplatin, and radiotherapy for carcinoma of the anal canal: a randomized controlled trial. JAMA 2008;299:1914-21.

[10] Gunderson LL, Winter KA, Ajaniet JA, al. Long-term update of US GI intergroup RTOG 98-11 phase III trial for anal carcinoma: survival, relapse, and colostomy failure with concurrent chemoradiation involving fluorouracil/mitomycin versus fluorouracil/cisplatin. J Clin Oncol 2012;30(35):4344-51.

[11] James RD, Glynne-Jones R, Meadows HM, et al. Mitomycin or cisplatin chemoradiation with or without maintenance chemotherapy for treatment of squamous-cell carcinoma of the anus (ACT II): a randomised, phase 3, open-label, 2 x 2 factorial trial. Lancet Oncol 2013;14:516-24.

[12] Peiffert D, Tournier-Rangeard L, Gérard J-P, et al. Induction chemotherapy and dose intensification of the radiation boost in locally advanced anal canal carcinoma: final analysis of the randomized UNICANCER ACCORD 03 trial. J Clin Oncol 2012;30:1941-8.

[13] Muirhead R, Adams RA, Gilbert DC, et al. Anal cancer: developing an intensity-modulated radiotherapy solution for ACT2 fractionation. Clin Oncol (R Coll Radiol) 2014;26(11):720-1.

[14] Muirhead R, Partridge M, Hawkins MA. A tumor control probability model for anal squamous cell carcinoma. Radiother Oncol 2015;116(2):192-6.

[15] Gilbert A, Drinkwater K, McParland L, et al. UK national cohort of anal cancer treated with intensity-modulated radiotherapy: One-year oncological and patient-reported outcomes. Eur J Cancer 2020;128:7-16.

[16] Obermueller T, Hautekiet J, Busto MP, et al. Prognostic value of high-risk human papillomavirus DNA and p16INK4a immunohistochemistry in patients with anal cancer: An individual patient data meta-analysis. Eur J Cancer 2021;157:165-178.

[17] Ang KK, Harris J, Wheeler R, et al. Human papillomavirus and survival of patients with oropharyngeal cancer. N Engl J Med 2010;363(1):24-35.

For peer review only

[18] Wakeham K, Murray L, Muirhead R, et al. Multicentre Investigation of Prognostic Factors Incorporating p16 and Tumour Infiltrating Lymphocytes for Anal Cancer After Chemoradiotherapy. Clin Oncol (R Coll Radiol) 2021;33(10):638-649.

[19] Le JY, Cutts RJ, White I, et al. Next Generation Sequencing Assay for Detection of Circulating HPV DNA (cHPV-DNA) in Patients Undergoing Radical (Chemo)Radiotherapy in Anal Squamous Cell Carcinoma (ASCC). Front Oncol 2020;10:505.

[20] Jones CM, Goh V, Sebag-Montefiore D, Gilbert DC. Biomarkers in anal cancer: from biological understanding to stratified treatment. Br J Cancer. 2017 Jan 17;116(2):156-162

[21] Sodergren SC, Johnson CD, Gilbert A, et al. European Organisation for Research and Treatment of Cancer Quality of Life Group. International Validation of the EORTC QLQ-ANL27, a Field Study to Test the Anal Cancer-Specific Health-Related Quality-of-Life Questionnaire. Int J Radiat Oncol Biol Phys 2023;115(5):1155-1164.

[22] Fish R, Sanders C, Adams R, et al. A core outcome set for clinical trials of chemoradiotherapy interventions for anal cancer (CORMAC): a patient and health-care professional consensus. Lancet Gastroenterol Hepatol 2018;3(12):865-873.

[23] Charnley N, Choudhury A, Chesser P, Cooper RA, Sebag-Montefiore D. Effective treatment of anal cancer in the elderly with low-dose chemoradiotherapy. Br J Cancer. 2005 Apr 11;92(7):1221-5.

[24] Simcock R, Wright J. Beyond Performance Status. Clin Oncol (R Coll Radiol). 2020 Sep;32(9):553-561.

[25] 2. Shah UA, Ballinger TJ, Bhandari R, et al. Imaging modalities for measuring body composition in patients with cancer: opportunities and challenges. J Natl Cancer Inst Monogr. 2023; 2023(61): 56-67.

Contributors

Contributors to the conception and design: AG, DG, NLA, SDR, VG, SR, JW, AS, JC, SR, LB, RM, AGR, MH, RA, MH, SB, DS-M. Development of the protocol, trial documents and patient information sheet: AG, DG, NLA, SDR, VG, SR, JW, AS, JC, SR, LB, RM, AGR, MH, RA, MH, SB, DS-M. Writing the manuscript: RF, SB, DS-M Reviewed the manuscript: All authors. All authors read and approved the final manuscript.

For peer review only

Funding

PLATO is supported by Cancer Research UK [A19121] and Stand Up to Cancer. The study is coordinated by University of Leeds CTRU and sponsored by University of Leeds (GA18/118305). For enquires, trial contact email: plato@leeds.ac.uk. Trial website: https://ctru.leeds.ac.uk/plato.

Competing interests

AGR is supported by the NIHR Manchester Biomedical Research Centre (NIHR203308)

SDR is supported in part by the National Institute for Health and Care Research (NIHR) Leeds Biomedical Research Centre (BRC) (NIHR203331). The views expressed are those of the author and not necessarily those of the NHS, the NIHR or the Department of Health and Social Care.

MH is supported by funding from UCLH National Institute for Health and Care Research Biomedical Research Centre.

For peer review only

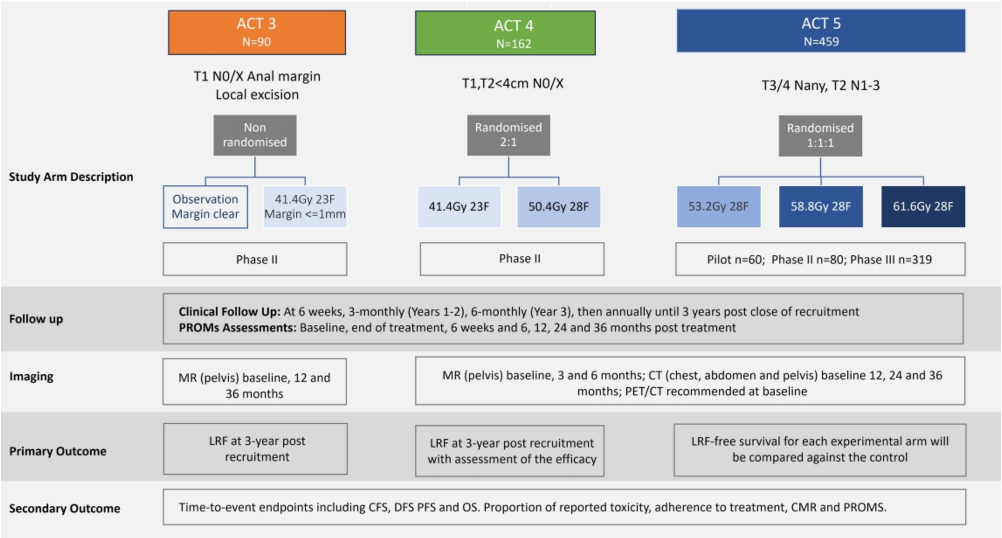

Figure 1: Participant trial pathway

370x199mm (300 x 300 DPI)

Translational research sample size

Analysis of p16, HPV and loco-regional failure

The sample size calculations in ACT4 are inflated to allow for a pre-planned analysis of p16/HPV. With 108 in the dose de-escalation arm of ACT4, we will have around 88% power to the test the dose de-escalation strategy and demonstrate efficacy rates of up to 90%. Assuming only 90% of these patients will have samples suitable for analysis, and 85% will be p16+, we will have 80% power to demonstrate efficacy rates of up to 90% within the p16+ve sub-group. With respect to the ACT5 question around dose escalation, 140 patients in the pilot and phase II stages of ACT5 give approximately 94 patients in the dose escalated arms. Assuming a relapse rate of 30%, where 85% are p16+ve (n=80) and 15% p16-ve (n=14), there would be 94% power to detect a HR of 4.5 between the p16 +ve/-ve groups. With a lower relapse rate of 20% in the dose escalated arms (as targeted within ACT5), the power would reduce to 85%. With 90% of samples suitable for analysis (so 126 patients with samples at the end of phase II, 84 in the dose escalated arm) and assuming a relapse rate of 30%, 85% p16+ve (n=71), and 15% p16-ve (n=13), there would be 90% power to detect a HR of 4.5 between the p16 +ve/-ve groups. If we assumed a relapse rate of 20% in the dose escalated arms (which is what were are targeting in ACT5), the power would reduce to 78%. The power to demonstrate a relationship between TIL score and relapse within p16 +ve patients is about 80% for a relapse rate of 30% and just over 50% for a relapse rate of 20%.

Analysis of cfDNA

Serial data on CTCs and cfDNA (presence/absence and enumeration/ quantification) will be analysed for the cohort of patients in ACT5 (phase II/III) with an estimated 25% risk of recurrence at 1 year. Performance characteristics (including ROC) will be determined for these data (CTC and cfDNA) in relation to complete response and 1 year disease free survival. For such longitudinal monitoring power calculation methodology is less clearly developed, but with 6 marker values throughout the follow-up period on 80 patients we will only be powered to detect relatively large effects.

Analysis of changes in gut microbiome

The faecal microbiome in patients undergoing radical treatment for anal cancer has not previously been investigated and as such all analyses and comparisons are exploratory. Our primary outcome measure is the feasibility of collecting and analysing the faecal microbiome in patients enrolled in ACT5 in >65% cases, i.e. obtaining paired, evaluable samples in at least 65% of patients. If we recruit at least 55 patients into the faecal microbiome sub-study, evaluable paired samples in 42 of these will give us

For peer review only

80% power (at the 1-sided 5% significance level) to achieve this, assuming a true paired, evaluable rate of 80%.

Target volume definition

Gross Tumour Volume (GTV) – ACT3, ACT4 and ACT 5 patients

The GTV will be determined by the treating clinician using the planning CT, and information from clinical data and diagnostic imaging.

- GTV_A = Site of primary tumour and scar (ACT3)
- GTV_A = Includes the gross primary anal tumour volume (ACT4 and ACT5)
- GTV_N = Includes all involved nodes ≤3cm (ACT5)
- GTV_N3 = Includes all involved nodes >3cm (ACT5)

Clinical Target Volume (CTV) – ACT3, ACT4 and ACT5 patients

All the expansions are in 3 dimensions unless stated otherwise.

- CTV_A = GTV_A + 10mm (ACT3)
- CTV_A = GTV_A + 10mm (ACT4)
- CTV_A = GTV_A +15mm (ACT5)

This is manually enlarged to ensure coverage of the anal canal, anal verge, internal and external anal sphincters. In the absence of bony involvement this is then edited to exclude bone.

- CTV_N = GTV_N + 5mm. This is also edited off bone and muscles in the absence of bony involvement (ACT5)
- CTV_E = Elective nodal regions (ACT4 and ACT5).

Planning Target Volume (PTV) – ACT3, ACT4 and ACT5 patients

All the expansions are in 3 dimensions unless stated otherwise.

ACT3 patients:-

- PTV_A= CTV_A + 10mm

ACT4 patients:-

- PTV_A= CTV_A + 10mm

For peer review only

- PTV_E = CTV_E + 5mm

ACT5 patients:-

- PTV_A= CTV_A + 10mm
- PTV_N = CTV_N + 5mm
- PTV_E = CTV_E + 5mm

For peer review only

ACT3 Assessment schedule

ACT3	Baseline			Treatment					Follow-up from end of treatment (or date of registration for observational arm)														
	Eligibility assessments	Pre reg	Pre tx	Week 1	Week 2	Week 3	Week 4	Week 5	6 weeks	3 months	6 months	9 months	12 months	15 months	18 months	21 months	24 months	30 months	36 months	48 months	60 months	then annually[5]	Relapse
Medical history	•																						
ECOG PS, Vital Signs, ht/wt	•[1]																						
Physical exam	•								•*	•	•	•	•	•	•	•	•	•	•	•	•		
Tumour biopsy	•																						
Pregnancy test	•*[1]																						
Full Blood Count:	•[1]		•*[4]	•*	•*	•*	•*	•*															
U&E	•[1]		•*[4]	•*	•*	•*	•*	•*															
LFTs	•[1]		•*[4]	•*	•*	•*	•*	•*															
HIV	•*[2]	•**[2]																					
CD4 count if HIV +ve	•*[2]	•**[2]																					
ECG	•*[1]																						
MRI scan	•[3]												•						•				
Informed consent		•																					
Registration		•																					
QoL		•						•*	• CTRU to administer		• CTRU to administer		• CTRU to administer				• CTRU to administer		• CTRU to administer				
Radiotherapy planning scan		•*																					
Data collection	•	•	•*	•*	•*	•*	•*	•*		•	•	•	•	•	•	•	•	•	•	•	•	•	
CTCAE acute toxicity monitoring				•*	•*	•*	•*	•*															
SAR monitoring and reporting				Monitor during weeks of treatment*																			

Translational research samples (explicit consent required)

Tumour Biopsy	•[6]																						•[6,7]

*RT arm only

** observation arm only

[1]within 14 days prior to registration; [2]within 28 days prior to registration

[3]within 63 days prior to registration

[4]within 10 days prior to start of treatment

[5]until 3 years after the last participant has completed treatment or death

[6]if consented to retreival of stored material for future cancer research [7]and where clinically indicated

For peer review only

ACT4 Assessment schedule

	Baseline			Treatment						Follow-up from end of treatment															
**ACT4**	Eligibility assessments	Pre rand	Pre tx	Week 1	Week 2	Week 3	Week 4	Week 5	Week 6	6 weeks	3 months	6 months	9 months	12 months	15 months	18 months	21 months	24 months	30 months	36 months	48 months	60 months	then annually[6]	Relapse
Medical history	•																							
ECOG PS, Vital Signs, ht/wt	•[1]																							
Physical exam	•									•	•	•	•	•	•	•	•	•	•	•	•	•		
Tumour Biopsy	•																							
Pregnancy test	•[1]																							
Full Blood Count	•[1]		•[5]	•	•	•	•	•	•*															
U&E	•[1]		•[5]	•	•	•	•	•	•*															
LFTs	•[1]		•[5]	•	•	•	•	•	•*															
HIV	•[2]																							
CD4 count if HIV +ve	•[2]																							
ECG	•[1]																							
CT scan	•[3]													•				•		•				
MRI scan	•[4]										•	•												
PET scan	•[4] Strongly recommended																							
Informed consent		•																						
Randomisation		•																						
QoL		•						• 41.4Gy arm only	• 50.4Gy arm only	• CTRU to administer		• CTRU to administer		• CTRU to administer				• CTRU to administer		• CTRU to administer				
Radiotherapy planning scan		•																						
Data collection	•	•	•	•	•	•	•	•	•*		•	•	•	•	•	•	•	•	•	•	•	•	•	
CTCAE acute toxicity monitoring				•	•	•	•	•	•*															
SAR monitoring and reporting				Monitor during weeks of treatment																				
Response assessment											•	•												

Translational research samples (explicit consent required)

	Eligibility assessments	Pre rand	Pre tx	Week 1	Week 2	Week 3	Week 4	Week 5	Week 6	6 weeks	3 months	6 months	9 months	12 months	15 months	18 months	21 months	24 months	30 months	36 months	48 months	60 months	then annually	Relapse
Tumour Biopsy	•[7]																							•[7,8]

*50.4Gy arm only

[1]within 14 days prior to randomisation; [2]within 28 days prior to randomisation

[3]within 63 days prior to randomisation. If the CT scan falls outside this timeframe but a PET CT has been carried out, there is no requirement to repeat the CT scan as long as the PET CT is a whole body scan.

[4]as a minimum, 1 of the MRI or PET scan (if done) for pelvic staging must be within 42 days before randomisation

[5]within 10 days prior to start of treatment

[6]until 3 yearsafter the last participant has completed treatment or death

[7]if consented to retreival of stored material for future cancer research [8]and where clinically indicated

For peer review only

ACT5 Assessment schedule

ACT5	Baseline			Treatment						Follow-up from end of treatment														
	Eligibility assessments	Pre rand	Pre tx	Week 1	Week 2	Week 3	Week 4	Week 5	Week 6	6 weeks	3 months	6 months	9 months	12 months	15 months	18 months	21 months	24 months	30 months	36 months	48 months	60 months	then annually[6]	Relapse
Medical history	•																							
ECOG PS, Vital Signs, ht/wt	•[1]																							
Physical exam	•									•	•	•	•	•	•	•	•	•	•	•	•	•		
Tumour Biopsy	•																							
Pregnancy test	•[1]																							
Full Blood Count	•[1]		•[5]	•	•	•	•	•	•															
U&E	•[1]		•[5]	•	•	•	•	•	•															
LFTs	•[1]		•[5]	•	•	•	•	•	•															
HIV	•[2]																							
CD4 count if HIV +ve	•[2]																							
ECG	•[1]																							
CT scan	•[3]													•				•		•				
MRI scan	•[4]										•	•												
PET scan	•[4] Strongly recommended																							
Informed consent		•																						
Randomisation		•																						
QoL		•							•	• CTRU to administer		• CTRU to administer		• CTRU to administer				• CTRU to administer		• CTRU to administer				
Radiotherapy planning scan		•																						
Data collection	•	•	•	•	•	•	•	•	•	•	•	•	•	•	•	•	•	•	•	•	•	•	•	
CTCAE acute toxicity monitoring				•	•	•	•	•	•	•	•	•												
SAR monitoring and reporting				Monitor during weeks of treatment and until 6 months post end of treatment																				
Response assessment											•	•												

Translational research samples (explicit consent required)

	Eligibility assessments	Pre rand	Pre tx	Week 1	Week 2	Week 3	Week 4	Week 5	Week 6	6 weeks	3 months	6 months	9 months	12 months	15 months	18 months	21 months	24 months	30 months	36 months	48 months	60 months	then annually	Relapse
Tumour Biopsy	•[7]																							•[7,8]
2 x Streck Tubes Blood (only for participants currently enrolled in the sub-study)	Blood sample sub-study closed to new patients in March 2022. Sample collections only applicable for patients already enrolled.										•[9]	•[9]		•[9]										•[9]
Consent for micobiome sub-study (separate PIS/ICF)		•																						
Stool sample collection			•[10]								•[10]													
Midstream urine collection			•[10]								•[10]													

[1]within 14 days prior to randomisation; [2]within 28 days prior to randomisation

[3]within 63 days prior to randomisation. If the CT scan falls outside this timeframe but a PET CT has been carried out within 42 days prior to randomisation, then there is no requirement to repeat the CT scan as long as the PET CT is a whole body scan.

[4]as a minimum, 1 of the MRI or PET scan (if done) for pelvic staging must be within 42 days before randomisation

[5]within 10 days prior to start of treatment

[6]until 3 yearsafter the last participant has completed treatment or death

[7]if consented to retreival of stored material for future cancer research [8]and where clinically indicated

[9]if consented to biomarkers sub-study and after randomisation (ACT5 only)

[10]if consented to gut microbiome sub-study and after randomisation (ACT5 only)

For peer review only

Personalising anal cancer radiotherapy dose (PLATO): protocol for a multi-centre integrated platform trial

Journal:	*BMJ Open*
Manuscript ID	bmjopen-2025-109655.R1
Article Type:	Protocol
Date Submitted by the Author:	18-Oct-2025
Complete List of Authors:	Frood, Russell; University of Leeds, Leeds Institute of Clinical Trials<br>Gilbert, Alexandra; University of Leeds Leeds Institute of Clinical Trials Research<br>Gilbert, Duncan; University College London, MRC Clinical Trials Unit at UCL<br>Abbott, Natalie; Velindre Cancer Centre, National Radiotherapy Trials QA<br>Richman, Susan; University of Leeds Leeds Institute of Medical Research at St James's; Leeds Biomedical Research Centre<br>Goh, Vicky; King's College London School of Biomedical Engineering & Imaging Sciences, Cancer Imaging<br>Rao, Sheela; Royal Marsden NHS Foundation Trust, Gastrointestinal Unit<br>Webster, Joanne; University of Leeds, Leeds Institute of Clinical Trials Research<br>Smith, Alexandra; University of Leeds, Leeds Institute of Clinical Trials Research<br>Copeland, Joanne; University of Leeds Leeds Institute of Clinical Trials Research<br>Ruddock, Sharon; University of Leeds Leeds Institute of Clinical Trials Research<br>Berkman, Lindy; Patient representative<br>Muirhead, Rebecca ; Oxford University Hospitals NHS Foundation Trust,<br>Renehan, Andrew; University of Manchester, Division of Cancer Sciences; University of Manchester Institute of Population Health, MRC Health eResearch Centre (HeRC), Division of Informatics, Imaging and Data Sciences,<br>Harrison, Mark; Hillingdon Hospitals NHS Foundation Trust<br>Adams, Richard; Velindre Cancer Centre<br>Hawkins, Maria; University College London, Medical Physics and Biomedical Engineering; University College London<br>Brown, Sarah; University of Leeds, Clinical Trials Research Unit<br>Sebag-Montefiore, David ; University of Leeds Faculty of Medicine and Health, Institute of Cancer and Pathology
**Primary Subject Heading**:	Oncology
Secondary Subject Heading:	Gastroenterology and hepatology
Keywords:	RADIOTHERAPY, CHEMOTHERAPY, Gastrointestinal tumours < GASTROENTEROLOGY, Treatment Outcome, Clinical Trial, Adult radiotherapy < RADIOTHERAPY

Personalising anal cancer radiotherapy dose (PLATO): protocol for a multi-centre integrated platform trial

Russell Frood[1], Alex Gilbert[1], Duncan Gilbert[2], Natalie L Abbott[3], Susan D Richman[4], Vicky Goh[5], Sheela Rao[6], Joanne Webster[1], Alexandra Smith[1], Joanne Copeland[1], Sharon Ruddock[1], Lindy Berkman[7], Rebecca Muirhead[8], Andrew G Renehan[9], Mark Harrison[10], Richard Adams[11], Maria Hawkins[12], Sarah Brown[1*], David Sebag-Montefiore[13*]

*co-lead authors

1. Leeds Cancer Research UK Clinical Trials Unit, Leeds Institute of Clinical Trials Research, University of Leeds, Leeds, UK
2. MRC Clinical Trials Unit, University College London, London, UK
3. National Radiotherapy Trials QA (RTTQA) group, Velindre Cancer Centre, UK
4. Leeds Institute of Medical Research & NIHR Leeds BRC, University of Leeds, Leeds, UK
5. Cancer Imaging, School of Biomedical Engineering and Imaging Sciences, Kings College London, UK
6. Gastrointestinal Unit, The Royal Marsden Hospital - Chelsea, London, UK
7. Patient and Public Involvement Trial Representative, London, UK
8. Oxford University Hospitals NHS Foundation Trust, Oxford, UK
9. Division of Cancer Sciences, School of Medical Sciences, Faculty of Biology, Medicine and Health, University of Manchester, Manchester, UK
10. The Hillingdon Hospital NHS Foundation Trust, Uxbridge, UK
11. Velindre Cancer Centre, Cardiff, UK
12. University College London, Medical Physics and Biomedical Engineering, London, UK
13. Leeds Institute of Medical Research, University of Leeds, Leeds, UK

For peer review only

Abstract

Introduction

The incidence of anal carcinoma is increasing with the current gold standard treatment being chemoradiotherapy. There is currently a wide range in the radiotherapy dose used internationally which may lead to overtreatment of early-stage disease, and potential under treatment of locally advanced disease.

PLATO is an integrated umbrella trial protocol which consists of three trials focussed on assessing risk adapted use of adjuvant low dose chemoradiotherapy in anal margin tumours (ACT3), reduced dose chemoradiotherapy in early anal carcinoma (ACT4) and dose escalated chemoradiotherapy in locally advanced anal carcinoma (ACT5), given with standard concurrent chemotherapy.

Methods and analysis

The primary endpoints of PLATO are locoregional failure (LRF)-free rate for ACT3 and ACT4 and LRF-free survival for ACT5. Secondary objectives include acute and late toxicities, colostomy-free survival and patient reported outcome measures. ACT3 will recruit 90 participants: participants with removed anal tumours with margins ≤1mm will receive lower dose chemoradiotherapy whilst participants with anal tumours with margins >1mm will be observed. ACT4 will recruit 162 participants, randomised on a 1:2 basis to receive either standard-dose Intensity modulated radiotherapy (IMRT) in combination with chemotherapy or reduced-dose IMRT in combination with chemotherapy. ACT5 will recruit 459 participants, randomised on a 1:1:1 basis to receive either standard-dose IMRT in combination with chemotherapy, or one of two increased-dose experimental arms of IMRT with synchronous integrated boost in combination with chemotherapy.

Ethics and dissemination

This study has been approved by Yorkshire & The Humber - Bradford Leeds Research Ethics Committee (ref: 16/YH/0157, IRAS: 204585), July 2016. Results will be disseminated via national and international conferences, peer reviewed journal articles and social media. A plain English report will be shared with the study participants, patients' organisations and media. Trial registration: ISRCTN88455282.

For peer review only

Article Summary

Strengths and limitations

- An umbrella clinical trial allowing concurrent evaluation of treatment across distinct risk groups aiming to personalise radiotherapy dose for patients with loco-regionally confined anal cancer
- Integrated translational sub-studies enable correlation of biological, genomic, and microbiome data with clinical outcomes using prospectively collected samples.
- Prospective collection of both clinician and patient reported toxicity provides comprehensive assessment of treatment tolerability.
- Centralised radiotherapy quality assurance and standardised IMRT protocols ensure treatment consistency between sites.
- Recruitment limited to ECOG 0–1 or 0–2 patients which likely restricts generalisability to frailer populations.

Keywords

Anal cancer; chemoradiotherapy; radiotherapy dose; HPV biology; locoregional recurrence

For peer review only

Introduction

Anal cancer

The global incidence of anal squamous cell carcinoma is rising with approximately 1600 new cases in the UK [1] with a global incidence of 50,865 cases [2]. Around 90% of cases are associated with prior high-risk HPV infection [3]. Many patients present with local disease, with less than 10% presenting with metastases [2]. The gold standard treatment is chemoradiotherapy (CRT); three randomised trials performed between 1987 and 1994 determined concurrent Mitomycin C (MMC), 5-Fluorouracil (5FU) and radiotherapy (MF-CRT) as the standard of care [4-6]. ACT1, the largest of these, randomised 585 UK patients to radiotherapy alone or MF-CRT and demonstrated CRT significantly reduced locoregional failure (LRF) from 61% to 36% at 3 years [4]. This benefit was maintained after a median follow up of 13 years [7], leading to a change in routine clinical practice with chemoradiotherapy replacing radical surgery as the standard of care [8]. The reduction in LRF was also demonstrated in a parallel European Organisation for Research and Treatment of Cancer (EORTC) trial of 110 patients with locally advanced disease [5] and in a Radiation Therapy Oncology Group (RTOG) trial (n=310) which demonstrated a significant reduction in LRF colostomy-free survival with MF-CRT compared with 5-Fluorouracil and radiotherapy [6].

Three further randomised trials were performed between 1998 and 2008 testing the role of neoadjuvant, concurrent and adjuvant cisplatin schedules [9-11]. The UK ACT2 trial, the largest anal trial [11] enrolled 940 patients and used a continuous two-phase radiotherapy schedule. There was no difference in the complete response rate for concurrent cisplatin compared with mitomycin C, and no improvement in progression-free survival with the addition of two cycles of maintenance 5FU cisplatin. However, outcomes were improved compared to ACT1 and the continuous radiotherapy schedule may have contributed to as other trials included a treatment break prior to the second phase of radiotherapy. The RTOG 9811 trial of 641 patients found that neoadjuvant and concomitant cisplatin 5FU resulted in inferior LRF, colostomy-free survival (CFS) and overall survival when compared with MF-CRT [9, 10]. The ACCORD 03 trial enrolled 307 patients and showed no benefit from the addition of neoadjuvant cisplatin 5FU chemotherapy [12]. It also compared two doses of boost (15 vs 20-25Gy) after whole pelvic irradiation. A non-significant 5% improvement in local control was seen. Following, these trials MMC 5FU CRT remained the standard of care.

The radiotherapy techniques used in previous phase III trials were relatively crude and resulted in substantial irradiation of the surrounding tissue and associated acute and late toxicity. Significant improvement in radiotherapy treatment using Intensity Modulated Radiotherapy (IMRT), allows the

For peer review only

use of altered doses to the gross tumour volume, sparing normal tissues [13]. As IMRT can reduce acute toxicity, this has led a wide range in the radiotherapy dose fractionation used, with doses of 60Gy, or more used in several countries globally. This is likely to result in substantial overtreatment [14, 15].

Although there are clinically derived prognostic biomarkers (T and N stage, male gender, low Hb, and more recently tumour infiltrating lymphocytes) which can help define the risk of relapse, there is currently a lack of biologically derived and validated predictive biomarkers to stratify and personalise treatment [16-20].

We designed a UK umbrella clinical trial to personalise radiotherapy dose across the locoregional disease spectrum.

Methods and analysis

Design and Aim

PLATO is an integrated umbrella trial protocol that employs a master protocol consisting of three risk stratified multi-centre trials (ACT3, ACT4 and ACT5). ACT3 is a non-randomised phase II trial where participants with a locally excised small (<=2cm) anal verge tumour with margins ≤1mm receive lower dose chemoradiotherapy and participants with anal tumour margins >1mm are observed. ACT4 is randomised non-comparative phase II trial where participants are randomised on a 1:2 basis to receive either standard-dose IMRT or reduced-dose IMRT in combination with chemotherapy. ACT5 is a randomised comparative phase II/III trial with an internal pilot where participants are randomised on a 1:1:1 basis to receive either standard-dose IMRT in combination with chemotherapy, or one of two increased-dose experimental arms of IMRT with synchronous integrated boost (SIB) in combination with chemotherapy. An integrated translational programme is included across the trial platform.

A total of 711 eligible participants will be recruited from 36 UK National Health Service sites: 90 to ACT3, 162 to ACT4, and 459 to ACT5. Participant pathway is depicted in Figure 1. The protocol (current version 9.0) and this manuscript have been written in accordance with the Standard Protocol Items: Recommendations for Interventional Trials guidelines. Recruitment is anticipated to take place over six years. Recruitment opened on 03/01/2017 and closed on 31/08/23 with a total of 709 patients recruited. Follow up for the primary endpoint analysis of each trial is currently ongoing and the first primary endpoint results are expected to be reported in May 2026.

For peer review only

Trial Objectives

The primary objectives are as follows:

ACT3: to assess 3-year LRF-free rate in participants with anal margin tumours treated with local excision.

ACT4: to assess 3-year LRF- free rate in participants with early-stage disease.

ACT5: to assess LRF-free survival in participants with locally advanced disease.

Secondary Objectives

To assess overall (all ACT trials) and between individual trial treatment arms (ACT4 and 5 only):

- Acute and late toxicities
- Treatment compliance
- Clinical response rate
- Disease-free survival (DFS)
- Colostomy-free survival (CFS)
- Progression-free survival (PFS)
- Overall survival (OS)
- Patient Reported Outcome Measures

Translational Objectives

- To assess the prognostic utility of p16/HPV status and tumour infiltrating lymphocyte scores with respect to chemoradiotherapy in Anal Squamous Cell Carcinoma.
- To investigate the nature and performance characteristics of circulating markers of disease (circulating tumour cells and cell free DNA) in response/relapse in high-risk cases (ACT5 patients only).
- To assess the changes in the gut microbiome caused by chemoradiotherapy and develop hypotheses around any potential relationship between this and patients experiencing late (gut) toxicities.

Study population

Patients with locally excised T1 N0 or Nx anal margin tumour and anal canal superficially invasive squamous cell carcinoma (SISCCA) are eligible for ACT3, patients with T1-2 ≤4cm N0 or Nx anal canal or T2 ≤4cm N0 or Nx anal margin (in situ or treated by prior local excision within 3 months before

For peer review only

randomisation) are eligible for ACT4, and patients with T2 N1-3 or T3-4 Nany are eligible for ACT5. ACT3 accepts patients with an Eastern Cooperative Oncology Group (ECOG) performance status of 0-2 whereas for ACT4 or ACT5 patients require an ECOG performance status of 0-1.

Only participants meeting all the inclusion criteria and none of the exclusion criteria will be considered for participation in the trial, with no eligibility waivers permitted. The full inclusion and exclusion criteria for ACT3, ACT4 and ACT5 are set out in **Table 1** and **Table 2**.

Consent and withdrawal

Informed written consent will be given by all participants before entering the study and before any interventions. An example of the consent form is provided in Supplementary Material 3. Consent will be taken by the Principal Investigator or their approved appropriate medically qualified healthcare professional. Optional consent for participants recruited to ACT5 will be sought regarding the participation in the biological serum plasma biomarker and microbiome sub-studies. In the event of withdrawal, any data collected up until that point will be kept and potentially included in analyses. It will be made clear to any participant withdrawing consent that further data pertaining to safety will continue to be collected.

For peer review only

Registration, randomisation and recruitment

Participants will be registered (ACT3) or randomised (ACT4, ACT5) at the University of Leeds Cancer Research UK Clinical Trials Unit (CTU) following confirmation of written consent and eligibility.

ACT3

Participants with anal tumour margins >1mm (deep and lateral) will undergo observation. Participants with anal tumour margins ≤1mm (close or involved), including piecemeal excision, will receive lower-dose CRT.

ACT4

Participants will be randomised on a 1:2 basis (standard-dose:reduced-dose) to receive either standard-dose IMRT or reduce-doe IMRT, both in combination with chemotherapy. A computer-generated minimisation program that incorporates a random element will be used to ensure the treatment groups are well-balanced for the following participant characteristics:

- T-stage (T1, T2)
- N-stage (N0, NX)
- Gender (M, F)
- HIV status (positive, negative)
- Randomising centre

ACT5

For each of the pilot , Phase II and Phase III trial components, patients who fulfil the eligibility criteria and have given written informed consent will be randomised on a 1:1:1 basis to receive either standard-dose IMRT in combination with chemotherapy, or one of two increased-dose experimental arms of IMRT with synchronous integrated boost (SIB) in combination with chemotherapy and will be allocated a trial number. Acomputer-generated minimisation program will be used to balance the following participant characteristics at randomisation:

- T-stage (T2/3, T4)
- N-stage (NX/0/1, N2/3)
- Gender (M, F)
- HIV status (positive, negative)
- Chemotherapy regimen (5FU, Capecitabine)
- Randomising centre

Sample size

The target sample size is 711 participants across each of the three study arms. The justification for the translational research questions sample size is provided in **Supplemental Material 1**.

ACT3

This study is powered to demonstrate that the overall treatment strategy of local excision alone with or without additional lower-dose radiotherapy with chemotherapy, does not produce unacceptable rates of efficacy (i.e. below 80%), but can reach efficacy rates of up to 90% (i.e. LRF rate of 10%). With 80% power and 5% 1-sided significance, 82 patients are required. Allowing for a drop-out rate of 10%, the target sample size is 90 patients, recruited over 3 years.

For peer review only

Inclusion Criteria		
**ACT3**	**ACT4**	**ACT5**
Informed consent	Informed consent	Informed consent
Histologically-proven, invasive primary squamous cell carcinoma of the anus	Histologically-proven, invasive primary squamous cell carcinoma of the anus	Histologically-proven, invasive primary squamous cell carcinoma of the anus
ECOG performance status 0-2	ECOG performance status 0-1	ECOG performance status 0-1
Absolute neutrophil count >1.5 x 109/L; platelets >100 x 109/L	Absolute neutrophil count >1.5 x 109/L; platelets >100 x 109/L 3	Absolute neutrophil count >1.5 x 109/L; platelets >100 x 109/L
Serum transaminase <2x ULN	Serum transaminase <2x ULN	Serum transaminase <2x ULN
Estimated GFR >50mls/min	Estimated GFR >50mls/min	Estimated GFR >50mls/min
Bilirubin <1.5 x ULN	Bilirubin <1.5 x ULN	Bilirubin <1.5 x ULN
HIV negative or HIV positive and receiving effective antiretroviral therapy with supervision and CD4 count >200	HIV negative or HIV positive and receiving effective antiretroviral therapy with supervision and CD4 count >200	HIV negative or HIV positive and receiving effective antiretroviral therapy with supervision and CD4 count >200
T1 N0 or Nx anal margin tumour and anal canal SISCCA treated with local excision within 3 months before registration	T1-2 ≤4cm N0 or Nx7 anal canal<br>or<br>T2 ≤4cm N0 or Nx7 anal margin (in situ or treated by prior local excision within 3 months before randomisation)	T2 N1-3<br>or<br>T3-4 N any
Age 16 or over	Age 16 or over	Age 16 or over (18 in Ireland)
Patient considered fit for either the protocol-defined follow-up, or reduced dose CRT.	Patient considered fit for all ACT4 protocol defined treatments	Patient considered fit for all ACT5 protocol defined treatments
Prepared to practice methods of contraception of proven efficacy during treatment and until 6 months post end of treatment	Prepared to practice methods of contraception of proven efficacy during treatment and until 6 months post end of treatment	Prepared to practice methods of contraception of proven efficacy during treatment and until 6 months post end of treatment
Able to undergo all mandated staging and follow-up investigations, including MRI	Able to undergo all mandated staging and follow-up investigations, including MRI	Able to undergo all mandated staging and follow-up investigations, including MRI

Table 1: Inclusion criteria for the different PLATO studies. CD = cluster of differentiation, ECOG = Eastern Co-operative Oncology Group, GFR = glomerular filtration rate, CRT = chemoradiotherapy, HIV = human immunodeficiency virus, MRI = magnetic resonance imaging.

For peer review only

Exclusion Criteria		
**ACT3**	**ACT4**	**ACT5**
Previous malignancy of pelvic origin where treatment was completed less than 2 years before registration or there is still evidence of disease, or previous untreated malignancy of any origin.	Previous malignancy of pelvic origin where treatment was completed less than 2 years before randomisation or there is still evidence of disease, or previous untreated malignancy of any origin.	Previous malignancy of pelvic origin where treatment was completed less than 2 years before randomisation or there is still evidence of disease, or previous untreated malignancy of any origin.
Prior systemic chemotherapy for anal cancer	Prior systemic chemotherapy for anal cancer	Prior systemic chemotherapy for anal cancer
Prior radiotherapy to the pelvis	Prior radiotherapy to the pelvis	Prior radiotherapy to the pelvis
Uncontrolled cardiorespiratory comorbidity	Uncontrolled cardiorespiratory comorbidity	Uncontrolled cardiorespiratory comorbidity
Pregnant or lactating	Pregnant or lactating	Pregnant or lactating
Immuncompromised (organ transplant)	Immuncompromised (organ transplant)	Immuncompromised (organ transplant)
Requiring ongoing treatment with a contraindicated concomitant medication	Requiring ongoing treatment with a contraindicated concomitant medication	Requiring ongoing treatment with a contraindicated concomitant medication
>1 local excision performed, at different times, for the same lesion		

Table 2: Exclusion criteria for the different PLATO studies.

For peer review only

ACT4

This study is powered to demonstrate that the reduced-dose IMRT arm does not produce unacceptable rates of efficacy (i.e. below 80%) but can reach efficacy rates of up to 90% (i.e. LRF rate of 10%). A target efficacy rate of 90% or greater is considered desirable based on data from the ACT2 trial [10] where 10% of T1-2 N0 patients had a pelvic failure at 3 years post-randomisation. The standard-dose IMRT arm will act as a calibration arm, to ensure the desired efficacy rate is plausible.

With 80% power and 5% 1-sided significance, 123 patients are required with a 1:2 randomisation (standard-dose:reduced-dose). In order to address the research hypothesis for the p16+ subset, the sample size has been inflated by 20% (as 90% of patients are expected to present with this genotype, and a further 90% have samples suitable for analysis). Allowing for a 10% drop-out rate, the target sample size is 162 patients, recruited over 2 years.

ACT5

The pilot stage of ACT5 will recruit a maximum of 60 patients (20:20:20). A formal power calculation was not performed for the pilot study given the aim is to review the initial feasibility of dose escalation and concomitant chemotherapy.

The Phase II trial will recruit an additional 80 patients. The phase II analysis will focus on the acute toxicity data including grade 3 and above neutropenia from the first 140 patients (including the pilot) randomised (overall in the three arms). With 80% power and 5% 1-sided significance, an acceptable rate of grade 3/4 neutropenia of <40% and an unacceptable rate of ≥60% yields a sample size of 126 patients (42:42:42), 140 with 10% drop-out. The desired rate of 40% is based on data from the RTOG 9811, RTOG 0529 and ACT2 trials where rates of grade 3/4 neutropenia were 45%, 58% and 24% respectively.

The overall trial is powered on detecting an absolute difference of 10% in 3-year LRF-free survival between the control and experimental arm(s). This difference is equivalent to a hazard ratio of 0.63 and assumes a LRF rate of 30% (3-year LRF-free survival 70%) in the control arm based on the ACT2 data [10].

If both experimental arms pass the pre-specified boundaries for acceptable toxicity at interim analysis, all three arms will continue into the phase III trial. This would compare each experimental arm against the standard dose in terms of 3-year LRF-free survival. With 80% power and 10% 2-sided significance 459 (153:153:153) patients are required (including 10% drop out) for the primary endpoint analysis. The target event rate is 113 per comparison based on assuming 6 years of recruitment and a minimum of 3 years follow up for all patients.

Treatment regimen

Radiotherapy treatment outlining and planning

Participants will have their radiotherapy planning study performed using a CT acquisition with a slice thickness of no greater than 3mm in a supine position. Patients will be scanned with a comfortably full bladder using local bladder filling protocol. The clinician will determine if additional bolus material is required during scanning. A nationally approved consensus protocol was developed for IMRT (**Supplementary Material 1)**.

For peer review only

Treatment regimen

ACT3

Participants within the observation arm will be observed under local practice but must attend trial follow-up assessments. Patients under the intervention arm will receive reduced dose IMRT (CTV 41.4Gy in 23 fractions(F)) plus MMC capecitabine (**Supplementary Material 2**).

ACT4

Participants are randomised to receive either to receive either standard-dose IMRT (GTV 50.4Gy, Elective CTV 40Gy (28F)) in combination with chemotherapy or reduced-dose IMRT (GTV 41.4Gy, Elective CTV 34.5Gy (23F)) in combination with chemotherapy (**Supplementary Material 2**)

ACT5

Patients will receive either standard-dose IMRT (GTV 53.2Gy) or one of two increased-dose experimental arms of IMRT with SIB (GTV 58.8Gy or GTV 61.6Gy) with elective CTV treated to 40Gy (28F)) in combination with chemotherapy. ACT5 will use Mitomycin C combined with either Capecitabine or 5 Fluorouracil (5FU) based on hospital preference (**Supplementary Material 2**).

Trial Assessments and follow up

For an overview of the ACT3, ACT4 and ACT5 assessment schedule please see **Supplementary Material 2**.

Clinician reported toxicity

With the exception of the ACT3 observation arm, assessment of acute toxicities will take place during each week of treatment. In ACT5 only, clinician reported acute toxicities will be captured at 6 weeks, 3 and 6 months post-end of treatment. All adverse reactions, serious adverse reactions and related unexpected serious adverse events will be recorded in ACT5, in ACT 4 grade 2 and above will be recorded and in ACT3 grade 3 and above will be recorded.

Patient reported outcome measures (toxicity and quality of life)

Baseline, acute and late toxicities and assessment of participants' Quality-of-life (QoL) will be performed via the validated EORTC QLQ-C30 and QLQ-ANL27 [21] questionnaires at baseline, in the final week of treatment, at 6 weeks, 6, 12, 24 and 36 months post-end of treatment. ACT3 participants in the observation arm will complete QoL questionnaires at 6 weeks, 6, 12, 24 and 36 months post-

For peer review only

date of registration. Patients will all complete baseline and final week of treatment questionnaires on paper and then could opt to complete on paper or online using REDCAP.

Follow up imaging

For ACT3 an MRI pelvis will be carried out at 12 and 36 months post-treatment.

For ACT4 and ACT5 MRI pelvis will be performed at 3 and 6 months, and a follow-up CT (chest/abdomen/pelvis) will be carried out at 12, 24 and 36 months post-treatment.

Assessment of efficacy

All patients will be followed up to detect recurrence that may occur in the pelvis or as distant metastases. These will be recorded on the relevant CRF and completed at the time of each follow-up visit. All failures that occur within the pelvis up to the level of the sacral promontory are considered loco-regional. The date of failure will be taken as the date of the biopsy, or where this is not available, the data of the first imaging assessment that confirmed the LRF. The presence of distant metastases is determined by cross sectional imaging.

DFS is defined as the time from randomisation to the first documented evidence of pelvic failure which includes: LRFs, distant metastases, new primary tumours or death.

All deaths occurring from the date of registration/randomisation will be recorded.

Ancillary Care

Participants will receive ancillary care beyond the scope of the trial as required. Any adverse events or complications arising from trial participation will be promptly addressed, and necessary medical interventions will be provided at no cost to the participants. Ancillary care will extend to managing conditions unrelated to the trial that may arise during the study period, ensuring the overall well-being of the participants.

Translational research

Baseline and pre-treatment biopsies, and resections and biopsies from recurrent disease will be collected retrospectively for participants. Tumours will be characterised for p16 (immunohistochemistry), HPV status (PCR) and tumour infiltrating lymphocyte scores, and correlated with outcome.

For peer review only

Longitudinal blood (plasma) samples (ACT 5) (pre-treatment, end of treatment, 3 months, 6 months and 12 months post treatment and at relapse) will be used for analysis of cell free DNA (cfDNA) and characterisation and changes in levels of circulating tumour cells and comparison of these with conventional markers of response.

At least 65% of the ACT5 participants will have faecal samples collected prior to commencing chemoradiotherapy and a paired sample 6-12 weeks (+/- 4 weeks) after the end of treatment. The microbiome will be characterized in these samples and will allow for additional biological insights into local immune response and potential factors relating to long term bowel toxicity.

Data collection and management

Participant data will be recorded on trial-specific case reports forms and submitted to the CTU from participating sites. All data will be entered, validated and stored securely by CTU with missing and discrepant data queried by the data management team. Missing data will be followed up until received or confirmed not available. Data items regarding consent, patient safety and the primary endpoint will be subject to manual priority checking.

Only the CTU will have access to the data, prior to analysis and release of trial results. On completion of the trial, data will be stored in the sponsor archiving facility for a minimum of 15 years. After the final trial results have been published, interested researchers may contact the CTRU to request access to relevant data.

Statistical Analysis

Efficacy analyses for ACT3 and ACT5 will be on an intention-to-treat (ITT) basis, including participants according to the treatment arm they were recruited/randomised to. For ACT4 a modified ITT will be used, including participants if they receive at least one dose of trial treatment.

<u>Primary endpoint analysis</u>

LRF includes a failure at the primary site (local) and/or surrounding nodal sites (regional). The event date is the biopsy date where the failure was confirmed histologically, or the scan date used to confirm failure on MDT review if a biopsy date is not available.

For peer review only

In ACT3, analysis will assess the proportion of participants without a LRF at 3-year post-recruitment, and the one-sided 95% CI (corresponding to a two-sided 90% CI) on the whole trial cohort. Assessment of the efficacy of the overall treatment strategy will focus on the lower bound of the CI, and whether it falls below the 'unacceptable' rate of 80%.

In ACT4, analysis will assess the proportion of participants without a LRF in the reduced-dose IMRT arm at 3 years post-recruitment and the one-sided 95% CI. Assessment of the efficacy will focus on whether the lower bound of the CI falls below the 'unacceptable' rate of 80%. The proportion of participants without a LRF in the standard-dose IMRT arm with corresponding CI will be presented to act as a calibration arm, and to establish whether the desired efficacy rate of 90% is plausible. In addition, the analysis will be performed on the subgroup of patients with a p16+ genotype.

In ACT5 LRF-free survival for each experimental arm will be compared against the control, i.e. two separate comparisons, using Cox's proportional hazards (PH) modelling, adjusting for the minimisation factors. The hazard ratio (HR) for the experimental arm versus the control arm will be presented along with two-sided 90% CIs and associated p-value testing. A secondary analysis will compare the combined dose escalation arms with the control to test the hypothesis that dose escalation generally provides improved LRF-free survival compared to control.

LRF-free survival will be presented via Kaplan-Meier (KM) curves. Median LRF-free survival and LRF-free survival estimates at 3 years, with corresponding 90% and 95% CIs will be presented along with the log-rank test statistic (and associated p-value).

Participants who are failure-free at the time of analysis, or who have come off trial prior to observing their primary endpoint will be censored at their last known alive and LRF-free date.

For peer review only

Secondary endpoint analysis

Time-to-event endpoints including CFS, DFS PFS and OS will be presented using KM curves. For ACT5 Cox's PH model will be used to compare each experimental arm with the control. There will be no formal comparison between the arms for ACT3 and ACT4. The proportion of participants experiencing each event will be presented by treatment group (where applicable) and overall.

The proportion of participants experiencing acute toxicities as per the latest National Cancer institute Common Terminology Criteria for Adverse Events (NCI-CTAE) will be summarised for each treatment

arm, for the overall treatment period and (for ACT5 only) at 6 weeks and 3 and 6 months. The number and proportion of participants experiencing grade 3 and above acute toxicities will be reported along with 95% CIs. These will be compared between each treatment arm and the control using a multivariable logistic regression model adjusted for the minimisation factors, for ACT5 only. Exploratory comparisons of acute toxicity may be performed between the experimental arms.

The proportion of participants adhering to the radiotherapy schedule (with no greater than 3 days delay due to toxicity) will be reported. In ACT5 only this will be compared between each treatment arm and the control using a logistic regression model.

The proportion of participants achieving a Complete Response (CR) at 3 and 6 months will be presented along with 95% CIs (ACT4 and 5). Complete response is defined as a TRG score of 1 or 2 assessed via MRI imaging, or if clinical assessment only is used to define response, a category of 'Complete response'. A logistic regression model, adjusted for the minimisation factors will be used to compare each treatment arm with the control (ACT 5 only) for the proportion of participants who have and have not achieved a CR at 6 months post-end of treatment.

EORTC QLQ guidelines will be used for analyses and management of missing data.(1) Mean scores and change in mean score from baseline with corresponding 95% CIs will be calculated for all domains of the patient reported questionnaires (EORTC QLQ-C30 and ANL27) for each treatment group and overall, at each follow-up time point.

Descriptive summaries will be presented by treatment arm for the pattern of failure and the proportion of participants with local relapse undergoing salvage surgery.

<u>Translational statistical analysis</u>

Analysis of p16, HPV and LRF

p16/HPV and tumour infiltrating lymphocytes will be analysed as part of the main trial analysis (primary endpoints of 3 year LRF) and as such, results of the biomarker analysis from ACT4 and the pilot/phase II stages of ACT5 will be made available (subject to oversight, see below) with a plan to validate the predictive value in later stages of the platform and inform subsequent biomarker stratification in later stages of the trial.

For peer review only

Analysis of cfDNA

Serial data on CTCs and cfDNA will be analysed for the cohort of patients in ACT5 (with an estimated 25% risk of recurrence at 1 year). Performance characteristics will be determined for these data (CTC and cfDNA) in relation to complete response and 1 year DFS.

Analysis of changes in gut microbiome

The faecal microbiome in patients undergoing radical treatment for anal cancer has not previously been investigated and as such all analyses and comparisons with e.g. long-term toxicity outcome measures are exploratory. Our primary outcome measure is the feasibility of collecting and analysing the faecal microbiome in patients enrolled in ACT5 with respect to changes in microbiome/metabolomics profiles pre- and post-chemoradiotherapy.

Trial oversight

The Chief Investigator (CI) will have overall responsibility for the trial design, set-up and management and will be supported by 5 study-specific clinical Leads across the 3 trials. A trial management group (TMG) comprising of the CI, CTU team, a PPIE representative (LB), co-leads for each trial, representatives for medical oncology, medical imaging, radiotherapy QA, translational research and patient reported outcomes and key external members of staff will meet 3-6 monthly. A trial steering committee will provide overall supervision of the trial, in particular trial progress, adherence to protocol, participant safety and consideration of new information. It will include an Independent Chair, not less than two other independent members and a PPI representative. An independent data monitoring and ethics committee (DMEC), which will meet annually as a minimum, will review safety and ethics of the trial during recruitment and follow-up period.

Patient and Public Involvement and Engagement (PPIE)

Input from the Leeds Cancer Centre PPIE team, National Cancer Research Institute consumer representatives and Leeds Radiotherapy Research user group was sought, and the information gained acted on through every aspect of the protocol design, the patient information leaflet, and the lay summary.

Ethics and dissemination

This study has been approved by the Yorkshire & The Humber - Bradford Leeds Research Ethics Committee (ref: 16/YH/0157, IRAS: 204585), July 2016.

For peer review only

The trial results will be analysed in stages for short-term and long-term endpoints. Results will be disseminated via national and international conferences, peer reviewed journal articles as well as social media and a newsletter to the relevant stakeholders. The final trial publications will be written by the TMG and will adhere to International Committee of Medical Journal Editors guidelines.

Discussion

The incidence of anal carcinoma is rising globally with a range of radiotherapy doses being employed to treat patients without high levels of evidence. PLATO is an integrated umbrella trial which aims to personalise radiotherapy dose for locoregionally confined cancer with arms looking at anal margin tumours, early and advanced anal carcinoma with it being the multi-centre phase II (ACT3 and ACT4) and phase II/III (ACT5) to do so. It is also the first anal cancer trial to use the EORTC QLG-ANL27 questionnaire and has translational components of the study for a subset of patients will add insight into HPV status and circulating tumour markers on disease free survival, and the changes in the gut microbiome and how this may relate to bowel toxicity.

Since the development of the protocol, the core outcome research measures in anal cancer (CORMAC) [22] has been published with the outcomes in the original protocol being retained but the planned analyses will align with the CORMAC recommendations.–PLATO is focussed on personalisation of treatment for patients with a good ECOG performance status and highlights the need for further research into the optimal treatment for elderly and frail patients [23]. As part of this a need for a reliable surrogate for the current performance status which is not subjective and can take into account changes in patients physical state is needed [24]. Medical imaging-based body composition analysis, which is the process of quantifying the volume, density and ratio of adipose tissue, skeletal muscle, bone and soft tissues within the abdomen can provide additional valuable patient-specific metrics which could be used as an indicator or frailty or outcome [25]. Future translational work looking into the imaging biomarkers as part of the PLATO study will be explored and will be invaluable in any future trial design.

Patient consent for publication

Not applicable.

For peer review only

References

[1] Anal cancer incidence statistics (2021) Cancer Research UK. Available at: https://www.cancerresearchuk.org/health-professional/cancer-statistics/statistics-by-cancer-type/anal-cancer/incidence (Accessed: 01 August 2024).

[2] Huang J, Lok V, Zhang L, et alIDDF2022-ABS-0273â€…Global incidence of anal cancer by histological subtypes. Gut 2022;71:A179-A180.

[3] Baricevic I, He X, Chakrabarty B, et al. High-sensitivity human papilloma virus genotyping reveals near universal positivity in anal squamous cell carcinoma: different implications for vaccine prevention and prognosis. Eur J Cancer. 2015;51(6):776-85.

[4] UKCCCR Anal Cancer Trial Working Party. Epidermoid anal cancer: results from the UKCCCR randomised trial of radiotherapy alone versus radiotherapy, 5-fluorouracil, and mitomycin. Lancet. 1996;348(9034):1049-54.

[5] Bartelink H, Roelofsen F, Eschwege F, et al. Concomitant radiotherapy and chemotherapy is superior to radiotherapy alone in the treatment of locally advanced anal cancer: results of a phase III randomized trial of the European Organization for Research and Treatment of Cancer Radiotherapy and Gastrointestinal Cooperative Groups. J Clin Oncol 1997;15:2040-9.

[6] Flam M, John M, Pajaket TF, et al. Role of mitomycin in combination with fluorouracil and radiotherapy, and of salvage chemoradiation in the definitive nonsurgical treatment of epidermoid carcinoma of the anal canal: results of a phase III randomized intergroup study. J Clin Oncol 1996;14:2527-39.

[7] Northover J, Glynne-Jones R, Sebag-Montefiore D, et al. Chemoradiation for the treatment of epidermoid anal cancer: 13-year follow-up of the first randomised UKCCCR Anal Cancer Trial (ACT I). Br J Cancer 2010;30;102(7):1123-8.

[8] Downing A, Morris EJ, Aravani A, et al. The Effect of the UK Coordinating Centre for Cancer Research Anal Cancer Trial (ACT1) on Population-based Treatment and Survival for Squamous Cell Cancer of the Anus. Br J Cancer 2010;102(7):1123-8.

For peer review only

[9] Ajani JA, Winter KA, Gunderson LL, et al. Fluorouracil, mitomycin, and radiotherapy vs fluorouracil, cisplatin, and radiotherapy for carcinoma of the anal canal: a randomized controlled trial. JAMA 2008;299:1914-21.

[10] Gunderson LL, Winter KA, Ajaniet JA, al. Long-term update of US GI intergroup RTOG 98-11 phase III trial for anal carcinoma: survival, relapse, and colostomy failure with concurrent chemoradiation involving fluorouracil/mitomycin versus fluorouracil/cisplatin. J Clin Oncol 2012;30(35):4344-51.

[11] James RD, Glynne-Jones R, Meadows HM, et al. Mitomycin or cisplatin chemoradiation with or without maintenance chemotherapy for treatment of squamous-cell carcinoma of the anus (ACT II): a randomised, phase 3, open-label, 2 x 2 factorial trial. Lancet Oncol 2013;14:516-24.

[12] Peiffert D, Tournier-Rangeard L, Gérard J-P, et al. Induction chemotherapy and dose intensification of the radiation boost in locally advanced anal canal carcinoma: final analysis of the randomized UNICANCER ACCORD 03 trial. J Clin Oncol 2012;30:1941-8.

[13] Muirhead R, Adams RA, Gilbert DC, et al. Anal cancer: developing an intensity-modulated radiotherapy solution for ACT2 fractionation. Clin Oncol (R Coll Radiol) 2014;26(11):720-1.

[14] Muirhead R, Partridge M, Hawkins MA. A tumor control probability model for anal squamous cell carcinoma. Radiother Oncol 2015;116(2):192-6.

[15] Gilbert A, Drinkwater K, McParland L, et al. UK national cohort of anal cancer treated with intensity-modulated radiotherapy: One-year oncological and patient-reported outcomes. Eur J Cancer 2020;128:7-16.

[16] Obermueller T, Hautekiet J, Busto MP, et al. Prognostic value of high-risk human papillomavirus DNA and p16INK4a immunohistochemistry in patients with anal cancer: An individual patient data meta-analysis. Eur J Cancer 2021;157:165-178.

[17] Ang KK, Harris J, Wheeler R, et al. Human papillomavirus and survival of patients with oropharyngeal cancer. N Engl J Med 2010;363(1):24-35.

For peer review only

[18] Wakeham K, Murray L, Muirhead R, et al. Multicentre Investigation of Prognostic Factors Incorporating p16 and Tumour Infiltrating Lymphocytes for Anal Cancer After Chemoradiotherapy. Clin Oncol (R Coll Radiol) 2021;33(10):638-649.

[19] Le JY, Cutts RJ, White I, et al. Next Generation Sequencing Assay for Detection of Circulating HPV DNA (cHPV-DNA) in Patients Undergoing Radical (Chemo)Radiotherapy in Anal Squamous Cell Carcinoma (ASCC). Front Oncol 2020;10:505.

[20] Jones CM, Goh V, Sebag-Montefiore D, Gilbert DC. Biomarkers in anal cancer: from biological understanding to stratified treatment. Br J Cancer. 2017 Jan 17;116(2):156-162

[21] Sodergren SC, Johnson CD, Gilbert A, et al. European Organisation for Research and Treatment of Cancer Quality of Life Group. International Validation of the EORTC QLQ-ANL27, a Field Study to Test the Anal Cancer-Specific Health-Related Quality-of-Life Questionnaire. Int J Radiat Oncol Biol Phys 2023;115(5):1155-1164.

[22] Fish R, Sanders C, Adams R, et al. A core outcome set for clinical trials of chemoradiotherapy interventions for anal cancer (CORMAC): a patient and health-care professional consensus. Lancet Gastroenterol Hepatol 2018;3(12):865-873.

[23] Charnley N, Choudhury A, Chesser P, Cooper RA, Sebag-Montefiore D. Effective treatment of anal cancer in the elderly with low-dose chemoradiotherapy. Br J Cancer. 2005 Apr 11;92(7):1221-5.

[24] Simcock R, Wright J. Beyond Performance Status. Clin Oncol (R Coll Radiol). 2020 Sep;32(9):553-561.

[25] 2.   Shah UA, Ballinger TJ, Bhandari R, et al. Imaging modalities for measuring body composition in patients with cancer: opportunities and challenges. J Natl Cancer Inst Monogr. 2023; 2023(61): 56-67.

Figure Legends

Figure 1: Overview of the participant pathway for ACT3, ACT4 and ACT5

For peer review only

Contributors

Contributors to the conception and design: AG, DG, NLA, SDR, VG, SR, JW, AS, JC, SR, LB, RM, AGR, MH, RA, MH, SB, DS-M. Development of the protocol, trial documents and patient information sheet: AG, DG, NLA, SDR, VG, SR, JW, AS, JC, SR, LB, RM, AGR, MH, RA, MH, SB, DS-M. Writing the manuscript: RF, SB, DS-M Reviewed the manuscript: All authors. All authors read and approved the final manuscript.

The guarantor is Prof David Sebag-Montefiore.

Funding

PLATO is supported by Cancer Research UK [A19121] and Stand Up to Cancer. The study is coordinated by University of Leeds CTRU and sponsored by University of Leeds (GA18/118305). For enquires, trial contact email: plato@leeds.ac.uk. Trial website: https://ctru.leeds.ac.uk/plato.

Competing interests

AGR is supported by the NIHR Manchester Biomedical Research Centre (NIHR203308)

SDR is supported in part by the National Institute for Health and Care Research (NIHR) Leeds Biomedical Research Centre (BRC) (NIHR203331). The views expressed are those of the author and not necessarily those of the NHS, the NIHR or the Department of Health and Social Care.

MH is supported by funding from UCLH National Institute for Health and Care Research Biomedical Research Centre.

RF, AG, DG, NLA, VG, SR, JW, AS, JC, SR, LB, RM, RA, SB, DS-M have no competing interested to declare.

For peer review only

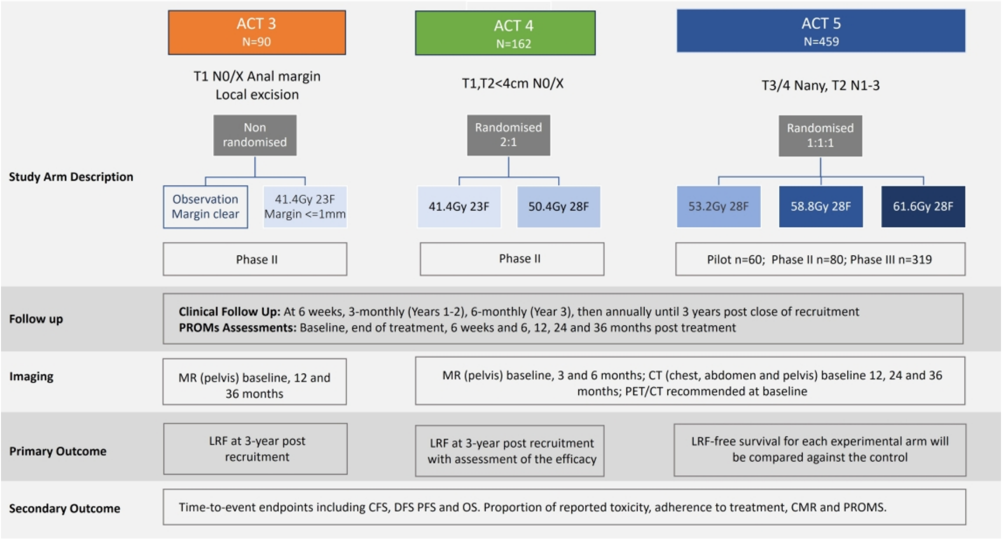

Figure 1: Participant trial pathway

370x199mm (300 x 300 DPI)

Translational research sample size

Analysis of p16, HPV and loco-regional failure

The sample size calculations in ACT4 are inflated to allow for a pre-planned analysis of p16/HPV. With 108 in the dose de-escalation arm of ACT4, we will have around 88% power to the test the dose de-escalation strategy and demonstrate efficacy rates of up to 90%. Assuming only 90% of these patients will have samples suitable for analysis, and 85% will be p16+, we will have 80% power to demonstrate efficacy rates of up to 90% within the p16+ve sub-group. With respect to the ACT5 question around dose escalation, 140 patients in the pilot and phase II stages of ACT5 give approximately 94 patients in the dose escalated arms. Assuming a relapse rate of 30%, where 85% are p16+ve (n=80) and 15% p16-ve (n=14), there would be 94% power to detect a HR of 4.5 between the p16 +ve/-ve groups. With a lower relapse rate of 20% in the dose escalated arms (as targeted within ACT5), the power would reduce to 85%. With 90% of samples suitable for analysis (so 126 patients with samples at the end of phase II, 84 in the dose escalated arm) and assuming a relapse rate of 30%, 85% p16+ve (n=71), and 15% p16-ve (n=13), there would be 90% power to detect a HR of 4.5 between the p16 +ve/-ve groups. If we assumed a relapse rate of 20% in the dose escalated arms (which is what were are targeting in ACT5), the power would reduce to 78%. The power to demonstrate a relationship between TIL score and relapse within p16 +ve patients is about 80% for a relapse rate of 30% and just over 50% for a relapse rate of 20%.

Analysis of cfDNA

Serial data on CTCs and cfDNA (presence/absence and enumeration/ quantification) will be analysed for the cohort of patients in ACT5 (phase II/III) with an estimated 25% risk of recurrence at 1 year. Performance characteristics (including ROC) will be determined for these data (CTC and cfDNA) in relation to complete response and 1 year disease free survival. For such longitudinal monitoring power calculation methodology is less clearly developed, but with 6 marker values throughout the follow-up period on 80 patients we will only be powered to detect relatively large effects.

Analysis of changes in gut microbiome

The faecal microbiome in patients undergoing radical treatment for anal cancer has not previously been investigated and as such all analyses and comparisons are exploratory. Our primary outcome measure is the feasibility of collecting and analysing the faecal microbiome in patients enrolled in ACT5 in >65% cases, i.e. obtaining paired, evaluable samples in at least 65% of patients. If we recruit at least 55 patients into the faecal microbiome sub-study, evaluable paired samples in 42 of these will give us

For peer review only

80% power (at the 1-sided 5% significance level) to achieve this, assuming a true paired, evaluable rate of 80%.

Target volume definition

Gross Tumour Volume (GTV) – ACT3, ACT4 and ACT 5 patients

The GTV will be determined by the treating clinician using the planning CT, and information from clinical data and diagnostic imaging.

- GTV_A = Site of primary tumour and scar (ACT3)
- GTV_A = Includes the gross primary anal tumour volume (ACT4 and ACT5)
- GTV_N = Includes all involved nodes ≤3cm (ACT5)
- GTV_N3 = Includes all involved nodes >3cm (ACT5)

Clinical Target Volume (CTV) – ACT3, ACT4 and ACT5 patients

All the expansions are in 3 dimensions unless stated otherwise.

- CTV_A = GTV_A + 10mm (ACT3)
- CTV_A = GTV_A + 10mm (ACT4)
- CTV_A = GTV_A +15mm (ACT5)

This is manually enlarged to ensure coverage of the anal canal, anal verge, internal and external anal sphincters. In the absence of bony involvement this is then edited to exclude bone.

- CTV_N = GTV_N + 5mm. This is also edited off bone and muscles in the absence of bony involvement (ACT5)
- CTV_E = Elective nodal regions (ACT4 and ACT5).

Planning Target Volume (PTV) – ACT3, ACT4 and ACT5 patients

All the expansions are in 3 dimensions unless stated otherwise.

ACT3 patients:-

- PTV_A= CTV_A + 10mm

ACT4 patients:-

- PTV_A= CTV_A + 10mm

For peer review only

- PTV_E = CTV_E + 5mm

ACT5 patients:-

- PTV_A= CTV_A + 10mm
- PTV_N = CTV_N + 5mm
- PTV_E = CTV_E + 5mm

For peer review only

ACT3 treatment summary

Patients with anal tumour margins >1mm (deep and lateral) will undergo observation; patients with anal tumour margins ≤1mm (close or involved) will receive lower-dose CRT.

ACT3 observation arm (if margin >1mm)

Patients in this arm will be under observation as per local practice but must attend trial follow-up assessments.

ACT3 – Radiotherapy plus MMC capecitabine (if margin ≤1mm)

Treatment	Week																						
	1					2					3					4					5		
	Days 1-5					Days 8-12					Days 15-19					Days 22-26					Days 29-31		
Mitomycin C $12mg/m^2$ iv	•																						
Capecitabine $825mg/m^2$ orally bd Mon-Fri*	• •	• •	• •	• •	• •	• •	• •	• •	• •	• •	• •	• •	• •	• •	• •	• •	• •	• •	• •	• •	• •	• •	• •
Radiotherapy CTV 41.4Gy 23F	•	•	•	•	•	•	•	•	•	•	•	•	•	•	•	•	•	•	•	•	•	•	•

*recommended, but treatment may start on any day of the week.

ACT4 treatment summary

Patients will be randomised on a 1:2 basis (standard-dose: reduced-dose) to receive either standard-dose IMRT in combination with chemotherapy or reduced-dose IMRT in combination with chemotherapy.

ACT4 - Standard-dose IMRT plus MMC capecitabine

Treatment	Week																											
	1					2					3					4					5					6		
	Days 1-5					Days 8-12					Days 15-19					Days 22-26					Days 29-33					Days 36-38		
Mitomycin C $12mg/m^2$ iv	•																											
Capecitabine $825mg/m^2$ orally bd Mon-Fri*	• •	• •	• •	• •	• •	• •	• •	• •	• •	• •	• •	• •	• •	• •	• •	• •	• •	• •	• •	• •	• •	• •	• •	• •	• •	• •	• •	• •
Radiotherapy GTV 50.4Gy CTV 40Gy 28F	•	•	•	•	•	•	•	•	•	•	•	•	•	•	•	•	•	•	•	•	•	•	•	•	•	•	•	•

*recommended, but treatment may start on any day of the week.

ACT4 - Reduced-dose IMRT plus MMC capecitabine

Treatment	Week																						
	1					2					3					4					5		
	Days 1-5					Days 8-12					Days 15-19					Days 22-26					Days 29-31		
Mitomycin C 12mg/m^2 iv	•																						
Capecitabine 825mg/m^2 orally bd Mon-Fri*	• •	• •	• •	• •	• •	• •	• •	• •	• •	• •	• •	• •	• •	• •	• •	• •	• •	• •	• •	• •	• •	• •	• •
Radiotherapy GTV 41.4Gy CTV 34.5Gy 23F	•	•	•	•	•	•	•	•	•	•	•	•	•	•	•	•	•	•	•	•	•	•	•

*recommended, but treatment may start on any day of the week.

ACT5 treatment summary

Patients will receive either standard-dose IMRT or one of two increased-dose experimental arms of IMRT with SIB, both in combination with chemotherapy. ACT5 will use Mitomycin C combined with either Capecitabine or 5 Fluorouracil (5FU).

ACT5 - Standard-dose IMRT plus MMC capecitabine

Treatment	Week																											
	1					2					3					4					5					6		
	Days 1-5					Days 8-12					Days 15-19					Days 22-26					Days 29-33					Days 36-38		
Mitomycin C 12mg/m^2 iv	•																											
Capecitabine 825mg/m^2 orally bd Mon-Fri*	• •	• •	• •	• •	• •	• •	• •	• •	• •	• •	• •	• •	• •	• •	• •	• •	• •	• •	• •	• •	• •	• •	• •	• •	• •	• •	• •	• •
Radiotherapy GTV 53.2Gy CTV 40Gy 28F	•	•	•	•	•	•	•	•	•	•	•	•	•	•	•	•	•	•	•	•	•	•	•	•	•	•	•	•

*recommended, but treatment may start on any day of the week.

ACT5 – SIB1 IMRT plus MMC capecitabine

Treatment	Week																											
	1					2					3					4					5					6		
	Days 1-5					Days 8-12					Days 15-19					Days 22-26					Days 29-33					Days 36-38		
Mitomycin C 12mg/m^2 iv	•																											
Capecitabine 825mg/m^2 orally bd Mon-Fri*	• •	• •	• •	• •	• •	• •	• •	• •	• •	• •	• •	• •	• •	• •	• •	• •	• •	• •	• •	• •	• •	• •	• •	• •	• •	• •	• •	• •

For peer review only

Radiotherapy GTV 58.8Gy CTV 40Gy 28F	•	•	•	•	•	•	•	•	•	•	•	•	•	•	•	•	•	•	•	•	•	•	•	•	•	•	•	•

*recommended, but treatment may start on any day of the week.

ACT5 – SIB2 IMRT plus MMC capecitabine

Treatment	Week																											
	1					2					3					4					5					6		
	Days 1-5					Days 8-12					Days 15-19					Days 22-26					Days 29-33					Days 36-38		
Mitomycin C 12mg/m² iv	•																											
Capecitabine 825mg/m² orally bd Mon-Fri*	• •	• •	• •	• •	• •	• •	• •	• •	• •	• •	• •	• •	• •	• •	• •	• •	• •	• •	• •	• •	• •	• •	• •	• •	• •	• •	• •	• •
Radiotherapy GTV 61.6Gy CTV 40Gy 28F	•	•	•	•	•	•	•	•	•	•	•	•	•	•	•	•	•	•	•	•	•	•	•	•	•	•	•	•

*recommended, but treatment may start on any day of the week.

ACT5 - Standard-dose IMRT plus MMC 5FU

Treatment	Week																											
	1					2					3					4					5					6		
	Days 1-5					Days 8-12					Days 15-19					Days 22-26					Days 29-33					Days 36-38		
Mitomycin C 12mg/m² iv	•																											
5FU 1000mg/m²/24h iv	•	•	•	•																	•	•	•	•				
Radiotherapy GTV 53.2Gy CTV 40Gy 28F Mon-Fri*	•	•	•	•	•	•	•	•	•	•	•	•	•	•	•	•	•	•	•	•	•	•	•	•	•	•	•	•

ACT5 - SIB1 IMRT plus MMC 5FU

Treatment	Week																											
	1					2					3					4					5					6		
	Days 1-5					Days 8-12					Days 15-19					Days 22-26					Days 29-33					Days 36-38		
Mitomycin C 12mg/m² iv	•																											
5FU 1000mg/m²/24h iv	•	•	•	•																	•	•	•	•				

For peer review only

Radiotherapy GTV 58.8Gy CTV 40Gy 28F Mon-Fri*	•	•	•	•	•	•	•	•	•	•	•	•	•	•	•	•	•	•	•	•	•	•	•	•	•	•	•	•

ACT5 – SIB2 IMRT plus MMC 5FU

Treatment	Week																											
	1					2					3					4					5					6		
	Days 1-5					Days 8-12					Days 15-19					Days 22-26					Days 29-33					Days 36-38		
Mitomycin C 12mg/m^2 iv	•																											
5FU 1000mg/m^2/24h iv	•	•	•	•																	•	•	•	•				
Radiotherapy GTV 61.6Gy CTV 40Gy 28F Mon-Fri*	•	•	•	•	•	•	•	•	•	•	•	•	•	•	•	•	•	•	•	•	•	•	•	•	•	•	•	•

*recommended, but radiotherapy may start on any day of the week. Regardless of when radiotherapy starts, MMC and 5FU should start on the first Monday or Tuesday of radiotherapy.

For peer review only

ACT3 Assessment schedule

ACT3	Baseline			Treatment					Follow-up from end of treatment (or date of registration for observational arm)														
	Eligibility assessments	Pre reg	Pre tx	Week 1	Week 2	Week 3	Week 4	Week 5	6 weeks	3 months	6 months	9 months	12 months	15 months	18 months	21 months	24 months	30 months	36 months	48 months	60 months	Then annually[5]	Relapse
Medical history	●																						
ECOG PS, Vital Signs, ht/wt	●[1]																						
Physical exam	●								●*	●	●	●	●	●	●	●	●	●	●	●	●		
Tumour biopsy	●																						
Pregnancy test	●*[1]																						
Full Blood Count:	●[1]		●*[4]	●*	●*	●*	●*	●*															
U&E	●[1]		●*[4]	●*	●*	●*	●*	●*															
LFTs	●[1]		●*[4]	●*	●*	●*	●*	●*															
HIV	●*[2]	●**[2]																					
CD4 count if HIV +ve	●*[2]	●**[2]																					
ECG	●*[1]																						
MRI scan	●[3]												*						*				
Informed consent		●																					
Registration		●																					
QoL		●						●*	● CTRU to administer		● CTRU to administer		● CTRU to administer				● CTRU to administer		● CTRU to administer				
Radiotherapy planning scan		●*																					
Data collection	●	●	●*	●*	●*	●*	●*	●*		●	●	●	●	●	●	●	●	●	●	●	●	●	
CTCAE acute toxicity monitoring				●*	●*	●*	●*	●*															
SAR monitoring and reporting				Monitor during weeks of treatment*																			

Translational research samples (explicit consent required)

Tumour Biopsy	●[6]																						●[6,7]

*RT arm only
** observation arm only
[1]within 14 days prior to registration
[2]within 28 days prior to registration
[3]within 63 days prior to registration
[4]within 10 days prior to start of treatment
[5]until 3 years after the last participant has completed treatment or death
[6]if consented to retrieval of stored material for future cancer research
[7]and where clinically indicated

For peer review only

ACT4 Assessment schedule

ACT4	Baseline			Treatment						Follow-up from end of treatment														
	Eligibility assessments	Pre rand	Pre tx	Week 1	Week 2	Week 3	Week 4	Week 5	Week 6	6 weeks	3 months	6 months	9 months	12 months	15 months	18 months	21 months	24 months	30 months	36 months	48 months	60 months	then annually[6]	Relapse
Medical history	•																							
ECOG PS, Vital Signs, ht/wt	•[1]																							
Physical exam	•									•	•	•	•	•	•	•	•	•	•	•	•	•		
Tumour Biopsy	•																							
Pregnancy test	•[1]																							
Full Blood Count	•[1]		•[5]	•	•	•	•	•	•*															
U&E	•[1]		•[5]	•	•	•	•	•	•*															
LFTs	•[1]		•[5]	•	•	•	•	•	•*															
HIV	•[2]																							
CD4 count if HIV +ve	•[2]																							
ECG	•[1]																							
CT scan	•[3]													•				•		•				
MRI scan	•[4]										•	•												
PET scan	•[4] Strongly recommended																							
Informed consent		•																						
Randomisation		•																						
QoL		•						• 41.4Gy arm only	• 50.4Gy arm only	• CTRU to administer		• CTRU to administer		• CTRU to administer				• CTRU to administer		• CTRU to administer				
Radiotherapy planning scan		•																						
Data collection	•	•	•	•	•	•	•	•	•*		•	•	•	•	•	•	•	•	•	•	•	•	•	
CTCAE acute toxicity monitoring				•	•	•	•	•	•*															
SAR monitoring and reporting				Monitor during weeks of treatment																				
Response assessment											•	•												

Translational research samples (explicit consent required)

Tumour Biopsy	•[7]																							•[7,8]

*50.4Gy arm only

[1]within 14 days prior to randomisation.

[2]within 28 days prior to randomisation

[3]within 63 days prior to randomisation. If the CT scan falls outside this timeframe but a PET CT has been carried out, there is no requirement to repeat the CT scan as long as the PET CT is a whole body scan.

[4]as a minimum, 1 of the MRI or PET scan (if done) for pelvic staging must be within 42 days before randomisation

[5]within 10 days prior to start of treatment

[6]until 3 years after the last participant has completed treatment or death

[7]if consented to retrieval of stored material for future cancer research

[8]and where clinically indicated

For peer review only

ACT5 Assessment schedule

ACT5	Baseline: Eligibility assessments	Baseline: Pre rand	Baseline: Pre tx	Treatment: Week 1	Treatment: Week 2	Treatment: Week 3	Treatment: Week 4	Treatment: Week 5	Treatment: Week 6	Follow-up from end of treatment: 6 weeks	3 months	6 months	9 months	12 months	15 months	18 months	21 months	24 months	30 months	36 months	48 months	60 months	then annually[6]	Relapse
Medical history	•																							
ECOG PS, Vital Signs, ht/wt	•[1]																							
Physical exam	•									•	•	•	•	•	•	•	•	•	•	•	•	•		
Tumour Biopsy	•																							
Pregnancy test	•[1]																							
Full Blood Count	•[1]		•[5]	•	•	•	•	•	•															
U&E	•[1]		•[5]	•	•	•	•	•	•															
LFTs	•[1]		•[5]	•	•	•	•	•	•															
HIV	•[2]																							
CD4 count if HIV +ve	•[2]																							
ECG	•[1]																							
CT scan	•[3]													•				•		•				
MRI scan	•[4]										•	•												
PET scan	•[4] Strongly recommended																							
Informed consent		•																						
Randomisation		•																						
QoL		•							•	• CTRU to administer		• CTRU to administer		• CTRU to administer				• CTRU to administer		• CTRU to administer				
Radiotherapy planning scan		•																						
Data collection	•	•	•	•	•	•	•	•	•	•	•	•	•	•	•	•	•	•	•	•	•	•	•	
CTCAE acute toxicity monitoring				•	•	•	•	•	•	•	•	•												
SAR monitoring and reporting				Monitor during weeks of treatment and until 6 months post end of treatment																				
Response assessment											•	•												

Translational research samples (explicit consent required)

ACT5	Eligibility assessments	Pre rand	Pre tx	Week 1	Week 2	Week 3	Week 4	Week 5	Week 6	6 weeks	3 months	6 months	9 months	12 months	15 months	18 months	21 months	24 months	30 months	36 months	48 months	60 months	then annually	Relapse
Tumour Biopsy	•[7]																							•[7,8]
2 x Streck Tubes Blood (only for participants currently enrolled in the sub-study)	Blood sample sub-study closed to new patients in March 2022. Sample collections only applicable for patients already enrolled.										•[9]	•[9]		•[9]										•[9]
Consent for microbiome sub-study (separate PIS/ICF)		•																						
Stool sample collection			•[10]								•[10]													
Midstream urine collection			•[10]								•[10]													

[1]within 14 days prior to randomisation.
[2]within 28 days prior to randomisation.
[3]within 63 days prior to randomisation. If the CT scan falls outside this timeframe but a PET CT has been carried out within 42 days prior to randomisation, then there is no requirement to repeat the CT scan as long as the PET CT is a whole body scan.
[4]as a minimum, 1 of the MRI or PET scan (if done) for pelvic staging must be within 42 days before randomisation.
[5]within 10 days prior to start of treatment
[6]until 3 years after the last participant has completed treatment or death
[7]if consented to retrieval of stored material for future cancer research
[8]and where clinically indicated
[9]if consented to biomarkers sub-study and after randomisation (ACT5 only)
[10]if consented to gut microbiome sub-study and after randomisation (ACT5 only)

For peer review only

Delete text and insert local header here

Patient Information Sheet and Informed Consent Document

PLATO

Anal Cancer Trial 5 (ACT5)

CANCER RESEARCH UK

A large-print version of this sheet is available on request

We invite you to take part in a research study called ACT5 (Anal Cancer Trial 5)

- Before you decide whether to take part, it is important for you to understand why the research is being done, what it would involve for you and how we would use the information we would collect about you.
- Please take time to read the following information carefully. Talk to others about the study if you wish.
- Ask us if anything is unclear, or if you would like more information.
- Once you have read this information, your doctor or nurse will talk to you about the study again and you can ask any questions you like.

- **Part 1** (Sections 1-22) tells you the purpose of the study and what will happen if you take part.
- **Part 2** (Sections 23-33) gives you more detailed information about the conduct of the study.
- Please take time to decide whether or not you wish to take part.

Thank you for reading this information sheet.

How to contact us

If you have any questions about this study, or need to speak to a member of your medical care team, please contact:

<<Enter PI, nurse name >>

<< Contact details for site>>

In case of emergencies outside of office hours, please contact:

<<Please insert relevant numbers for emergency contacts>>

For peer review only - http://bmjopen.bmj.com/site/about/guidelines.xhtml

Part 1

Important things that you need to know

- We want to find out the best way to treat patients with locally advanced cancer of the anus.
- The study will compare 3 different doses of radiotherapy given with chemotherapy (chemoradiotherapy).
- You will need to attend hospital every week day for treatment (for 5½ weeks), then for follow-up assessments every 3 months (for 2 years) and every 6 months (for 1 year), when we will collect information about your progress.
- You can stop taking part in the study at any time, without giving a reason. This will not affect the standard of care you receive in any way.

1 What is the purpose of the study?

In this study we are exploring the potential benefit of giving higher doses of radiotherapy to the tumour in patients with more advanced anal cancers. An advanced cancer is one that is bigger than 4cm or has spread into the local lymph nodes.

Previous research has shown that patients with a more advanced cancer have a higher chance of the cancer coming back.

We are testing to see if giving a higher dose of radiotherapy reduces the chance of the cancer coming back, while at the same time not causing too many extra side effects.

2 Why have I been chosen?

Your Doctor or Surgeon has informed you that you have a locally advanced anal cancer that may be successfully treated with a combination of chemotherapy and radiotherapy.

The study aims to recruit 459 patients like you.

3 Do I have to take part?

No, your participation in this study is entirely voluntary. If you decide to take part, you will be given this information document to keep. You will be asked to sign a consent form, but you may withdraw your consent to take part at any time and without giving a reason.

If you decide not to take part, your doctor or nurse will be happy to talk through other treatment alternatives with you. The standard of your treatment and care will not be affected in any way if you decide not to take part.

4 If I want to, will I definitely be able to take part?

Unfortunately, no. Although your doctor thinks you might be suitable to take part, they will still need to carry out some tests and ask you some specific questions to make sure you are suitable. These are known as “eligibility screening tests”. Most of these tests would happen as a part of normal clinical care, and you may already have undergone some of them. These tests are described under Section 8.

If the eligibility screening tests show that it is not appropriate for you to take part in this study, your doctor will discuss your other treatment options with you.

For peer review only - http://bmjopen.bmj.com/site/about/guidelines.xhtml

5 What is the current standard treatment?

The standard treatment for locally advanced anal cancer is a combination of chemotherapy (anti-cancer drugs) and radiotherapy (high energy X-rays). The aim of this treatment is to get rid of the cancer.

6 What are the new treatments that are being studied?

The new treatments which are being studied are two higher doses of radiotherapy, given in combination with standard chemotherapy. We want to find out whether a higher dose of radiotherapy offers a better chance of getting rid of the cancer, while not causing unacceptable side effects. The higher doses of radiotherapy being tested take no longer to give each day and are given over the same number of days as the standard dose (5½ weeks). The only difference is that the dose given each day is slightly higher than the standard dose. We expect that the higher doses will lead to an increase in side effects at the time of radiotherapy and afterwards (see Section 11), but it may reduce the number of cancers that come back.

7 Which treatment will I receive if I take part?

We need to make sure the groups of people receiving each treatment are as similar as possible, to make sure that the way each group responds to their treatment is due to the treatment itself rather than some other difference between the groups that we do not know about. The best way of dividing people up into groups is by randomly deciding which treatment each person will receive. This is called 'randomisation.' A computer programme will be used to decide what treatment you will be randomised to receive. Neither you, your doctor nor the study staff will be able to choose which group you will be placed in, or which radiotherapy dose you will receive. In this way, a fair comparison between the treatments can be made.

In this study, participants will be split into three groups. Each group will receive a different dose of radiotherapy

If you are in the control group, you will receive the standard dose of radiotherapy. If you are in the experimental groups, you will receive one of the two higher doses of radiotherapy.

You will have a 2 in 3 chance of receiving a higher dose of radiotherapy and a 1 in 3 chance of receiving the standard dose. You will also be given chemotherapy alongside your radiotherapy treatment. The chemotherapy that you will receive will be the same no matter which dose of radiotherapy you receive. Further details about the treatments are given under Section 8.

Occasionally, some patients will be advised to have a surgical procedure before starting treatment if the medical team think that diarrhoea side effects or control of your bowels will become a problem during the treatment. The surgical procedure will form a stoma (called an ileostomy or colostomy). A stoma is an opening on your abdomen or tummy that allows faeces to bypass your back passage and pass straight into a bag. This approach is considered before treatment in routine practice in the same way as this study. Your doctor will explain more about this procedure if it is relevant to you.

For peer review only - http://bmjopen.bmj.com/site/about/guidelines.xhtml

8 What will happen to me if I take part?

This section describes what will happen to you if you agree to take part in the study.

Routine baseline assessments

You will need to have some tests, examinations and scans of your disease, to make sure that you are suitable for the study. Most of these are routine and include:

- Medical history and physical examination.
- A biopsy (a procedure where a small piece of tissue is removed from the cancer to look at under a microscope).
- Blood tests to check your blood count, kidney and liver function. We will also test you for Human Immuno-deficiency Virus (HIV).
- Scans to assess your disease. This will include a CT scan (which involves exposing you to some radiation) of your chest, abdomen and pelvis, and an MRI scan of your pelvis. Your doctor may also choose for you to have a PET scan of your whole body, which may be considered additional to normal care at some hospitals. More information about these scans is provided later (Section 17).

An assessment of your quality of life

If you agree to take part, we would like you to fill in a questionnaire about your quality of life and how you are feeling. This questionnaire will take about 15 minutes and must be completed before you are allocated to your treatment group.

After these assessments, you will attend hospital for radiotherapy treatment planning, radiotherapy and chemotherapy treatment, periodic scans and other checks to monitor your progress, as described below.

Radiotherapy treatment planning

Before your radiotherapy treatment can start, your treatment will need to be planned. You will need to attend the radiotherapy department for a CT scan. This is in addition to the CT scan that you will have had to diagnose your disease. At this visit, the radiographers may make marks on your skin to make sure the same area is treated at each treatment session. After your planning scan, the physicist and your radiotherapy doctor will then carefully create your personal treatment plan using the CT scan images. This planning may take 2 weeks or more to complete. You will then receive an appointment for your first dose of radiotherapy.

Radiotherapy treatment

High energy X-ray beams are directed at the cancer from outside the body (this is known as external beam radiotherapy). The type of radiotherapy that you will receive is called Intensity Modulated Radiotherapy (IMRT). IMRT is able to target your tumour more precisely so that your tumour receives a very high dose of radiotherapy and normal healthy cells nearby receive a much lower dose. The radiotherapy treatment is given as a series of short, daily treatments using equipment similar to a large X-ray machine. The radiographers will help you to get into the right position on the radiotherapy couch. Once you are in the right position, the staff will leave you alone in the room so that they are not exposed to the radiation. They will watch you carefully either through a window or on a closed circuit television screen. The treatment is painless

For peer review only - http://bmjopen.bmj.com/site/about/guidelines.xhtml

and the machine will rotate around you without touching you over 10-20 minutes. Treatment will occur each weekday for 5½ weeks (28 weekday treatments in total).

Chemotherapy treatment

This is the use of anti-cancer drugs to destroy cancer cells. Chemotherapy used in this study is a combination of two drugs. One of the drugs is given by injection through a vein (intravenously) on the first day of radiotherapy. It is called **Mitomycin C**. The second drug is either a drug called **5FU** (which goes into your vein) or **Capecitabine** (which is given as tablets). 5FU is given on the 1st and 5th week of your radiotherapy for 4 days each time. Capecitabine tablets are taken twice a day each day of radiotherapy. Your hospital will tell you which of these two drugs they will use. Both drugs are equally as good, but different hospitals are used to different drugs so will use what they usually give.

It is important that you tell your doctor if you are taking, have recently taken or might take any other medicines. This is particularly important if you are taking any of the following:

- **allopurinol** (gout medicine)
- **warfarin** (blood-thinning medicine used to treat blood clots)
- **sorivudine** and **brivudine** (anti-viral medicines)
- **phenytoin** (medicine for seizures or tremors)
- **metronidazole** (antibiotic)
- **clozapine** (schizophrenia medicine)

9 How long does treatment go on for?

The chemoradiotherapy treatment is given over 5½ weeks (28 treatments). Occasionally it may go on for slightly longer than this if you experience side effects that mean treatment must be interrupted.

10 The effect of chemoradiotherapy on having children

Men:

Male patients will be required to use barrier contraception during chemoradiotherapy and until 6 months after finishing treatment. It is advised that men avoid having children during this time period unless this is through sperm stored before the treatment started. Whilst some men will be infertile after treatment, many will not.

Women:

It is vital to avoid pregnancy during the treatment and contraceptives should be used throughout. It is important to tell your clinical care team if you are pregnant or become pregnant during treatment, as this will affect your care.

It is likely you will be infertile after the treatment is finished. This means you will lose the ability to have children. However, if you think there is any chance you are pregnant in the future, please speak to your doctor.

Further information about the risks of infertility is provided in Section 11h.

For peer review only - http://bmjopen.bmj.com/site/about/guidelines.xhtml

11 What are the potential side effects of radiotherapy?

If you take part in the study, you must report any problems you have to your study nurse or doctor. There is also a contact number given at the end of this information sheet for you to phone if you become worried at any time.

With intensity modulated radiotherapy treatment (IMRT), there is very little normal tissue in the treatment area. This means that the risk of side effects is lower than non-IMRT techniques, but unfortunately you can still have side effects. As with any external beam radiotherapy, the side effects only affect the part of the body that the radiotherapy treatment is aimed at. Radiotherapy affects people in different ways, so it's difficult to predict exactly how you will react. Some people have only mild side effects, but for others the side effects are more severe. Knowing about the side effects may help you to prepare and manage any problems.

Acute side effects of radiotherapy

The side effects that occur during radiotherapy and for a few weeks after finishing radiotherapy treatment are known as 'acute' side effects. They usually get worse towards the end of treatment and then start to gradually get better a few weeks after treatment has ended, but may take some months to entirely settle back to normal.

You should be aware that the side effects listed below may be worse if you get one of the higher doses of radiotherapy

a) Effects on the skin

It's very likely that your skin will be sore in the area being treated, which is around the anus. There will possibly be other areas affected including the scrotum (the testicles and the skin sack they are held in) in men, the vulval area for women, and in the creases in your groin. This soreness usually starts about 2-3 weeks after treatment begins and clears up 3-6 weeks after it finishes. The skin may become blistered and sore and the area may be quite painful. You can be prescribed painkillers to help with this.

The hospital staff will keep an eye on the area and will advise you how to look after your skin. They will provide you with creams and dressings to use on the skin. It's important that you only use products they recommend.

b) Diarrhoea

During the course of treatment, you may notice changes in how your bowel works. You may experience diarrhoea, mucus or jelly-like discharge and pass more wind. These symptoms can sometimes be reduced by avoiding particular foods. Your doctor or a dietician at the hospital can give you advice about this. Most of the time the diarrhoea can be controlled by tablets, but your team will discuss other options if it becomes very troublesome.

c) Urinary problems

During radiotherapy passing urine may become painful or difficult and it may sometimes be difficult to hold your urine. If this happens, your medical team will treat any infection and may suggest pain killers or other tablets. Very occasionally a fine tube (called a catheter) is put into your bladder to drain the urine to relieve pain or symptoms.

For peer review only - http://bmjopen.bmj.com/site/about/guidelines.xhtml

d) Hair loss (from radiotherapy)

It's common to lose your pubic hair during radiotherapy. The hair should grow back after treatment finishes in most patients, although very occasionally the hair loss is permanent.

e) Extreme tiredness (fatigue)

Fatigue is a common side effect of radiotherapy for anal cancer. The tiredness usually starts around 3-4 weeks after treatment begins and may last for a number of weeks following treatment. Generally by around 8-12 weeks, most people feel their energy levels retuning, although this can be a longer period for some. It is important to pace yourself during this time. Your health care team will be able to give you advice on balancing rest with exercise/activities.

f) Vaginal irritation

The vagina can become irritated and inflamed during radiotherapy. It can be prone to infection, so please let staff know if there is an abnormal discharge or itch. Intercourse may be painful or cause bleeding and it may be advisable to not have intercourse until the skin has healed. Your nurse or radiographer will advise you on this and can offer practical advice to help minimise symptoms.

All these acute side effects usually decrease gradually once the treatment has ended, but it may take some months for skin changes to go back to normal.

Late side effects of radiotherapy

Side effects can also occur months and years after finishing radiotherapy. Some side effects can still be a problem years after the patient has finished treatment. These are known as 'chronic' side effects and are listed below.

You should be aware that the side effects may be increased if you receive one of the higher doses of radiotherapy.

g) Bowel problems

Some people find that the way their bowel works is permanently altered after radiotherapy and occasionally a patient has severe difficulties. Problems that can occur include: more regular bowel motions due to scarring; narrowing of the bowel passage causing difficulty in passing bowel motions; bleeding from the back passage due to new, fragile blood vessels formed as a result of radiotherapy; mucus or jelly-like discharge; an urgent need to go to pass a motion, but nothing comes out; or occasionally patients can lose control of bowel motions completely. These side effects can usually be managed although you may need to take medication. Your healthcare team will be able to advise you to minimise any bowel effects that you experience.

In very rare circumstances patients may require surgery to form a stoma (see end of Section 7). Any requirement would be discussed thoroughly with you and information provided to help inform decision making.

h) Infertility (loss of the ability to have children)

Pelvic radiotherapy is likely to cause infertility in women. This is because the ovaries usually stop producing eggs after radiotherapy and the uterus is less flexible and may not be able to carry a baby to full term. Pelvic radiotherapy may also cause infertility in men. If you are considering having a child in the

For peer review only - http://bmjopen.bmj.com/site/about/guidelines.xhtml

future, it is important to discuss your options with your doctor or nurse before treatment starts. Fertility treatments aimed at helping you to be able to have a child after your treatment has finished can be complicated. The options available to you will depend on your age, whether you already have children or a partner, and how soon your treatment needs to start. Your doctor can refer you to a fertility specialist for further discussion.

Men: Sperm can be stored for use in the future. It is important to talk to your doctor before the treatment starts. During radiotherapy you may still produce sperm, but these could be damaged, resulting in abnormalities in the child. It is therefore very important to use barrier contraception during treatment and for a number of months after treatment has finished.

i) Menopause

Women who are still having periods may find that treatment brings on an early menopause because of the reduction in hormones in the ovaries. This results in infertility and menopausal symptoms such as hot flushes and sweats, which may affect sexual activity. Your doctor or nurse can give you advice on managing menopausal symptoms. It usually takes about three months for the ovaries to stop producing eggs and during this time it is still possible to get pregnant. It is important to use contraception during radiotherapy if you are still of child-bearing age.

j) Vaginal dryness and tightening

Women may develop dryness and narrowing of the vagina after radiotherapy. This may make sexual intercourse difficult or uncomfortable. This can often be avoided with the use of “vaginal dilators” as soon as the soreness has resolved after treatment is finished. These are plastic devices that you put into the vagina twice a week to keep the vagina walls open and supple. You may also need to use a lubricating jelly during sex. Your doctor or specialist nurse can give you more information about this.

k) Bones

During radiotherapy, the radiation goes through the bones of your pelvis including your hips bones. As such, you will be at a slightly increased risk of pain in your pelvic bones, or very occasionally breaking these bones in future. If you are concerned please speak to your doctor about reducing the risk of these side effects.

l) Impotence

Men may become unable to have an erection (impotence) after treatment. It’s important to let your doctor know if this happens to you as there are ways this can be managed.

m) Urinary problems

There is a small risk of difficulties passing water or bleeding from the waterworks after finishing radiotherapy. Please let your doctor know if these are an issue because both can be treated.

n) Skin changes

The skin around the pelvis may look different after any blistering has subsided. It may be a slightly different colour than the surrounding skin, have visible blood vessels in it, or feel slightly thicker. It may be prone to bleeding. This does not usually cause a problem, but if you are worried about it please speak to your medical team. You may experience an itch

For peer review only - http://bmjopen.bmj.com/site/about/guidelines.xhtml

which can last for many months (it may be longer in some patients, although this is rare). Your medical team will advise on topical creams which you can apply to minimise the dryness and sooth the itching.

12 What are the potential side effects of chemotherapy?

a) Anaemia (low red blood cells)

Red blood cells carry oxygen round the body. If they are low, you have less oxygen and can be tired and breathless. The medical team will do weekly blood tests to pick this up and occasionally you may need a drip to give you extra red blood cells (blood transfusion).

b) Bruising and bleeding

Chemotherapy can reduce the number of platelets in your blood. Platelets are cells that help the blood to clot. Tell your doctor if you have any bruising or bleeding you can't explain. This includes nosebleeds, bleeding gums, blood spots or rashes on the skin. The medical team will do weekly blood tests to check platelets. Very occasionally you may require a drip to give you extra platelets (platelet transfusion).

c) Risk of infection

Chemotherapy can reduce the number of white blood cells in your blood. When the number of white blood cells is low, it's called neutropenia. White blood cells help your body fight infection. Neutropenia will make you more likely to get an infection.

Contact the hospital straight away on the contact number you've been given if you have a temperature over 37.5°C (99.5°F), you suddenly feel unwell (even with a normal temperature), or you have symptoms of an infection such as shivering, sweats, sore throat or pain on passing water.

The medical team will do weekly blood tests to check for neutropenia.

d) Nausea and vomiting

You may feel sick (nauseous) or be sick (vomiting). Medicines are given to counteract this and are generally very effective. If you continue to suffer nausea or vomiting consult your doctor or nurse, as there are many different anti-sickness medicines available.

e) Hair loss (from chemotherapy)

While the radiotherapy will cause pubic hair loss, some chemotherapy drugs can cause temporary hair loss in all areas of hair. However, this is uncommon with the drugs you will receive in this study.

f) Sore mouth

Chemotherapy drugs may make your mouth sore and cause mouth ulcers. Regular mouthwashes are important and your nurse will show you how to use these properly. Let your medical team know if your mouth continues to be sore as other treatments are available.

g) Severe skin reaction

Although rare, you may develop a severe skin reaction, such as skin rash, ulceration and blistering, which may involve ulcers of the mouth, nose, genitalia, hands, feet and eyes (red and swollen eyes). **Contact the hospital straight away should any of these symptoms occur.**

For peer review only - http://bmjopen.bmj.com/site/about/guidelines.xhtml

13 Very rare side effects of chemotherapy or radiotherapy

There is a very small risk of developing other side effects as a result of receiving chemotherapy. These can include abnormalities in your blood tests, heart problems or blood clots. Please let the medical team know if you have any concerns about any of these.

Giving radiotherapy or chemotherapy, whether given as part of standard treatment or in a study, carries a very small risk of severe side effects that can rarely result in the death of the patient. This risk should be discussed with your doctor and compared with the need for treatment and its possible benefits. Early treatment of side effects is important and you should inform the clinical team promptly if you experience side effects.

14 What if the treatment doesn't help?

Occasionally the treatment does not completely get rid of the cancer. If this happens your team will discuss the options with you. One option is to undergo a surgical operation, which would remove the anus and rectum and leave you with a permanent stoma (see end of Section 7).

15 How is my condition monitored?

You will be seen weekly throughout your chemoradiotherapy treatment and will be able to call for advice at any time.

Once your treatment is complete you will be under close follow up every few months with your cancer team for a total of 3-5 years.

16 What happens when treatment has finished?

After your treatment has finished, you will need to attend clinic for follow-up visits at 6 weeks after your treatment ends, then every 3 months for 2 years, every 6 months for 1 year, then once per year, until 3 years after the trial closes to recruitment. During these study visits, your doctor will examine you and record information about your progress.

You will have an MRI scan of your pelvis at 3 months and 6 months after the end of your treatment. Also, at 12, 24 and 36 months after the end of your treatment you will have a CT scan of your chest, abdomen and pelvis.

We would like you to complete a questionnaire about your quality of life (similar to the one you filled in at the start of the study) at the end of your treatment and again at 6 weeks, 6, 12, 24 and 36 months later. The questionnaire can be completed on-line, or on paper if you prefer.

Your doctor may wish to see you at other time points outside these study visits.

We will register your name, date of birth and, depending on which country you live in, your NHS number[1], CHI number[2], or H&C number[3] with NHS Digital or other registries. This is so we can continue to check your health status for the purposes of this study after you have stopped attending for your study visits.

[1] England and Wales, [2] Scotland, [3] Northern Ireland

17 What are the different scans that I will have?

CT scan

For peer review only - http://bmjopen.bmj.com/site/about/guidelines.xhtml

A computed tomography (CT scan) is a standard test used to create images of the inside of the body using X-rays. It is performed whilst you are lying down as you pass through a tunnel. Please ask your doctor if you wish to know more about this.

PET scan

A positron emission tomography (PET scan) involves a specialist scanner to create images of the inside of your body, which highlight areas of cancer. You have an injection of a very small amount of a radioactive drug (tracer) first. The amount of radiation is very small and does not make you feel unwell. It only stays in the body for a few hours. Please ask your doctor if you wish to know more about this.

MRI scan

A magnetic resonance imaging (MRI scan) uses strong magnetic fields to create images of the inside of your body. It is performed whilst you are lying down as you pass through a tunnel. It is a bit noisy and occasionally people can find it a little claustrophobic. Please ask your doctor if you wish to know more about this.

18 What risk is involved with scans I will have as part of the study?

CT and PET scans involve X-rays (radiation). For many patients, the number of scans and radiation exposure you will receive in this study are the same as if you were not in the study. For some patients, the number of scans and radiation exposure you will receive in this study may be more than you would usually receive. Each scan corresponds to about 5 years' exposure to the natural background radiation that we all receive throughout life.

19 What are the possible benefits of taking part?

We hope that taking part in this study will help you by giving you the best available treatment for your cancer. If you receive one of the higher doses of radiotherapy, it may reduce the chance of the cancer coming back.

20 What are the possible disadvantages and risks of taking part?

Although similar high doses of radiotherapy have been used in other trials done in other countries, the side effects that occur years after radiotherapy have not been studied. There is a risk that the side effects may be worse with the higher doses of radiotherapy, and require more treatment.

21 What if something goes wrong?

As with any cancer treatment, your doctors and nurses aim to ensure that any risks are kept to a minimum. The treatment is checked by a number of different people in every department before and during treatment, so the chance of it being delivered incorrectly is very small. Should this happen, or an unexpected problem arises during treatment your medical team will discuss the consequences and options with you.

The Trial Management Group and independent committees will monitor the study on an on-going basis so that if any of the treatments turn out to be much worse than the others, this will be detected as soon as possible and the study stopped. If you

For peer review only - http://bmjopen.bmj.com/site/about/guidelines.xhtml

experience problems, you must report these to your study nurse or doctor. Their contact numbers can be found at the end of this information sheet.

22 Will my taking part be kept confidential?

If you decide to take part in this study, the information collected about you will be handled strictly in accordance with the General Data Protection Regulation (GDPR) and 2018 Data Protection Act.

The study is being carried out in the United Kingdom and the University of Leeds is the Sponsor for this study. We will be using information from you and your medical records in order to undertake this study. The University of Leeds will act as the data controller for this study. As a publicly-funded organisation, we have to ensure that it is in the public interest when we use personally-identifiable information from people who have agreed to take part in research. This organisation will be responsible for looking after your information and using it properly. The University of Leeds will keep identifiable information about you for 15 years after the study has finished.

Your rights to access, change or move your information are limited, as we need to manage your information in specific ways in order for the research to be reliable and accurate. If you withdraw from the study, we will keep the information about you that we have already obtained. To safeguard your rights, we will use the minimum personally-identifiable information possible.

The University of Leeds will collect information about you for this research study from the hospital you are receiving treatment from. This information will include your name, date of birth and, depending on which country you live in, your NHS number, CHI number, or H&C number, as well as your postal or email address (if you consent to the quality of life study). This information is regarded as a special category of information. The reasons why we collect this are explained in Section 27 below.

If you have any concerns about the way your personal data is being processed or have a query about the information in this document, please contact the University of Leeds Data Protection Officer using any of the following details:

- Email: DPO@leeds.ac.uk;
- General postal address: University of Leeds, Leeds LS2 9JT, UK;
- Postal address for data protection issues: University of Leeds, Room 11.72, EC Stoner Building, Leeds, LS2 9JT;
- Telephone number: +44 (0)113 243 1751.

The data controller registration number provided by the Information Commissioner's Office is Z553814X.

If you are not satisfied with our response or believe we are processing your personal data in a way that is not lawful you can complain to the Information Commissioner's Office (https://ico.org.uk/).

Please refer to Section 27 in Part 2 for further details about how your personal data will be handled.

For peer review only - http://bmjopen.bmj.com/site/about/guidelines.xhtml

This completes Part 1 of the Information Sheet. If the information in Part 1 has interested you and you are considering participation, please continue to read the additional information in Part 2 before making any decision.

For peer review only

For peer review only - http://bmjopen.bmj.com/site/about/guidelines.xhtml

Part 2

23 Additional research (optional)

We will be using this opportunity to study a lot of different aspects of anal cancer. We aim to use information from you to allow us to gain a better understanding of who is at risk of this type of cancer and who is most likely to develop significant side effects. Additional research includes:

1) With your permission, we would like to collect some of your stored biopsies (samples of tumour) from your diagnosis and if your disease relapses. These samples will be sent to a central laboratory and stored for future research within, or possibly outside, the UK. These studies may include genetic tests. The samples will be considered a gift. For this research, your samples will not have your name or address attached to them. They will be labelled with a code number that will enable the trials team to link the sample back to you, but you will not be identified or contacted.

2) We would also like to collect scans taken as part of the study to look at other aspects of anal cancer. These scans will be sent to a central department and held in a secure NHS server. They will be considered a gift. These scans will be used for future research within or possibly outside the UK. In this research, your scans will not have your name / address or details attached to them but the trials team will be able to link them back to you.

It is important to note that collection of these samples and scans and participation in this additional research is optional. Even if you choose to enter the main part of the study, you may choose not to provide these samples. This will not affect your participation in this study, your treatment, or your relationship with your doctor.

24 What if relevant new information becomes available?

Sometimes during the course of a study, new information becomes available. You will be informed if new information comes to light regarding any of the study treatments or your cancer that could have a bearing on the treatment of your cancer. We will notify you of any changes in the way the study is run at the earliest opportunity and discuss with you whether you want to continue in the study. If you decide not to continue, your cancer team will continue with your care as appropriate, and discuss other treatment options with you. If you decide to continue you may be asked to sign an updated consent form. Occasionally on receiving new information, your cancer team may consider it to be in your best interest to withdraw you from further study treatment.

25 What will happen if I don't want to carry on with the study?

You may withdraw from the study at any time. You will not need to give a reason, however, we would like to know the reason if you are willing to say. Before deciding to stop, you should talk to your study doctor or nurse. They can advise you and may be able to deal with any concerns you may have. Information about your participation will remain confidential.

For peer review only - http://bmjopen.bmj.com/site/about/guidelines.xhtml

Even if you do not wish to continue with study treatment, we would still like you to attend for trial visits and assessments and to continue to collect information about you and how you are feeling, unless you request otherwise.

If you do withdraw consent for study treatment and/or further data collection, your data collected up until that point will remain on file and will be included in the final study analysis.

26 Harm and complaints

Every care will be taken in the course of this clinical study. However, in the unlikely event that you are injured as a result of the managing organisation (University of Leeds), compensation may be available but you may have to pay your related legal costs. Your hospital where you receive your treatment has a duty of care to you whether or not you agree to take part in the study and the University of Leeds accepts no liability for negligence on the part of your hospital's employees. If you wish to complain about any aspect of the way you have been treated please contact your research doctor in the first instance.

Any claims will be subject to UK law and must be brought in the UK.

Regardless of this, if you wish to complain, or have any concerns about any aspect of the way you have been approached or treated during the course of this study, the normal National Health Service complaints services are available to you. These are unique to your local NHS Trust. Your study nurse/doctor can give you their information.

If you have private medical insurance, you should contact your insurer, who will let you know whether participation in this study will affect your policy.

27 Will my taking part be kept confidential?

If you decide to take part in ACT5, the information collected about you will be handled in accordance with the General Data Protection Regulation (GDPR) and 2018 Data Protection Act. The information needed for study purposes will be collected on paper forms and sent (usually using standard Royal Mail post, but in some cases by fax, email or secure electronic transfer), from the hospital to the University of Leeds Clinical Trials Research Unit (CTRU). Paper records will be stored in locked filing cabinets. You will be allocated a study number, which will be used along with your date of birth and initials to identify you on each paper form. Your full name and signature will be included on your consent form, and a copy of this will be sent to the CTRU by fax, post or secure electronic transfer. In addition, your full name and postal address or email address will be sent to the CTRU by fax, post or secure electronic transfer to allow the CTRU to issue Quality of Life questionnaires online or by post. Every effort will be made to ensure that any further information about you that leaves the hospital will have your name and address removed so that you cannot be recognised from it. This information will usually be removed by a member of the study team at your hospital, but if this does not happen, it will be removed by the CTRU upon receipt.

If you agree to use the online system (called REDCap) to complete your Quality of Life questionnaires, the CTRU will email a you a

For peer review only - http://bmjopen.bmj.com/site/about/guidelines.xhtml

link to the online questionnaire when it is due to be completed.

When completing the online questionnaires, we recommend using a trusted personal computer/device and avoiding using shared public computers such as the ones in public libraries, as they can be less secure and more open to viruses.

Your GP, and the other doctors involved in your clinical care, will be kept informed of your participation in this study, but otherwise all information about you and your treatment will remain confidential.

We will register your name, date of birth and, depending on which country you live in, your NHS number, CHI number, or H&C number with NHS Digital or other registries. This is so we can continue to check your health status for the purposes of this study after you have stopped attending for your study visits.

Your data will be entered onto a secure database held at the CTRU in accordance with the 2018 Data Protection Act.

Your anonymised data may be passed to other organisations (possibly in other countries where the data protection standards and laws are different to the UK) to monitor the safety of the treatment(s) that you are receiving; this data will have your name removed.

Some of the scans from selected participants may be sent to other hospitals to be looked at by other doctors. This is to ensure that results / reports are consistent across hospitals. These will be sent via standard hospital processes (such as Royal Mail or courier). This data will be anonymised and your name removed.

Your healthcare records may be looked at by authorised individuals from the research team, the University of Leeds (the study Sponsor), or the regulatory authorities, to check that the study is being carried out correctly.

The information collected about you may be shared with other research teams to answer new research questions in future. These researchers may be in this or other organisations. These organisations may be universities, NHS organisations or companies involved in health and care research in this country or abroad. Your information will only be used by organisations and researchers to conduct research in accordance with the UK Policy Framework for Health and Social Care Research. Wherever possible, information will be anonymised. Where this information could identify you, the information will be held securely with strict arrangements about who can access the information. The information will only be used for the purpose of health and care research, or to contact you about future opportunities to participate in research. It will not be used to make decisions about future services available to you, such as insurance.

Where there is a risk that you can be identified your data will only be used in research that has been independently reviewed by an ethics committee.

When the study is finished, the results will be published in a medical journal, but no individual participants will be identified.

In line with Good Clinical Practice guidelines, at the end of the study, your data will be securely archived for a minimum of 15 years. Arrangements for confidential destruction will then be made.

For peer review only - http://bmjopen.bmj.com/site/about/guidelines.xhtml

28 What will happen if I lose mental capacity during the study period?

This is expected to be a very rare occurrence. It could happen to any patient whether or not they take part in this study. If this did occur, no further study treatment would be given and data collection for the study would stop. Data collected up until this point will remain on file and will be included in the study analysis.

29 What happens when the research study stops?

The study will stop three years after the last patient is randomised. After this time the frequency at which you are seen by your doctor may vary slightly depending on your hospital's policy, but you are likely to be seen either 6 monthly or yearly until it is 5 years after your treatment ended.

Five years after your treatment you are usually discharged from follow up, but can always contact the team if you have any concerns following discharge.

30 What will happen to the results of the research study?

When the study is complete, the results will be made openly available to the scientific community and will be published in a medical journal. Your personal information will be protected and kept confidential at all times and no individual participants will be named in any published material. If you would like to obtain a copy of the published results, please ask your doctor. We will prepare a summary which will be available to patients when submitting the results to a journal to make it easier to understand.

31 Who is organising and funding the research?

This study is funded by the Cancer Research UK (CRUK) Clinical Trials Advisory and Awards Committee (CTAAC). The study is being organised by the University of Leeds, through the Clinical Trials and Research Unit (CTRU), who will collect and analyse your data. The study is also sponsored by the University of Leeds.

32 Who has reviewed the study and who will monitor it?

The study was reviewed and approved by the National Cancer Research Institute Colorectal Clinical Studies Group, the NHS Health Research Authority and the Research and Development (R&D) department at your hospital. It has also been reviewed by an independent NHS Research Ethics Committee.

The study is supervised by two independent committees called the Data Monitoring and Ethics Committee (DMEC) and the Trial Steering Committee (TSC). These committees include experienced cancer doctors, statisticians and a patient representative.

33 Further information

If you have any further questions about your cancer or clinical trials, please discuss them with your doctor. You may also find it helpful to contact Macmillan Cancer Support, an independent cancer information charity (free phone: 0808 808 0000; address: 89 Albert

For peer review only - http://bmjopen.bmj.com/site/about/guidelines.xhtml

Embankment, London, SE1 7UQ; web site www.macmillan.org.uk), or CancerHelp.

CancerHelp is an information service Cancer Research UK (Tel: 020 7061 8355; website www.cancerhelp.org.uk). If you would like further information about clinical research, the UK Clinical Research Collaboration (a partnership of organisations working together on clinical research in the UK) have published a booklet entitled 'Understanding Clinical Trials'. Contact UKCRC: Tel: 0207 670 5452; website www.ukcrc.org

An on-line tool that explains some of the terminology used in this information sheet is available on the CancerHelp website http://www.cancerresearchuk.org/cancer-help/utilities/glossary/.

Thank you very much for taking an interest in this research

For peer review only - http://bmjopen.bmj.com/site/about/guidelines.xhtml

Glossary of terms

Short term	Long term	What it means
Anaemia		A low level of red blood cells and/or haemoglobin in your blood. Haemoglobin carries oxygen around the body. It can make you feel very tired, weak or breathless.
Chemoradiotherapy		A combination of chemotherapy and radiotherapy treatment. Chemotherapy is given on the same days as radiotherapy
CT scan	Computer Tomography scan	Using X-Rays to create a 2D picture of inside the body. The image produced is very detailed.
CTRU	Clinical Trials Research Unit	This is the organisation coordinating the study. It is part of the University of Leeds.
IMRT	Intensity Modulated Radiotherapy	This is a way of giving radiotherapy to a tumour in a way that targets the treatment very accurately, and where the normal healthy cells nearby receive a much lower dose.
MRI scan	Magnetic Resonance Imaging scan	Using a magnetic field to create a picture of inside the body. This is very useful for telling the difference between muscle, soft tissue and bone.
Neutropenia		A low level of neutrophils, a type of white blood cell. Neutrophils help the body fight infection. People who have neutropenia have a higher risk of getting serious infections.
PET scan	Positron Emission Tomography scan	Using a scan to detect radiation given off by a small amount of a radioactive drug (tracer) that is injected into you before the scan. It shows how the body tissues are working and what they look like. It is often combined with a CT scan to create more detailed pictures.
Randomisation		Using a computer programme to decide which treatment a patient will. In this way, a fair comparison between the treatments can be made
Stoma		An opening on your abdomen or tummy that allows faeces to bypass your back passage and pass straight into a bag.

For peer review only - http://bmjopen.bmj.com/site/about/guidelines.xhtml

Summary of your participation in ACT5

	Routine Procedures	Extra Procedures
**Eligibility and Consent**	• Physical exam<br>• Medical history<br>• Diagnostic biopsy<br>• Blood tests<br>• Pregnancy test<br>• CT scan<br>• MRI scan	• PET CT scan may be done. If done, this may be routine or extra, depending on your hospital's local policy.
**Randomisation**	**14 days before treatment:**<br>• Heart test (ECG)<br>**10 days before treatment:**<br>• Blood tests	• Questionnaire
	You will be randomised to receive standard-dose radiotherapy plus chemotherapy, or one of two higher doses of radiotherapy plus chemotherapy	
**Radiotherapy planning**	• CT scan to plan your radiotherapy treatment	
**Radiotherapy and chemotherapy 5½ weeks (28 weekday treatments)**	**Each week:**<br>• Blood tests<br>• Assessment of side effects	
**End of Treatment**	• Blood tests<br>• Assessment of side effects	• Questionnaire
**6 weeks after end of treatment**	• Assessment of side effects	• Questionnaire<br>• Physical exam
**Follow Up: Every 3 months for 2 years, then every 6 months for at least 1 year**	• Physical exam<br>• Assessment of side effects at 3 and 6 months after the end of treatment<br>• MRI scan at 3 and 6 months after the end of treatment<br>• CT scan at 12, 24 and 36 months after the end of treatment	• Questionnaire at 6, 12, 24 and 36 months after the end of treatment

For peer review only - http://bmjopen.bmj.com/site/about/guidelines.xhtml

(Reprint this form on headed paper)

Participant ID:	Initials:
Date of Birth:	NHS/CHI/H&C Number:
ISRCTN: 88455282	Principal Investigator:

ACT5 (Anal Cancer Trial 5)

CONSENT FORM

1. I confirm that I have read and understand the information sheet for the above study and have had the opportunity to ask questions.

2. I understand that my participation is voluntary and that I am free to withdraw at any time, without giving any reason and without my medical care or legal rights being affected. I understand that even if I withdraw from the above study, the data collected from me will be used in analysing the results of the study and in some cases, further information about any unwanted effects of my treatment may need to be collected by the study team.

3. I understand that my healthcare records may be looked at by authorised individuals from the study team, regulatory bodies or Sponsor in order to check that the study is being carried out correctly.

4. I understand that the information held and maintained by NHS Digital and other central UK registries may be used to help contact me or provide information about my health status. I give permission for my details (which will include my name, date of birth and NHS number, CHI number, or H&C number) to be submitted to these bodies so that information about my health status may be obtained by the CTRU if necessary.

5. I agree to a copy of this Consent Form being sent to the CTRU.

6. I agree that my general practitioner (GP), or any other doctor treating me, will be notified of my participation in this study.

7. I agree to allow any information or results arising from this study to be used for healthcare and/or further medical research upon the understanding that my identity will remain anonymous.

8. I understand that if during this study my clinical care team determine that I have lost my ability to make my own decisions, no further study intervention will be given. I agree that information collected up until this point will remain on file and will be included in the analysis.

9. I agree to take part in this study.

For peer review only - http://bmjopen.bmj.com/site/about/guidelines.xhtml

The following points are OPTIONAL. Even if you agree to take part in this study, you do not have to agree to the questions in this section.

Please tick ✓

	Yes	No
10. I agree to take part in the Quality of Life study and understand that my email address or postal address will be passed to the CTRU for the purpose of issuing the study Quality of Life questionnaires (either online, or via the post)	☐	☐
11. I give permission for surplus samples from my cancer to be retrieved from my hospital's pathology laboratory and sent to a central laboratory, to be stored and used for future cancer research that receives ethical approval. I understand that my name will not be stored on the samples, but they will be labelled with a unique reference number that will allow the sample to be linked back to me in future, for research purposes. I understand that my tissue samples are a 'gift' and may be shared on a collaborative basis with researchers in the UK and potentially abroad, including outside the European Economic Area. I understand that my tissue samples may be used for genetic research.	☐	☐
12. I give permission for any scans taken as part of the study to be sent to a central department and held in a secure NHS server. They will be considered a gift. These scans will be used for future research that receives ethical approval within or possibly outside the UK. In this research, your scans will not have your name / address or details attached to them but the trials team will be able to link them back to you.	☐	☐

For peer review only

For peer review only - http://bmjopen.bmj.com/site/about/guidelines.xhtml

Patient:

Signature……………………………………………………………………………………

Name (block capitals)……………………………………………………………………

Date…………………………………………………………………………………………

Investigator:

I have explained the study to the above named patient and he/she has indicated his/her willingness to participate.

Signature……………………………………………………………………………………

Name (block capitals)……………………………………………………………………

Date…………………………………………………………………………………………

(If used)Translator:

Signature……………………………………………………………………………………

Name (block capitals)……………………………………………………………………

Date…………………………………………………………………………………………

(1 copy for patient; 1 copy sent to CTRU; 1 copy for patient notes, original stored in Investigator Site File)

For peer review only

For peer review only - http://bmjopen.bmj.com/site/about/guidelines.xhtml
